# The type I-E CRISPR-Cas system influences the acquisition of *bla*_KPC_-IncF plasmid in *Klebsiella pneumonia*

**DOI:** 10.1080/22221751.2020.1763209

**Published:** 2020-05-20

**Authors:** Ying Zhou, Yu Tang, Pan Fu, Dongxing Tian, Lianhua Yu, Yunkun Huang, Gang Li, Meng Li, Yong Wang, Zehua Yang, Xiaogang Xu, Zhe Yin, Dongsheng Zhou, Laurent Poirel, Xiaofei Jiang

**Affiliations:** a Department of Laboratory Medicine, Huashan Hospital, Shanghai Medical College, Fudan University, Shanghai, People’s Republic of China; b Department of Laboratory Medicine, Shanghai Chest Hospital, Shanghai Jiao Tong University, Shanghai, People’s Republic of China; c Department of Laboratory Medicine, Taizhou Municipal Hospital, Taizhou, People’s Republic of China; d Department of Laboratory Medicine Kunming Yan’an Hospital, Kunming, People’s Republic of China; e Department of Laboratory Medicine, Jinshan Hospital, Shanghai Medical College, Fudan University, Shanghai, People’s Republic of China; f Department of Clinical Laboratory, The First Affiliated Hospital of Guangxi Medical University, Nanning, People’s Republic of China; g Department of Clinical Laboratory, Shandong Provincial Hospital affiliated to Shandong University, Jinan, People’s Republic of China; h Department of Laboratory Medicine, Sixth Hospital of Shanxi Medical University, Taiyuan, People’s Republic of China; i Institute of Antibiotics, Huashan Hospital, Fudan University, and Key Laboratory of Clinical Pharmacology of Antibiotics, National Health and Family Planning Commission, Shanghai, People’s Republic of China; j National Clinical Research Center for Aging and Medicine, Huashan Hospital, Fudan University, Shanghai, People’s Republic of China; k State Key Laboratory of Pathogen and Biosecurity, Beijing Institute of Microbiology and Epidemiology, Beijing, People’s Republic of China; l Emerging Antibiotic Resistance Unit, Medical and Molecular Microbiology, Faculty of Science and Medicine, University of Fribourg, Fribourg, Switzerland; m Laboratoire Europeen Associé (LEA) INSERM, IAME (Paris, France), University of Fribourg, Fribourg, Switzerland

**Keywords:** CRISPR-Cas, *Klebsiella pneumoniae* clonal complex 258, carbapenem resistance, plasmids, horizontal gene transfer

## Abstract

*Klebsiella pneumoniae* carbapenemase (KPC)-producing *K. pneumoniae* (KPC-KP) have disseminated worldwide and emerged as major threats to public health. Of epidemiological significance, the international pandemic of KPC-KP is primarily associated with CG258 isolates and *bla*_KPC_-IncF plasmids. CRISPR-Cas system is an adaptive immune system that can hinder gene expansion driven by horizontal gene transfer. Because of *bla*_KPC_-IncF plasmids are favored by CG258 *K. pneumoniae,* it was of interest to examine the co-distribution of CRISPR and *bla*_KPC_-IncF plasmids in such isolates. We collected 459 clinical *K. pneumoniae isolates* in China and collected 203 global whole-genome sequences in GenBank to determine the prevalence of CRISPR-Cas systems. We observed that CRISPR-Cas system was significantly scarce in the CG258 lineage and *bla*_KPC_-positive isolates. Furthermore, the results of conjugation and plasmid stability assay fully demonstrated the CRIPSR-Cas system in *K. pneumoniae* could effectively hindered *bla*_KPC_-IncF plasmids invasion and existence. Notably, most *bla*_KPC_-IncF plasmids were also proved to be good targets of CRISPR owing to carry matched and functional protospacers and PAMs. Overall, our work suggests that type I-E CRISPR-Cas systems could impact the spread of *bla*_KPC_ in *K. pneumoniae* populations, and the scarcity of CRISPR-Cas system was one of potential factors leading to the propagation of *bla*_KPC_-IncF plasmids in CG258 *K. pneumoniae*.

## Introduction

Since they were first identified in 2001 [1], *Klebsiella pneumoniae* carbapenemase (KPC)-producing *K. pneumoniae* (KPC-KP) have emerged as important nosocomial pathogens and causes of global public health concern because of their prevalence and the associated high rate of mortality [2–5] Therefore, controlling the dissemination of KPC-KP became a critical global issue. Interestingly, although the carbapenemase-encoding *bla*_KPC_-harboring plasmid has been detected in numerous *K. pneumoniae* sequence types (STs), the pandemic of KPC-KP is mainly associated with the clonal group 258 (CG258), which includes ST258, ST11, ST340, ST512, and others [2,6]. In China, the epidemic of KPC-KP is primarily restricted to ST11 *K. pneumoniae* [7]*.* Furthermore, *bla*_KPC_ is mostly located on the incompatibility group F (IncF) plasmids(Figure S2A) [2,7,8], although other plasmid scaffolds harbouring *bla*_KPC_-like genes (e.g. IncI2, IncX, IncA/C, IncR, and ColE1) have been identified among CG258 isolates [2,7,8]. The global dissemination of KPC-KP is strongly related to *K. pneumoniae* CG258 and *bla*_KPC_-IncF epidemic plasmids. Although the reasons behind this phenomenon are unclear, the ability of the CG258–*bla*_KPC_-IncF linkage to spread swiftly is beyond dispute.

Previous studies have shown that in addition to clonal dissemination, horizontal gene transfer (HGT) also contributes significantly to the pandemic dissemination of the *bla*_KPC_ gene [2,7,9]. The clustered regularly interspaced short palindromic repeats (CRISPRs) are part of the adaptive immune system in diverse bacteria and archaea, which can cleave foreign DNA in a programmable and sequence-specific manner, and are disadvantageous for HGT-driven gene expansion [10–12]. Thus, we proposed a hypothesis that high-risk CG258 lineage may lack or have lost such endogenous barriers (CRISPR) to HGT. Antibiotic use unintentionally selects for outgrowth of these immunocompromised strains with enhanced abilities to acquire *bla*_KPC_ genes, thereby assisting their rapid adaptation the hospital environment.

The new CRISPR-Cas classification of CRISPR-Cas includes two classes, five types and 16 subtypes[13]. By analyzing 203 whole genome sequences of *K. pneumoniae* in GenBank (Supplementary data 1a), only type I-E CRISPR systems were identified [14–16]. The mechanism for type I-E CRISPR-Cas genome defense has been recently reviewed [10,16]and is summarized as follows. CRISPR loci generally consists of short repeat sequences interspersed with unique spacer sequences that are homologous to sequences of invading DNA (“proto-spacers”) and a set of genes encoding nucleases (*cas* genes) are typically located near the CRISPR. Type I-E CRISPR systems possess eight *cas* genes (*cas1*, *cas2*, *cas3*, *cse1, cse2*, *cas7e, cas5e*, and *cas6e*) and either one or two CRISPR arrays [14]. On the basis of different layouts, type I-E CRISPR systems were further classified into two distinctive subtypes, type I-E and type I-E*[14]. Type I-E (in the cysH-iap region) is the canonical type I-E CRISPR-Cas system, while the other (Type I-E*, in ABC transport system-glyoxalase region) is variable, occasionally with a transposase-encoding gene integrated into the *cas* operon (Figure S1). Among the *cas* genes, *cse1*, *cse2*, *cas5e*, *cas6e* and *cas7e* encode proteins required for forming a CRISPR-associated complex for antiviral defense (Cascade) [13,14]. The Cascade–CRISPR RNA(crRNA) complex recognizes and binds to the foreign DNA, and then recruits the Cas3 protein for DNA degradation [13,17]. In addition, for foreign DNA, the matched proto-spacers (for targeted) and functional proto-spacer adjacent motifs (PAMs) (for distinguishing between self and foreign DNA) are necessary for CRISPR interference [18,19].

By analyzing the whole genome sequences of a series of *K. pneumoniae*, we have found 71 identified CRISPR loci of *K. pneumoniae*[16] (supplementary data1a) through the CRISPR finder[20]. Furthermore, bioinformatics analysis of 121 *bla*_KPC_-bearing plasmids (supplementary data 1b) also showed that the proto-spacer-positive plasmids were more commonly identified in the IncF group (Figure S2B), which possessed 14 proto-spacers (supplementary data 1c) matched for the CRISPR system in *K. pneumoniae*, than in the non-IncF group. Several matched proto-spacers identified from *bla*_KPC_-IncF plasmids suggest that these CRISPR-Cas systems may strongly associated with interfering with the survival of *bla*_KPC_-IncF plasmids. However, whether the existence of CRISPR in *K. pneumoniae* could impede the incursion and survival of such plasmids effectively remained unclear.

In this study, we explored the presence of type I-E CRISPR-Cas system in *K. pneumoniae,* especially focused on the CG258 lineage. We used the conjugation and plasmid stability assays to explore the function of CRISPR in perturbing the dissemination of the *bla*_KPC_-IncF plasmids. Furthermore, we also analyzed whether all *bla*_KPC_-IncF plasmids in this study would be good targets for such CRISPR systems. Hence, our goal was to further elucidate the function of CRISPR-Cas in anti- *bla*_KPC_-IncF-plasmid immunity and indicate possible associations between the scarcity of CRISPR-Cas system and globally successful dissemination of CG258 harbouring *bla*_KPC_ plasmids.

## Materials and Methods

### Bacterial strains

Four hundred and fifty-nine non-duplicated *K. pneumoniae* isolates were randomly isolated from individual patients at seven hospitals in six Chinese provinces, which represented different rates of carbapenem-resistance. (Figure S3), from January 2017 to February 2018. Among these, 247 carbapenem-resistant *K. pneumoniae* (CR-KP) and 212 carbapenem-sensitive *K. pneumoniae* (CS-KP) were collected contemporaneously from similar departments (Supplementary data 1d). The presence of *bla*_KPC_ gene about these strains was determined by the pair of primers listed in Table S1. Susceptibility testing was performed using the VITEK 2 system (bioMérieux, La Balme-les-Grottes, France) or using the broth microdilution method per the Clinical and Laboratory Standards Institute (CLSI) guidelines [21]. Multilocus sequence typing (MLST) was performed according to the protocol described on the Pasteur Institute MLST website for *K. pneumoniae*. Plasmid incompatibility type was identified by comparing with information in the Plasmid MLST locus/sequence definitions database (https://pubmlst.org/bigsdb?db=pubmlst_plasmid_seqdef). All strains and plasmids used in this study are listed in Table S1.

### Bioinformatics analysis

All *K. pneumoniae* complete genome sequences are publicly available (203 in total) and the sequences of the 121 *bla_KPC_*-positive plasmids were downloaded from the NCBI database in April 2018. CRISPR finder [20] was used with default parameters to identify the CRISPR loci in the genomes and determine the number and sequences of the spacers within CRISPR repeat arrays. Nucleotide BLAST was used to identify the Cas genes upstream and downstream of the CRISPR loci. Nucleotide BLAST was also used to search for matched protospacers with a minimum of 90% homology (29/32 nucleotides) on 121 *bla*_KPC_-positive plasmids. The proto-spacers and the PAMs located at the 5’end of proto-spacers on the plasmids were searched using Nucleotide BLAST. WebLogo [22] was used to analyze PAMs on *bla_KPC_*-IncF plasmids.

### Prevalence of CRISPR-Cas systems

The prevalence of *K. pneumoniae* isolates with CRISPR-Cas was determined using polymerase chain reaction (PCR) with the primers listed in Table S2. The presence of Type I-E CRISPR-Cas were preliminarily tested by amplifying the cysH-iap (Type I-E) and the ABC transport system-glyoxalase region (Type I-E*), as all of the CRISPR-Cas identified in the complete genome sequences of *K. pneumoniae* available in GenBank were located in cysH-iap or ABC transport system-glyoxalase region(Figure S1), and then further confirmed by amplifying the conserved gene cas1 and cas3, respectively.

### Construction of Escherichia coli BW25113 without and with the CRISPR-Cas system mutant strain

The JS681 (BW25113ΔCRISPR) strain, which delete complete original CRISPR-Cas systems in *E.coli* BW25113, was created also using the lambda RED recombination method as described previously[23]. The plasmid (pCRISPR-KP8) was assembled with three fragments: the two fragments (with 50-bp overlap) of CRISPR-Cas system were amplified from KP8 and the Pi-dependent plasmid backbone (Kan^R^) with one FRT site was amplified from pKD4 using primers with 30-bp homology to CRISPR-Cas fragment of KP8. Then, JS683 (BW25113-KP8CRISPR) was obtained by integrating the CRISPR-Cas system of KP8 (pCRISPR-KP8) into the JS681 strain via FLP-mediated site-specific recombination as described previously[24]. (Figure S4B)

### Construction of KP8 cas3 deletion mutants

The *cas3* deletion KP8 mutants were constructed using the lambda RED recombination method as described previously [23]. Primers designed to eliminate specific DNA stretches are listed in Table S2. When necessary, the resistance cassette introduced using the gene targeting procedure was eliminated via recombination with plasmid pCP20.

### Plasmid construction

The different proto-spacers and PAM sequences (along with same spacer) were synthesized by annealing single-stranded, complementary oligonucleotides and then cloned into a *Bsa I* site in the pUC-RP4 (Figure S4A). The RP4 Mob site increased the conjugation frequencies and amplified the variation among spacers as described previously[25]. All the primers and synthesized nucleotides used for cloning are listed in Table S2. All the plasmid fragments were combined using the in-fusion cloning method and the NEBuilder HiFi DNA assembly master mix (New England BioLabs).

### Conjugation assay

The donors and recipients were cultured to the logarithmic phase, mixed in 1:1 ratio, and then resuspended in 20 µl MgSO_4_ (10 mM). The resuspension was spotted on the Luria Bertani (LB) plate and incubated at 37°C overnight. Subsequently, the serial dilutions were plated in media with appropriate antibiotics. The conjugation frequency was calculated as the number of transconjugants per recipient.

### Analysis of plasmid stability

The strains transformed with the corresponding plasmids were grown overnight in LB broth with appropriate antibiotics. Next, 50 µl of the previous culture were inoculated in 5 ml fresh LB medium (containing 40 mM glucose) after every 12 h (37°C) for several passages[15,26]. For each culture, culture solution was serially diluted, spread on LB plates, and incubated at 37°C overnight and 100 colonies were collected from the LB plates. Single colonies were collected using tips and spotted on LB plates in the presence and absence of imipenem or chloramphenicol. The passages were discontinued only when the percentage of the resistant clones decreased to 50%. Then, the relative plasmid stability was calculated by comparing the number of colonies on the LB agar plate containing antibiotics with that on pure LB agar.

### Statistics

Statistical significance was assessed using a two-tailed Student’s t-test, one-way analysis of variance (ANOVA) test, or chi-square test of the GraphPad Prism8 software. *p* < 0.05 was considered statistically significant.

## Results

### Type I-E CRISPR systems are extremely rare in KPC-KP and CG258 lineage

**Figure 1.**
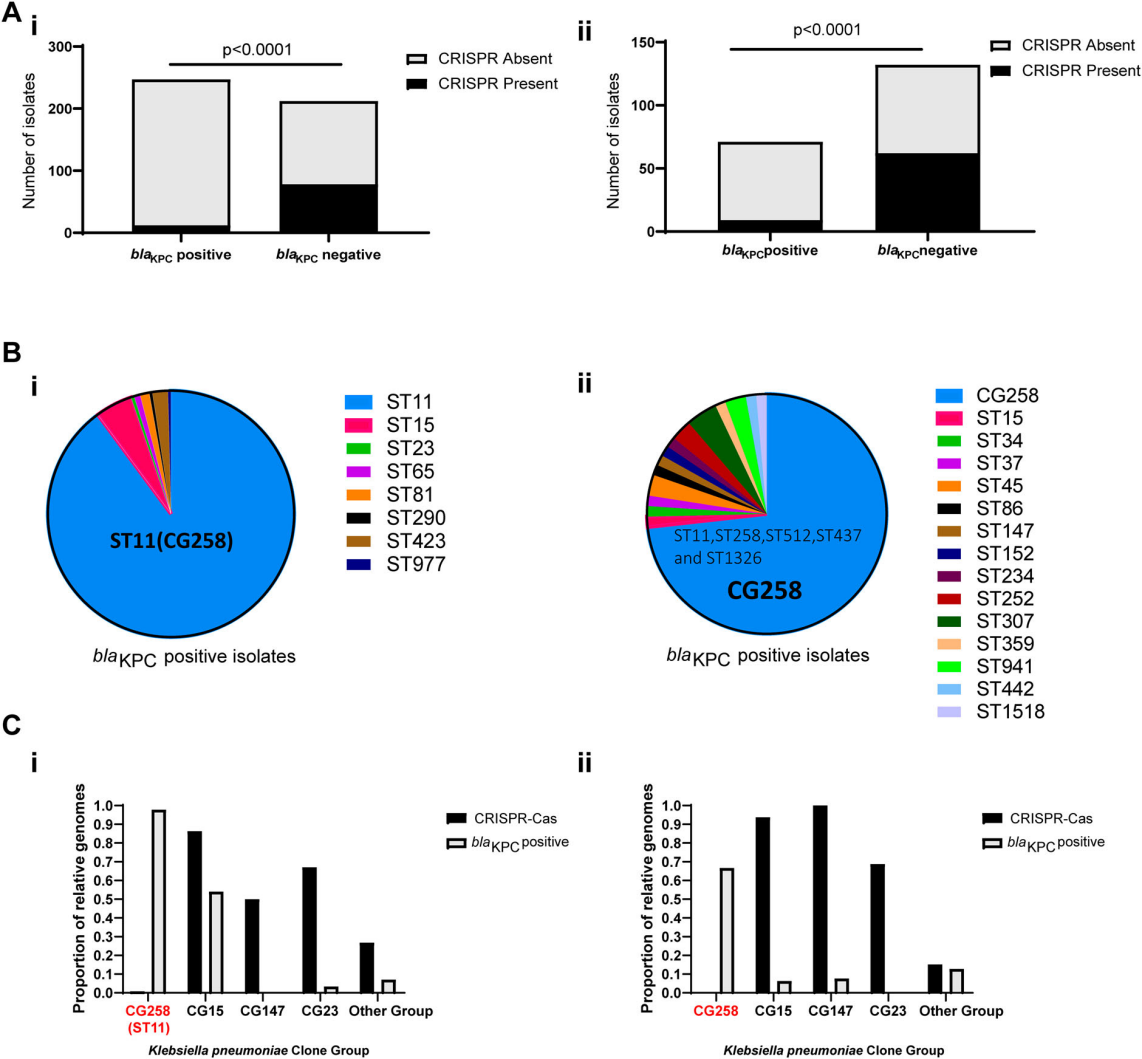
Presence of CRISPR-Cas system in *bla*_KPC_-positive /*bla*_KPC_-negative groups and **CG258/non-CG258 isolates.** (A) **i.** Presence of type I-E CRISPR systems in 459 Chinese clinical isolates collected in this study; **ii.** Presence of type I-E CRISPR systems in 203 completely sequenced strains available in GenBank. (B) **i.** MLSTs of *bla*_KPC_-positive group in459 Chinese clinical isolates; **ii.** MLSTs of *bla*_KPC_-positive group in 203 completely sequenced strains available in GenBank. (C) **i.** Presence of type I-E CRISPR systems among different clone groups in 459 Chinese clinical isolates collected in this study; **ii.** Presence of type I-E systems among different clone groups in 203 completely sequenced strains available in GenBank. *p < *0.0001 indicate significant differences between two groups as determined using Chi-square (and Fisher’s exact) test with Bonferroni correction of the GraphPad Prism8 software.

A total of 459 *K. pneumoniae* isolates was collected from six provinces in China. In parallel, 203 *K. pneumoniae* whole-genome sequences from worldwide and available over GenBank databases were analyzed to determine the distribution of the CRISPR-Cas loci in *K. pneumoniae*. We observed that the CRISPR-Cas loci were extremely rare in the KPC-positive isolates, whereas they were abundant among the KPC-negative isolates, either within the Chinese collection of clinical isolates (12/247 *vs.*78/212, *p* < 0.0001) or within the Genbank database (9/72 *vs.*62/131, *p* < 0.0001) (Figure 1A). These observations indicate a correlation between the scarcity of CRISPR-Cas system and *K. pneumoniae* with *bla*_KPC_.

Previous studies demonstrated that the pandemic of KPC-KP is mainly associated with the clonal group 258 (CG258) [2,6]. In this study, we also confirmed these features by analyzing 459 Chinese collection of clinical isolates and 203 global whole genome sequences (Figure 1B). The epidemic of *bla*_KPC_ is primarily restricted to CG258 *K. pneumoniae,* but the sequence types of KPC-negative strains were diverse (Supplementary data 1a and Supplementary data 1d). Thus, we speculate the CG258 lineage may be immunocompromised strains lacking type I-E CRISPR systems.

We investigated the co-distribution of CRISPR and acquired *bla*_KPC_ genes in different *K. pneumoniae* clone group, including high-risk CG258 group, MDR (Multidrug-Resistant) group (CG15 and CG147), hypervirulent group (CG23) and others. Consistent with our hypothesis, we observed that type I-E CRISPR systems were extremely rare in the CG258 lineages, either within the Chinese collection of clinical isolates or within the Global Genbank database (Figure 1C). Moreover, we found a significant inverse correlation between the presence of CRISPR and *bla_KPC_* genes in all *K. pneumoniae* clone groups. These findings indicated the lack of type I-E CRISPR systems in CG258 group may be related to the *bla*_KPC_ genes dissemination in such high-risk lineage.

### The CRISPR-Cas system in K. pneumoniae impedes IncF bla_KPC_-harboring plasmid conjugation

**Figure 2.**
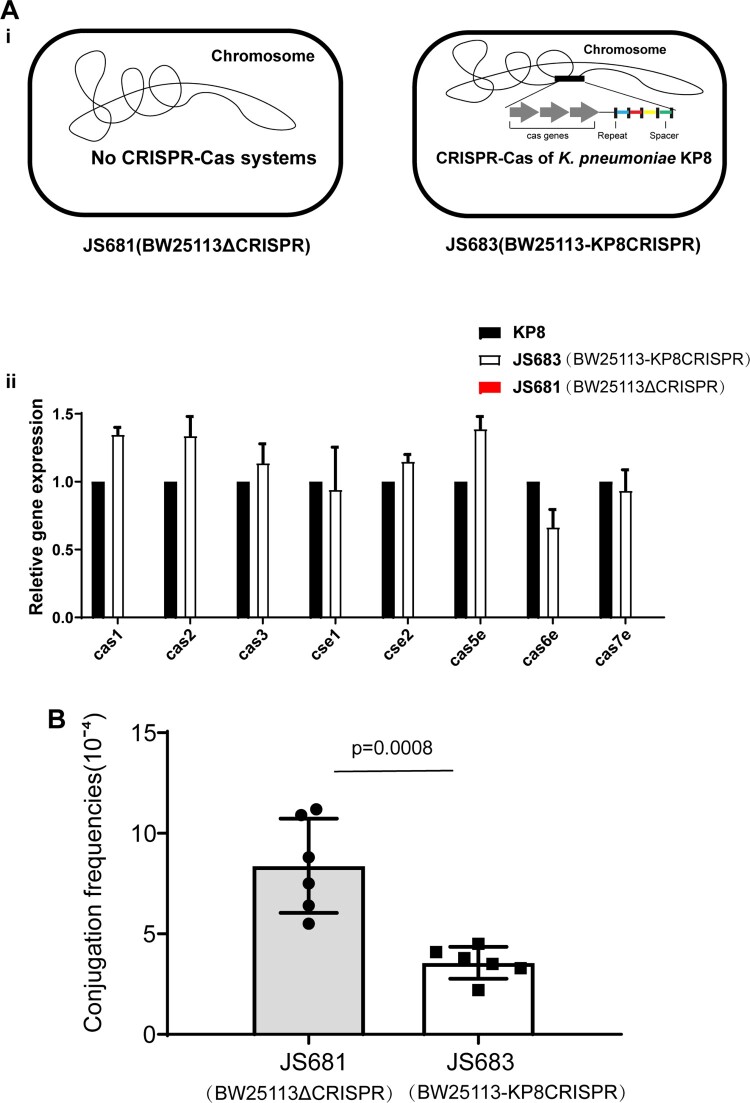
Conjugation frequencies of p187-2 in BW25113 strains with or without KP8 CRISPR. (A) (i) Schematic of JS681 and JS683. (ii)Expression of the Cas operon in the KP8, JS683 and JS681 cells. (B) Effect of the KP8 CRISPR on the conjugation frequencies of p187-2(IncF conjugative plasmid with *bla*_KPC_ and matched proto-spacers). The data represent the mean ± SD for six independent biological replicates. *p *= 0.0008 indicate significant differences between two groups as determined using two-tailed Student’s t-test.

The paucity of the CRISPR-Cas system in both CG258 *K. pneumoniae* and KPC-positive isolates implied that CRISPR-Cas may be involved in preventing the acquisition of the *bla*_KPC_-harboring plasmid. We used conjugation assays to determine whether the CRISPR-Cas system in *K. pneumoniae* is disadvantageous for the dissemination of *bla*_KPC-_ positive plasmids. The CRISPR-Cas system in the *K. pneumoniae* KP8 strain (type I-E, CP025636.1), being *bla*_KPC_-negative and belonging to ST458, was used as a model owing to its abundant matched spacers (Table S1). *Escherichia coli* BW25113 was used as recipient to generate recombinant strains with and without the CRISPR-Cas of strain KP8 (JS681 and JS683, Figure 2A). In addition, *E. coli* strain JS531 harbouring p187-2 (an IncF conjugative plasmid with *bla*_KPC_ and matched proto-spacers, CP025468.1) was used as donor (Table. S1).

Results of real-time PCR analysis confirmed that the KP8 Cas operon integrated in *E. coli* BW25113 was expressed successfully. Moreover, these results also proved the negative control JS681(BW25113ΔCRISPR) was useful since there was no Cas operon expression detected in such isolates (Figure 2B). Then, conjugation frequencies of the two *E. coli* recombinant strains were compared. As show in Figure 2C, the presence of the CRISPR-Cas of KP8 significantly decreased the conjugation frequencies in *E. coli* JS683 (BW25113-KP8CRISPR) cells by approximately 60%. This suggested that the type I-E CRISPR-Cas system in *K. pneumoniae* KP8 was involved in perturbing the acquisition of the IncF-*bla*_KPC_-harboring plasmids.

### The CRISPR-Cas system affects the stability of plasmids harbouring matched spacers

In addition to the plasmid acquisition process, stable plasmid persistence in a given host also plays a critical role in maintenance of antibiotic resistance. Therefore, a plasmid stability test was performed to investigate the survival of target plasmids harbouring matched spacers in both KP8 and JS683 (BW25113-KP8CRISPR) cells. In case a sequence on a plasmid may be targeted by the Cascade–crRNA complex, plasmid replication should be hindered. Therefore, when grown in a medium without antibiotics, plasmid abundance should be reduced, a phenomenon reflected by the lower percentage of resistant clones recovered. Moreover, the interference of the CRISPR-Cas system was conversely estimated by looking at specific antibiotic susceptibility recovery.

**Figure 3.**
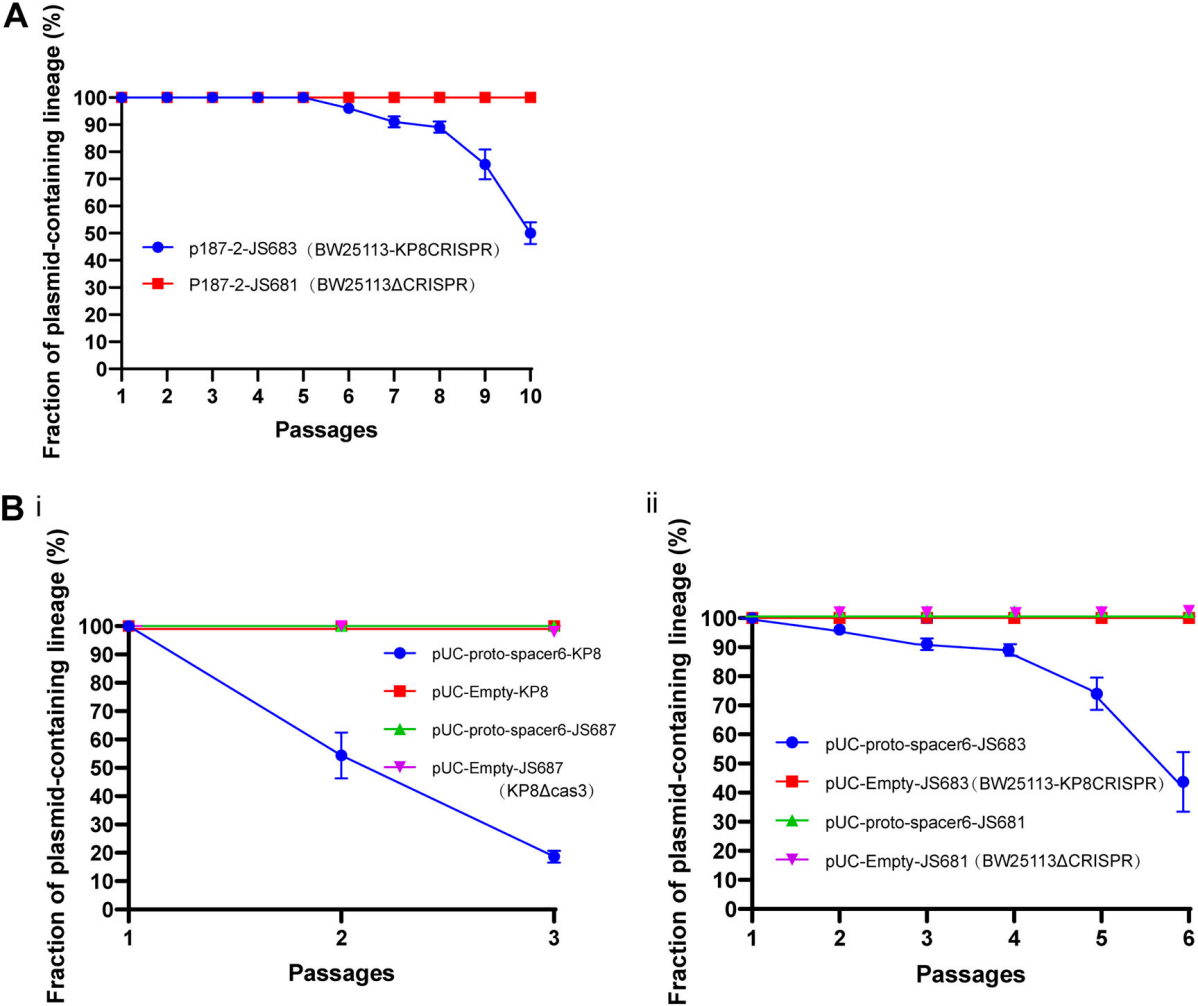
Plasmid stability in KP8 and JS683 (BW25113-KP8CRISPR) cells. (A) The *E. coli* strains JS683 and JS681 were transformed with a clinical plasmid p187-2 (an IncF conjugative plasmid with *bla*_KPC_ and matched spacers). Plasmid stability experiment results during10 passages. The number of imipenem-resistant clones was lesser in the CRISPR-positive strain (blue line) at the 10th passage than in the CRISPR-negative strain (red line), in which the number of resistant clones were no altered. (B) Two chloramphenicol-resistant plasmids with or without matched spacer were transformed into KP8, JS683, and their CRISPR-mutant versions(JS687, KP8ΔCas3 and JS681,BW25113ΔCRISPR). Plasmid stability experiment results during 3 passages(i) or 6 passages(ii). The elimination rates of pUC-proto-spacer 6 in the CRISPR-positive strain (blue lines) decreased to variable extents, whereas that of pUC-empty in all strains were identical. The stability of all plasmids was identical in the CRISPR deletion strain(JS687, KP8ΔCas3 and JS681,BW25113ΔCRISPR). All experiments were conducted in triplicate.

To investigate whether the CRISPR-Cas system of strain KP8 inhibited the presence of the *bla*_KPC_-IncF plasmids, the natural plasmid p187-2 was transformed into both *E. coli* JS683 (BW25113-KP8CRISPR) and *E. coli* JS681 (BW25113ΔCRISPR) strains and replication interference was assessed. It showed that the percentage of resistant clones in the CRISPR-positive strain ranged from 46% to 54% after 10 passages in LB medium, whereas the CRISPR-negative strain still retained the resistance plasmid after the same number of passages (Figure 3A). In agreement with the results of the plasmid stability assay, minimum inhibitory concentrations (MICs) of imipenem for these clones further confirmed that the CRISPR-Cas system of KP8 significantly and negatively contributed to restrain the retention of IncF-*bla*_KPC_ plasmids (Table. S3).

As host differences may affect the CRISPR interference, two plasmids (chloramphenicol-resistant) were constructed with or without matched proto-spacers to further evaluate how CRISPR operate in the original *K. pneumoniae* strain (KP8). Furthermore, the KP8 Cas3 deletion mutant was created as the negative control. Both plasmids were transformed into *K. pneumoniae* KP8 and KP8 mutants and into *E. coli* BW25113 with or without CRISPR. This experiment showed that the maintenance ability of matched plasmids in the CRISPR-positive strains, especially in the *K. pneumoniae* KP8, decreased significantly compared to the negative strains and non-matched plasmid (Figure 3B). Those observations were in full agreement with obtained MIC values (Table. S3). Overall, those findings confirmed that this difference in term of plasmid stability was unequivocally related to the CRISPR-Cas system-mediated interference.

### Proto-spacers located on bla_KPC_-IncF plasmids can be well-interfered by the CRISPR system

The interference between the CRISPR system and the occurrence of the IncF*-bla*_KPC_ plasmids in *K. pneumoniae* was investigated. Our aim was to evaluate whether all the IncF *bla*_KPC_-harboring plasmids in this study were effectively targeted by CRISPR. Previous studies have shown that the matched proto-spacers on plasmids are the vital clues for CRISPR scanning and cleavage [12]. Therefore, degradation of plasmids depends on its proto-spacers.

**Figure 4.**
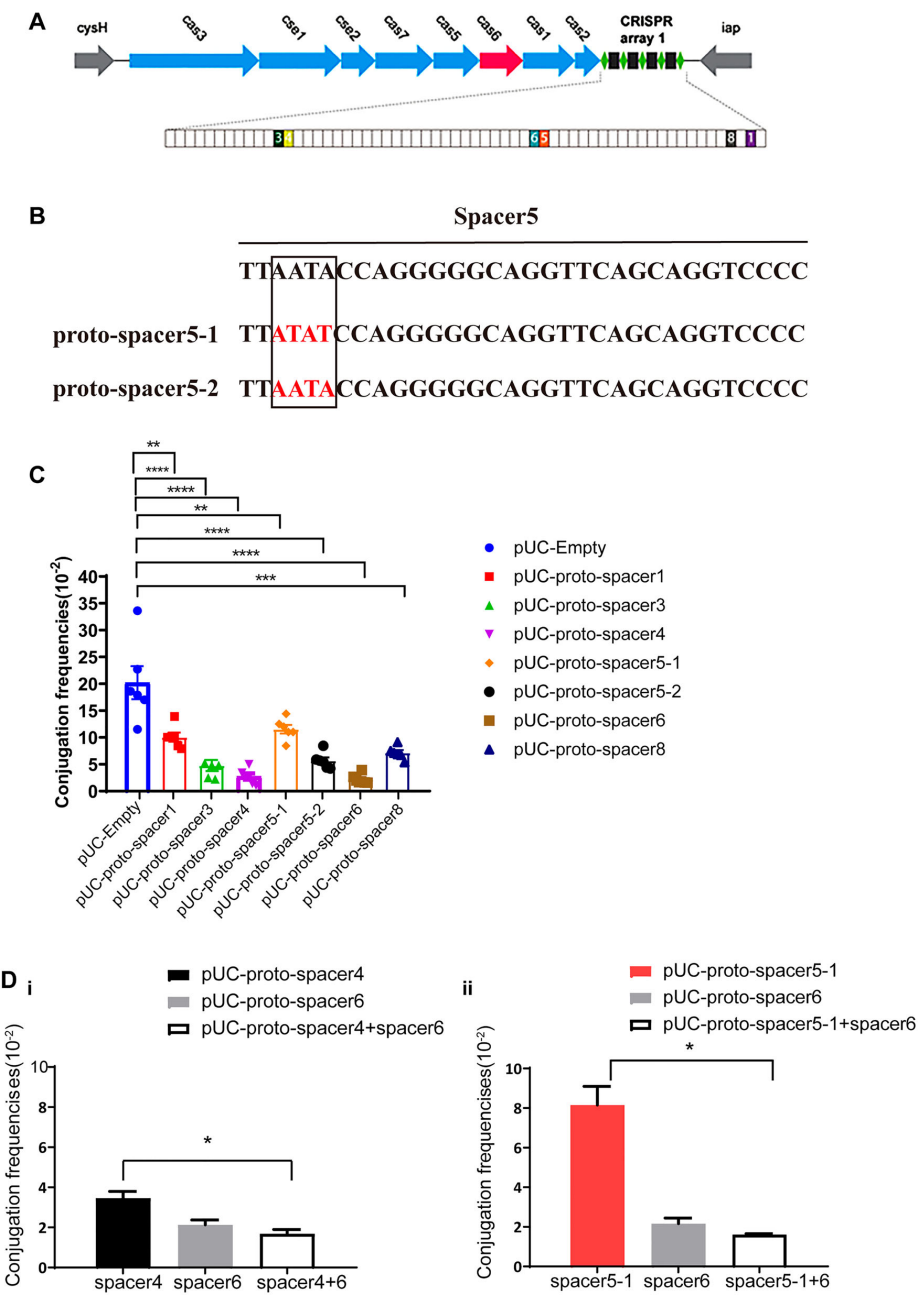
Characteristics of proto-spacers located on the *bla*_KPC_ -IncF plasmids. (A) Schematic showing the CRISPR-Cas system in KP8. Genes are depicted as arrows in different colours and the IncF plasmid-matched spacers are shown as colourful boxes. (B) Two subtypes of proto-spacer 5, in which the nucleotide sequence in red represents the base mutation. (C) Conjugation frequencies of different proto-spacers. (D) Conjugation frequencies of single and combined proto-spacers. The results of the conjugation assay are presented as means ± SD from six independent experiments. * *p* < 0.05, ** *p* < 0.01, *** *p* < 0.001, ****p < 0.0001 indicate significant differences between the strains and the control group (pUC-Empty) as determined using one-way ANOVA with Dunnett correction(C). Statistical significance between the two strains was assessed using a two-tailed Student’s t-test with Bonferroni correction of the GraphPad Prism8 software. * *p* < 0.05 was considered statistically significant (D).

*We have previously observed that almost all the* bla*_KPC_-IncF plasmids* contain more than one type of proto-spacers that matched with the CRISPR array in *K. pneumoniae* (Supplementary data 1b). To investigate whether all types of proto-spacers present onto IncF plasmids can be targeted, the KP8 CRISPR was used as a model. Six types of proto-spacers (Table S4) that matched the KP8 CRISPR were used, of which proto-spacer 5 had two subtypes (Figure 4).

Conjugation is a stable assay for assessing CRISPR-Cas activity. Hence, we used this assay to monitor how different types of sequences targeted by the crRNA affect plasmid replication. Seven plasmids harbouring variable proto-spacers were constructed and transformed into *E. coli* S17-1 λpir (RP4+) that was subsequently used as donor (Figure S4A) while *E. coli* JS683(BW25113-KP8CRISPR) was used as recipient for conjugative assays. Compared to the negative control (pUC-Empty), the frequencies of all seven plasmids decreased significantly, among which the frequency of proto-spacer 6 was reduced the most and that of the proto-spacer 5–1 was reduced the least (90% *vs.* 42.2%) (Figure 4C). Notably, frequencies of the well-paired proto-spacer 5–2 decreased by 51% compared to that of the proto-spacer 5–1 (containing 3 bp substitutions), although both belonged to the same type. These results indicated that all matched sequences can be targeted, albeit with variations in the CRISPR immune response.

The aforementioned results showed that not all plasmid-borne proto-spacers may be interfered in a similar manner, suggesting that CRISPR tend to leave multiple matched proto-spacers on one plasmid possibly because that may promote effective plasmid elimination. To account for this possibility, two recombinant plasmids were constructed: harbouring either plasmids containing two highly functional proto-spacers (proto-spacer 4 and proto-spacer 6) or containing the supposed strongest and weakest- functional proto-spacers (proto-spacer 5–1 and proto-spacer 6), respectively. Compared to the plasmids harbouring single proto-spacer4 or proto-spacer 5-1, the conjugation frequencies of the combined plasmids all decreased and were similar to the frequencies of proto-spacer 6 (Figure 4D). These findings indicated that the occurrence of high-active proto-spacer 6 made up for the low-active proto-spacers, resulting a more effective plasmid elimination.

Taken together, these results demonstrated that although the CRISPR system may specifically interfere with certain spacers effectively, multiple proto-spacers on a single plasmid may compensate for this bias and accelerate its elimination.

### PAMs harboured by the bla_KPC_-IncF plasmids are conserved and functional

**Figure 5.**
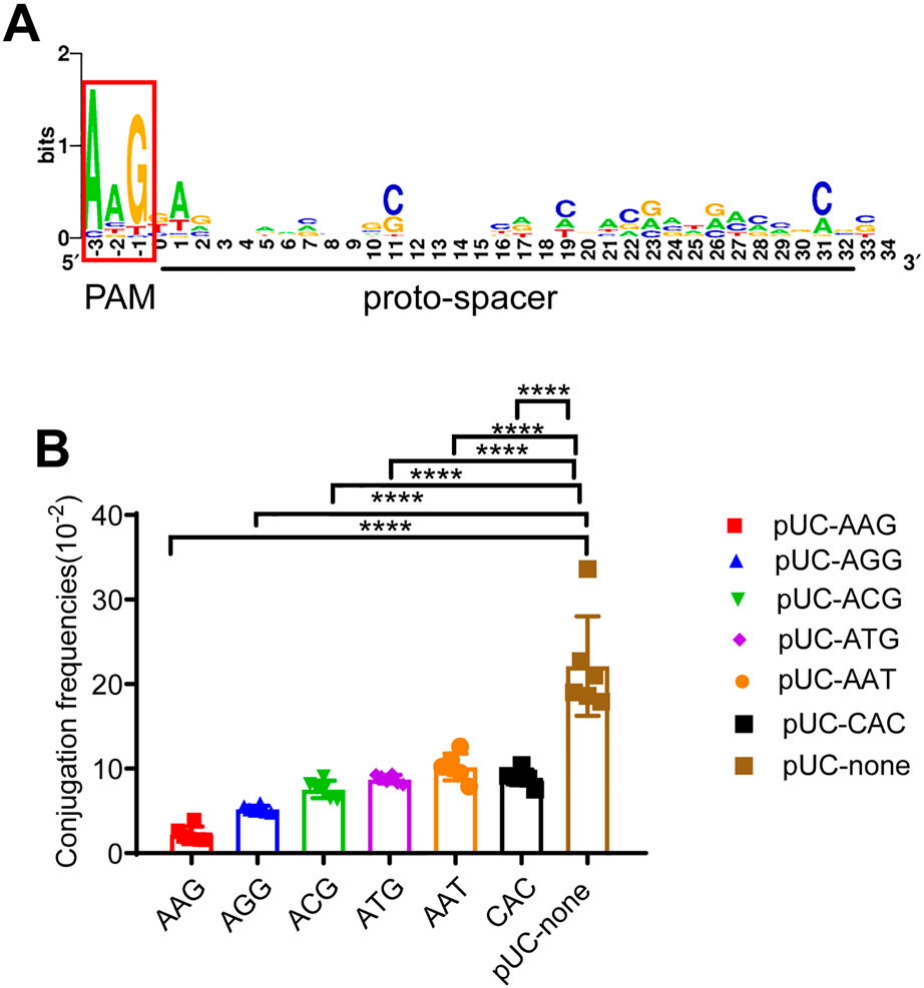
Characteristics of PAMs adjunct to the proto-spacers harboured by *bla*_KPC_-IncF plasmids. (A) WebLogo was used to analyze PAMs from *bla*_KPC_-IncF plasmids. The first nucleotide of the proto-spacer is at position 0. WebLog of the proto-spacer, as well as those 3 nucleotides upstream and downstream of the proto-spacer are shown. The relative letter size indicates the base frequency at that position. (B) Conjugation frequencies of proto-spacers with different PAMs. The data represent the mean ± SD for six independent biological replicates. * *p* < 0.05, ** *p* < 0.01, *** *p* < 0.001, ****p < 0.0001 indicate significant differences between the strains and the control group (pUC-none) as determined using one-way ANOVA with Dunnett correction.

In addition to the matched pro-spacers, functional PAM is also essential for the Cascade–crRNA complex to target the plasmid. To investigate whether PAMs on IncF- *bla*_KPC_ plasmids were conserved respect to the CRISPR system in *K. pneumoniae,* all the target proto-spacers of 54 IncF *bla*_KPC_-positive plasmids were analyzed (Supplementary data 1b). As shown in Figure 5A, there was a strong bias for the AAG motif, which confirmed the existence of CRISPR-specific PAMs.

As the AAG motif was the most abundant PAM (56.7%, 389/687) (Figure 5A), we speculated that it was also subject to the strongest CRISPR-interference. To verify this hypothesis, six plasmids were constructed containing different PAMs (AAG, AGG, ACG, ATG, AAT, and CAC) respectively (Figure S4A) originating from the IncF plasmids and conjugation assay were performed to assess their function. In agreement with our assumption, the frequency of degradation of the AAG-PAM (90.3%) was higher among the 6 PAMs (Figure.5B) compared to those of plasmids with no PAM (pUC-none). These observations further demonstrate that the functions of different PAMs are consistent with the PAM preferences of the CRISPR cascade.

## Discussion

The widespread *K. pneumoniae* CG258 isolates are important human pathogens known to cause urinary tract infections, respiratory tract infections, and bloodstream infections, which have spread extensively throughout the world [2,6]. Several studies have demonstrated that the CG258 strains and related *bla*_KPC_-IncF plasmids are responsible for the sudden increase in the population of multidrug resistance among *K. pneumoniae* isolates [8,27]. However, little is known about the exact factors of CG258-*bla*_KPC_ successful dissemination. Our results indicated the type I-E CRISPR-Cas systems impacted the spread of *bla*_KPC_-plasmids in *K. pneumonia*. The scarcity of type I-E CRISPR-Cas systems in CG258 lineage, allowing them to readily acquire and adapt to *bla*_KPC_-plasmids.

Type I-E CRISPR-Cas systems identified in *K. pneumoniae* are categorized into subtypes type I-E and type I-E*[14], and the anti-plasmid immunity in the type I-E*-CRISPR-positive *K. pneumoniae* NTUH-K2044 strain (ST23) have been well-described [15]. However, their relationship with *bla*_KPC_ plasmids was not understood. Hence, in this study, we investigated the type I-E CRISPR prevalence in *K. pneumoniae* at present and observed dearth of drug-resistant *K. pneumoniae* CG258 strains. Furthermore, the proto-spacers harboured by the IncF-*bla*_KPC_ plasmids, matched for CRISPR, were collected and analyzed. Results indicated that more than one matched sequence were present in all these plasmids (Supplementary data 1c). As the role of type I-E*-CRISPR activity in anti-plasmid immunity has already been confirmed [15], the type I-E CRISPR in the KP8 strain was selected as a model for further study.

Conjugation and plasmid stability assays were used to comprehensively assess the ability of CRISPR to impede the transmission of IncF-*bla*_KPC_ plasmids. The results demonstrated that the CRISPR harboured by *K. pneumoniae* resulted in effective immunity to drug-resistance plasmids containing matched proto-spacers. The results of the plasmid stability assay also revealed that complete plasmid elimination required cumulative CRISPR interference, consistent with the results of former studies [15]. Interestingly, although the Cas operon transcript of an alternative isolate (JS683,*E. coli* BW25513-KP8 CRISPR) and original strain (*K. pneumoniae* KP8) were similar, according to the results of the plasmid stability assay the CRISPR system in KP8 cells was more competent than that in JS683(*E.coli* BW25513-KP8 CRISPR), which may be ascribed to differences in host regulators in these two organisms. In addition, this can explain the absence of a sharp decrease in the conjugation frequency of the substitute strains (*E. coli*) and the more significant decrease in the frequency of the KP8(*K. pneumoniae*) isolate could be speculated.

Previous studies have demonstrated that the IncF plasmids are the most predominant *bla*_KPC_-containing plasmid types [2,7]. In this study, we observed that the IncF-*bla*_KPC_ plasmids were also favoured by the proto-spacers in the 121 *bla*_KPC_ plasmids we studied (Supplementary data 1b and Figure S2B). To clarify whether the CRISPR system can effectively prevent *bla*_KPC_ dissemination, we should further validate whether all the proto-spacers and PAMs harboured by *bla*_KPC_-IncF-plasmids are functionally recognized. Our results elucidated that all matched sequences can be targeted, although not all can be flawlessly interfered with. Many studies suggest that the base pairing at the −1 position and the seed region (nucleotides +1 to +5, +7, and +8) are critical for target recognition by Cascade [28], which is in agreement with the observation that proto-spacer 5–2 generated more effective immune response than proto-spacer 5–1 (Figure 4). In addition to the well base-paired, the positions at which spacers are inserted in the CRISPR array and spacer GC content (62.5% was optimal), are also essential for CRISPR interference activity [29]. Among these selected proto-spacers, the CRISPR-interference increased as the GC content in the spacer until reached 62.5% [29], and the more proximal a spacer is to the leader sequence, the more efficiently can it be transcribed [10,30]. Therefore, although each matched sequence was targeted, not all proto-spacers could trigger interference with equal efficiency. These observations explained the presence of multiple spacers in the plasmids, which were required to compensate for the presence of low-active mismatch-containing spacers [10]. Our observations also support the view that multiple proto-spacers on plasmids promote the efficiency of CRISPR-mediated degradation. In summary, almost all the *bla*_KPC_ -IncF plasmids can be well-targeted as they harbour manifold proto-spacers.

Interestingly, both type I-E CRISPR-Cas systems identified in *E. coli* [31] and *K. pneumoniae* are prone to targeting the AAG PAM, and PAMs located on IncF plasmids predominantly contain the AAG motif (Figure 5A). In such CRISPR systems, the PAM selectivity of the CRISPR adaptation machinery has co-evolved with the CRISPR interference machinery; thus, both the structural basis of *cas1-cas2* (adaption) and *cse1* (interference) are responsible for PAM determination, which specifically recognize the AAG as the consensus PAM sequence [10,32]. In this study, we demonstrated that the AAG PAM was conserved in *bla*_KPC_-IncF plasmids and that the AAG motif showed the strongest CRISPR interference. As previous studies have demonstrated that only the proto-spacers were flanked by a functional PAM, they can be effectively recognized by the cascade [33]. Our observations regarding PAMs further verified that the *bla*_KPC_-IncF plasmids are good targets for CRISPR.

Although the CRISPR could influence the obtention of *bla*_KPC_-IncF plasmids in *K. pneumoniae*, the type I-E CRISPR systems were not flawless barriers to plasmid transfer. We could found other *K. pneumoniae* lineages such as CG 65, CG86 and CG40, also seem to lack type I-E CRISPR-Cas[33], but they were not favoured by *bla*_KPC_-IncF plasmids, that may be caused by polysaccharide capsule (CG 65 and CG86, hypervirulent clones) or harbouring R-M (Restriction-Modification) systems (the other defense system)[34]. Notably, in addition to the high-risk CG258 lineage, other clone groups including CG15 and CG147 also be considered to be important causes of carbapenem-resistant infections[35]. However, these MDR groups could acquire several resistance genes such as *bla*_VIM_, *bla*_IMP_ and *bla*_OXA-48_[35], successfully evaded restriction by CRISPR systems. Moreover, previous studies also found CRISPR systems could not influence the invasion of all antibiotic resistance genes into *K. pneumoniae* [36]. These defense failures may be attributed to the no-matched spacers of invading resistance genes or involved with CRISPR tolerance[11].

Remarkably, unlike the inverse relationship we found between *bla*_KPC_ genes and CRISPR, the coexistence of a CRISPR array and multiple copies of the same β-lactamase genes in the chromosome were found in several *K. pneumoniae* isolates [37]. These novel findings proposed a potential mechanism that CRISPR could promote the antimicrobial resistance gene mobilization from plasmids into the chromosome, through degradation the targeting plasmids[37]. As well as the CRISPR systems, the other immune systems (R-M systems) carried by the bacteria also play an important role in regulating the plasmid dissemination. Previous study in *E. faecalis* indicated that CRISPR-Cas defense and R-M defense individually contribute significantly to anti-plasmid genome defense[38]. Over all, these observations all verified that the CRISPR-Cas system was not the only limiting factor for controlling plasmid attack, as other differences between the resistance-plasmids and several host-specific variations may also affect plasmid intrusion, which will be the focus of future work.

In addition to exploring the relationship between *bla*_KPC_ and CRISPR in *K. pneumoniae*, we also found the hypervirulent group, especially the CG23 lineage, was preferred by the type I-E CRISPR, in keeping with the results of former studies [36]. These interesting discoveries were also worth further study.

In conclusion, our work demonstrates the scarcity of type I-E CRISPR-Cas systems is a probable factor leading to *bla*_KPC_-IncF plasmids can be propagated in *K. pneumoniae* CG258 lineage. Meanwhile, antibiotic use inadvertently selects for these strains with *bla*_KPC_-plasmids, which provide a potential explanation for the CG258 epidemic success.

## Supplementary Material

Supplemental Material

## Abbreviations

ABBREVIATIONS: CG258: Clonal group 258; CRISPR: Clustered regularly interspaced short palindromic repeats; IncF: Incompatibility group F; KPC: *Klebsiella pneumoniae* carbapenemase; KPC-KP: KPC-producing *K. pneumoniae;* LB: Luria Bertani; MICs: Minimum inhibitory concentrations; PCR: Polymerase chain reaction; PAMs: Proto-spacer adjacent motifs; ST: Sequence type
