# Supplementary material for: The type I-E CRISPR-Cas system influences the acquisition of blaKPC-IncF plasmid in Klebsiella pneumonia
Source: Emerg Microbes Infect. 2020 May 20;9(1):1011–22. doi: 10.1080/22221751.2020.1763209 (PMC7301723; doi:10.1080/22221751.2020.1763209)
Supplement: Supplemental Material [file TEMI_A_1763209_SM1594.zip › Supplementary files/supplementary data1a.docx]

Supplementary data 1a. 203 completely sequenced *K. pneumoniae* strains used in this study.

* The ‘-’symbol indicates the absence of the CRISPR-Cas locus

On the basis of different layouts, the Type I-E CRISPR were furtherly classified and designated as Type I-E and Type I-E*

#The ‘-’symbol indicates the absence of the plasmid carrying *bla*_KPC_

**Several strains have the same layout of the CRISPR array

| **203 completely sequenced K. pneumoniae strains used in this study** | | | | | | | | | |  | No. of spacer matches in *bla*_KPC_-plasmid | | | | | | | | | | | | | | |
| --- | --- | --- | --- | --- | --- | --- | --- | --- | --- | --- | --- | --- | --- | --- | --- | --- | --- | --- | --- | --- | --- | --- | --- | --- | --- |
| Number | CRISPR  -Cas* | CRISPR array | Strain | Clonal complex | MLST | Collection location and date | GenBank accession no. of the chromosome | *bla* KPC# | Typing | No. of distinct  spacer matches | Total | Spacer1 | Spacer2 | Spacer3 | Spacer4 | Spacer5 | Spacer6 | Spacer7 | Spacer8 | Spacer9 | Spacer1 0 | Spacer1 1 | Spacer1 2 | Spacer1 3 | Spacer1 4 |
| 1 | I-E | HZW25 | HZW25 | Non-CC258 | 34 | USA(2016) | CP025211.1 | - |  |  |  |  |  |  |  |  |  |  |  |  |  |  |  |  |  |
| 2 | I-E | KPNIH27 | KPNIH27 | Non-CC258 | 34 | USA (2012) | CP007731.1 | CP007732.1 | IncA/C | 0 | 0 | 0 | 0 | 0 | 0 | 0 | 0 | 0 | 0 | 0 | 0 | 0 | 0 | 0 | 0 |
| 3 | I-E | CAV1016** | AR_0126 | Non-CC258 | 45 | USA(2016) | CP021740.1 | CP021743.1 | HCM1 259 | 0 | 0 | 0 | 0 | 0 | 0 | 0 | 0 | 0 | 0 | 0 | 0 | 0 | 0 | 0 | 0 |
| 4 | I-E | CAV1016 | CAV1016 | Non-CC258 | 45 | USA (2007) | CP017934.1 | CP017937.1 | HCM1_259 | 0 | 0 | 0 | 0 | 0 | 0 | 0 | 0 | 0 | 0 | 0 | 0 | 0 | 0 | 0 | 0 |
| 5 | I-E | QS17-0161 | QS17-0161 | Non-CC258 | 45 | USA(2016) | CP024458.1 | - |  |  |  |  |  |  |  |  |  |  |  |  |  |  |  |  |  |
| 6 | I-E | Kp52.145 | Kp52.145 | Non-CC258 | 66 | France (2015) | FO834906.1 | - |  |  |  |  |  |  |  |  |  |  |  |  |  |  |  |  |  |
| 7 | I-E | SB3432 | SB3432 | Non-CC258 | 67 | USA(2016) | FO203501.1 | - |  |  |  |  |  |  |  |  |  |  |  |  |  |  |  |  |  |
| 8 | I-E | 002SK2 | 002SK2 | Non-CC258 | 147 | USA(2016) | CP025515.1 | - |  |  |  |  |  |  |  |  |  |  |  |  |  |  |  |  |  |
| 9 | I-E | 825795-1** | 825795-1 | Non-CC258 | 147 | USA(2016) | CP017985.1 | - |  |  |  |  |  |  |  |  |  |  |  |  |  |  |  |  |  |
| 10 | I-E | 825795-1 | Kp_Goe_822579 | Non-CC258 | 147 | USA(2016) | CP018140.1 | - |  |  |  |  |  |  |  |  |  |  |  |  |  |  |  |  |  |
| 11 | I-E | 825795-1 | Kp_Goe_149473 | Non-CC258 | 147 | USA(2016) | CP018686.1 | - |  |  |  |  |  |  |  |  |  |  |  |  |  |  |  |  |  |
| 12 | I-E | 825795-1 | Kp_Goe_149832 | Non-CC258 | 147 | USA(2016) | CP018695.1 | - |  |  |  |  |  |  |  |  |  |  |  |  |  |  |  |  |  |
| 13 | I-E | 825795-1 | Kp_Goe_827024 | Non-CC258 | 147 | USA(2016) | CP018701.1 | - |  |  |  |  |  |  |  |  |  |  |  |  |  |  |  |  |  |
| 14 | I-E | 825795-1 | Kp_Goe_827026 | Non-CC258 | 147 | USA(2016) | CP018707.1 | - |  |  |  |  |  |  |  |  |  |  |  |  |  |  |  |  |  |
| 15 | I-E | 825795-1 | Kp_Goe_152021 | Non-CC258 | 147 | USA(2016) | CP018713.1 | - |  |  |  |  |  |  |  |  |  |  |  |  |  |  |  |  |  |
| 16 | I-E | 825795-1 | KP_Goe_828304 | Non-CC258 | 147 | USA(2016) | CP018719.1 | - |  |  |  |  |  |  |  |  |  |  |  |  |  |  |  |  |  |
| 17 | I-E | MS6671** | FDAARGOS_44 | Non-CC258 | 147 | USA(2016) | CP023925.1 | CP023928.1 | IncFIIK | 8 | 8 | 1 | 1 | 0 | 0 | 0 | 1 | 1 | 0 | 1 | 1 | 1 | 0 | 1 | 0 |
| 18 | I-E | MS6671 | MS6671 | Non-CC258 | 147 | Australia(2015) | LN824133.1 | - |  |  |  |  |  |  |  |  |  |  |  |  |  |  |  |  |  |
| 19 | I-E | MS6671 | KP5 | Non-CC258 | 147 | Singapore(2015) | CP012426.1 | - |  |  |  |  |  |  |  |  |  |  |  |  |  |  |  |  |  |
| 20 | I-E | MS6671 | TGH13 | Non-CC258 | 147 | Belgium(2015) | CP012745.1 | - |  |  |  |  |  |  |  |  |  |  |  |  |  |  |  |  |  |
| 21 | I-E | MS6671 | AATZP | Non-CC258 | 147 | USA(2016) | CP014755.1 | - |  |  |  |  |  |  |  |  |  |  |  |  |  |  |  |  |  |
| 22 | I-E | MS6671 | SKGH01 | Non-CC258 | 147 | USA(2016) | CP015500.1 | - |  |  |  |  |  |  |  |  |  |  |  |  |  |  |  |  |  |
| 23 | I-E | MS6671 | AR_0138 | Non-CC258 | 147 | USA(2016) | CP021757.1 | - |  |  |  |  |  |  |  |  |  |  |  |  |  |  |  |  |  |
| 24 | I-E | MS6671 | AR_0145 | Non-CC258 | 147 | USA(2016) | CP021939.1 | - |  |  |  |  |  |  |  |  |  |  |  |  |  |  |  |  |  |
| 25 | I-E | MS6671 | AR_0152 | Non-CC258 | 147 | USA(2016) | CP021944.1 | - |  |  |  |  |  |  |  |  |  |  |  |  |  |  |  |  |  |
| 26 | I-E | MS6671 | FDAARGOS_43 | Non-CC258 | 147 | USA(2016) | CP023913.1 | - |  |  |  |  |  |  |  |  |  |  |  |  |  |  |  |  |  |
| 27 | I-E | MS6671 | DA48896 | Non-CC258 | 147 | USA(2016) | CP024429.1 | - |  |  |  |  |  |  |  |  |  |  |  |  |  |  |  |  |  |
| 28 | I-E | MS6671 | CRKP-2297 | Non-CC258 | 147 | USA(2016) | CP024834.1 | - |  |  |  |  |  |  |  |  |  |  |  |  |  |  |  |  |  |
| 29 | I-E | MS6671 | CRKP-1215 | Non-CC258 | 147 | USA(2016) | CP024838.1 | - |  |  |  |  |  |  |  |  |  |  |  |  |  |  |  |  |  |
| 30 | I-E | AR_0158** | AR_0158 | Non-CC258 | 163 | USA(2016) | CP021696.1 | - |  |  |  |  |  |  |  |  |  |  |  |  |  |  |  |  |  |
| 31 | I-E | MS6671 | Kpn223 | Non-CC258 | 273 | USA(2016) | CP015025.1 | - |  |  |  |  |  |  |  |  |  |  |  |  |  |  |  |  |  |
| 32 | I-E | WCHKP34 | WCHKP34 | Non-CC258 | 273 | - | CP025963.1 | - |  |  |  |  |  |  |  |  |  |  |  |  |  |  |  |  |  |
| 33 | I-E | TGH8** | TGH8 | Non-CC258 | 383 | USA(2016) | CP012743.1 | - |  |  |  |  |  |  |  |  |  |  |  |  |  |  |  |  |  |
| 34 | I-E | TGH8 | TGH10 | Non-CC258 | 383 | USA(2016) | CP012744.1 | - |  |  |  |  |  |  |  |  |  |  |  |  |  |  |  |  |  |
| 35 | I-E | KPNIH31** | KPNIH31 | Non-CC258 | 392 | USA (2013) | CP009876.1 | in chromosome | |  |  |  |  |  |  |  |  |  |  |  |  |  |  |  |  |
| 36 | I-E | KPNIH31 | CN1 | Non-CC258 | 392 | USA(2016) | CP015382.1 | - |  |  |  |  |  |  |  |  |  |  |  |  |  |  |  |  |  |
| 37 | I-E | GN-2 | GN-2 | Non-CC258 | 485 | USA(2016) | CP019160.1 | - |  |  |  |  |  |  |  |  |  |  |  |  |  |  |  |  |  |
| 38 | I-E | YH43 | YH43 | Non-CC258 | 906 | Japan | CP020847.1 | - |  |  |  |  |  |  |  |  |  |  |  |  |  |  |  |  |  |
| 39 | I-E | CAV1344** | CAV1193 | Non-CC258 | 941 | USA (2010) | CP013322.1 | CP013325.1 | HCM1 259 | 0 | 0 | 0 | 0 | 0 | 0 | 0 | 0 | 0 | 0 | 0 | 0 | 0 | 0 | 0 | 0 |
| 40 | I-E | CAV1344 | CAV1344 | Non-CC258 | 941 | USA (2010) | CP011624.1 | CP011622.1 | IncA/C | 0 | 0 | 0 | 0 | 0 | 0 | 0 | 0 | 0 | 0 | 0 | 0 | 0 | 0 | 0 | 0 |
| 41 | I-E* | FDAARGOS_15 | FDAARGOS_15 | Non-CC258 | 13 | USA(2016) | CP014123.1 | - |  |  |  |  |  |  |  |  |  |  |  |  |  |  |  |  |  |
| 42 | I-E* | PittNDM01** | PittNDM01 | Non-CC258 | 14 | USA(2016) | CP006798.1 | - |  |  |  |  |  |  |  |  |  |  |  |  |  |  |  |  |  |
| 43 | I-E* | PittNDM01 | KP617 | Non-CC258 | 14 | USA(2016) | CP012753.1 | - |  |  |  |  |  |  |  |  |  |  |  |  |  |  |  |  |  |
| 44 | I-E* | PittNDM01 | U25 | Non-CC258 | 14 | USA(2016) | CP012043.1 | - |  |  |  |  |  |  |  |  |  |  |  |  |  |  |  |  |  |
| 45 | I-E* | PittNDM01 | K.pneumoniae 11 | Non-CC258 | 14 | USA(2016) | CP016923.1 | - |  |  |  |  |  |  |  |  |  |  |  |  |  |  |  |  |  |
| 46 | I-E* | PittNDM01 | AR_0068 | Non-CC258 | 14 | USA(2016) | CP020067.1 | - |  |  |  |  |  |  |  |  |  |  |  |  |  |  |  |  |  |
| 47 | I-E* | PittNDM01 | KPN528 | Non-CC258 | 14 | USA(2016) | CP020853.1 | - |  |  |  |  |  |  |  |  |  |  |  |  |  |  |  |  |  |
| 48 | I-E* | PittNDM01 | AR_0143 | Non-CC258 | 14 | USA(2016) | CP021708.1 | - |  |  |  |  |  |  |  |  |  |  |  |  |  |  |  |  |  |
| 49 | I-E* | PittNDM01 | AR_0066 | Non-CC258 | 14 | USA(2016) | CP026751.1 | - |  |  |  |  |  |  |  |  |  |  |  |  |  |  |  |  |  |
| 50 | I-E* | NUHL24835** | NUHL24835 | Non-CC258 | 14 | USA(2016) | CP014004.1 | - |  |  |  |  |  |  |  |  |  |  |  |  |  |  |  |  |  |

| 51 | I-E* | NUHL24835 | 23 | Non-CC258 | 14 | USA(2016) | CP016926.1 | - |  |  |  |  |  |  |  |  |  |  |  |  |  |  |  |  |  |
| --- | --- | --- | --- | --- | --- | --- | --- | --- | --- | --- | --- | --- | --- | --- | --- | --- | --- | --- | --- | --- | --- | --- | --- | --- | --- |
| 52 | I-E* | 19051 | 19051 | Non-CC258 | 15 | USA(2016) | CP022023.1 | - |  |  |  |  |  |  |  |  |  |  |  |  |  |  |  |  |  |
| 53 | I-E* | PMK1** | BR# | Non-CC258 | 15 | USA(2016) | CP015990.1 | CP015991.1 | repN | 0 | 0 | 0 | 0 | 0 | 0 | 0 | 0 | 0 | 0 | 0 | 0 | 0 | 0 | 0 | 0 |
| 54 | I-E* | PMK1 | PMK1 | Non-CC258 | 15 | USA(2016) | CP008929.1 | - |  |  |  |  |  |  |  |  |  |  |  |  |  |  |  |  |  |
| 55 | I-E* | PMK1 | KP36 | Non-CC258 | 15 | USA(2016) | CP017385.1 | - |  |  |  |  |  |  |  |  |  |  |  |  |  |  |  |  |  |
| 56 | I-E* | PMK1 | DHQP1605752_ | Non-CC258 | 15 | USA(2016) | CP022127.1 | - |  |  |  |  |  |  |  |  |  |  |  |  |  |  |  |  |  |
| 57 | I-E* | RJF999 | RJF999 | Non-CC258 | 23 | USA(2016) | CP014010.1 | - |  |  |  |  |  |  |  |  |  |  |  |  |  |  |  |  |  |
| 58 | I-E* | RJA166 | RJA166 | Non-CC258 | 23 | USA(2016) | CP019047.1 | - |  |  |  |  |  |  |  |  |  |  |  |  |  |  |  |  |  |
| 59 | I-E* | 1084** | 1084 | Non-CC258 | 23 | USA(2016) | CP003785.1 | - |  |  |  |  |  |  |  |  |  |  |  |  |  |  |  |  |  |
| 60 | I-E* | 1084 | ED2 | Non-CC258 | 23 | USA(2016) | CP016813.1 | - |  |  |  |  |  |  |  |  |  |  |  |  |  |  |  |  |  |
| 61 | I-E* | 1084 | SGH10 | Non-CC258 | 23 | USA(2016) | CP025080.1 | - |  |  |  |  |  |  |  |  |  |  |  |  |  |  |  |  |  |
| 62 | I-E* | P1428 | P1428 | Non-CC258 | 23 | USA(2016) | CP017994.1 | - |  |  |  |  |  |  |  |  |  |  |  |  |  |  |  |  |  |
| 63 | I-E* | KPHS1249 | KPHS1249 | Non-CC258 | 23 | USA(2016) | CP027189.1 | - |  |  |  |  |  |  |  |  |  |  |  |  |  |  |  |  |  |
| 64 | I-E* | ED23 | ED23 | Non-CC258 | 23 | USA(2016) | CP016814.1 | - |  |  |  |  |  |  |  |  |  |  |  |  |  |  |  |  |  |
| 65 | I-E* | NTUH-K2044 | NTUH-K2044 | Non-CC258 | 23 | USA(2016) | AP006725.1 | - |  |  |  |  |  |  |  |  |  |  |  |  |  |  |  |  |  |
| 66 | I-E* | J1 | J1 | Non-CC258 | 111 | USA(2016) | CP013711.1 | - |  |  |  |  |  |  |  |  |  |  |  |  |  |  |  |  |  |
| 67 | I-E* | 342 | 342 | Non-CC258 | 146 | USA(2016) | CP000964.1 | - |  |  |  |  |  |  |  |  |  |  |  |  |  |  |  |  |  |
| 68 | I-E* | AR_0117 | AR_0117 | Non-CC258 | 234 | USA(2016) | CP020061.1 | CP020066.1 | IncN | 0 | 0 | 0 | 0 | 0 | 0 | 0 | 0 | 0 | 0 | 0 | 0 | 0 | 0 | 0 | 0 |
| 69 | I-E* | RJF293 | RJF293 | Non-CC258 | 374 | USA(2016) | CP014008.1 | - |  |  |  |  |  |  |  |  |  |  |  |  |  |  |  |  |  |
| 70 | I-E* | ATCC 43816 | ATCC 43816 | Non-CC258 | 493 | USA(2016) | CP009208.1 | - |  |  |  |  |  |  |  |  |  |  |  |  |  |  |  |  |  |
| 71 | I-E* | ATCC 35657 | ATCC 35657 | Non-CC258 | 505 | USA(2016) | CP015134.1 | - |  |  |  |  |  |  |  |  |  |  |  |  |  |  |  |  |  |
| 72 |  | - | Kp_Goe_39795 | Non-CC258 | 15 | Germany（2016 | ）CP018458.1 | - |  |  |  |  |  |  |  |  |  |  |  |  |  |  |  |  |  |
| 73 |  | - | QS17-0029 | Non-CC258 | 16 | Thailand(2017) | CP024038.1 | - |  |  |  |  |  |  |  |  |  |  |  |  |  |  |  |  |  |
| 74 |  | - | UCLAOXA232K | Non-CC258 | 16 | USA(2015) | CP012560.1 | - |  |  |  |  |  |  |  |  |  |  |  |  |  |  |  |  |  |
| 75 |  | - | UCLAOXA232K | Non-CC258 | 16 | USA(2015) | CP012568.1 | - |  |  |  |  |  |  |  |  |  |  |  |  |  |  |  |  |  |
| 76 |  | - | FDAARGOS_44 | Non-CC258 | 16 | USA（2017） | CP023919.1 | - |  |  |  |  |  |  |  |  |  |  |  |  |  |  |  |  |  |
| 77 |  | - | XH209 | Non-CC258 | 17 | China (before 201 | CP009461.1 | - |  |  |  |  |  |  |  |  |  |  |  |  |  |  |  |  |  |
| 78 |  | - | Kp_Goe_154414 | Non-CC258 | 23 | Germany（2016 | ）CP018337.1 | - |  |  |  |  |  |  |  |  |  |  |  |  |  |  |  |  |  |
| 79 |  | - | KP-1 | Non-CC258 | 29 | Australia | CP012883.1 | - |  |  |  |  |  |  |  |  |  |  |  |  |  |  |  |  |  |
| 80 |  | - | INF322 | Non-CC258 | 29 | Australia(2017) | CP024482.1 | - |  |  |  |  |  |  |  |  |  |  |  |  |  |  |  |  |  |
| 81 |  | - | INF249 | Non-CC258 | 29 | Australia(2017) | CP024489.1 | - |  |  |  |  |  |  |  |  |  |  |  |  |  |  |  |  |  |
| 82 |  | - | KpvK54 | Non-CC258 | 29 | UK(2017) | CP023134.2 | - |  |  |  |  |  |  |  |  |  |  |  |  |  |  |  |  |  |
| 83 |  | - | DHQP1002001 | Non-CC258 | 34 | USA(2016) | CP016811.1 | - |  |  |  |  |  |  |  |  |  |  |  |  |  |  |  |  |  |
| 84 |  | - | KPNIH39 | Non-CC258 | 37 | USA(2016) | CP014762.1 | CP014765.1 | IncFIIK | 10 | 12 | 1 | 3 | 0 | 0 | 1 | 1 | 1 | 0 | 1 | 1 | 1 | 0 | 1 | 1 |
| 85 |  | - | KSB1_7J | Non-CC258 | 37 | Australia | CP024548.1 | - |  |  |  |  |  |  |  |  |  |  |  |  |  |  |  |  |  |
| 86 |  | - | 459 | Non-CC258 | 37 | China（2016） | CP018306.1 | - |  |  |  |  |  |  |  |  |  |  |  |  |  |  |  |  |  |
| 87 |  | - | KPN1482 | Non-CC258 | 37 | USA（2017） | CP020841.1 | - |  |  |  |  |  |  |  |  |  |  |  |  |  |  |  |  |  |
| 88 |  | - | AR_0139 | Non-CC258 | 37 | USA（2017） | CP021960.1 | - |  |  |  |  |  |  |  |  |  |  |  |  |  |  |  |  |  |
| 89 |  | - | INF042 | Non-CC258 | 37 | Australia(2017) | CP024542.1 | - |  |  |  |  |  |  |  |  |  |  |  |  |  |  |  |  |  |
| 90 |  | - | INF059 | Non-CC258 | 37 | Australia(2017) | CP024545.1 | - |  |  |  |  |  |  |  |  |  |  |  |  |  |  |  |  |  |
| 91 |  | - | MGH 78578 | Non-CC258 | 38 | USA (1994) | CP000647.1 | - |  |  |  |  |  |  |  |  |  |  |  |  |  |  |  |  |  |
| 92 |  | - | 1158 | Non-CC258 | 65 | China (2002) | CP006722.1 | - |  |  |  |  |  |  |  |  |  |  |  |  |  |  |  |  |  |
| 93 |  | - | NUHL30457 | Non-CC258 | 86 | China(2018) | CP026586.1 | CP026589.1 | IncFII | 2 | 2 | 0 | 0 | 1 | 0 | 0 | 0 | 0 | 1 | 0 | 0 | 0 | 0 | 0 | 0 |
| 94 |  | - | CG43 | Non-CC258 | 86 | China | CP006648.1 | - |  |  |  |  |  |  |  |  |  |  |  |  |  |  |  |  |  |
| 95 |  | - | KP5-1 | Non-CC258 | 86 | USA (2005) | CP008700.1 | - |  |  |  |  |  |  |  |  |  |  |  |  |  |  |  |  |  |
| 96 |  | - | HK787 | Non-CC258 | 86 | China | CP006738.1 | - |  |  |  |  |  |  |  |  |  |  |  |  |  |  |  |  |  |
| 97 |  | - | kp757 | Non-CC258 | 86 | USA(2016) | CP015120.1 | - |  |  |  |  |  |  |  |  |  |  |  |  |  |  |  |  |  |
| 98 |  | - | Kp_Goe_33208 | Non-CC258 | 101 | Germany(2016) | CP018447.1 | - |  |  |  |  |  |  |  |  |  |  |  |  |  |  |  |  |  |
| 99 |  | - | Kp_Goe_71070 | Non-CC258 | 101 | Germany(2016) | CP018450.1 | - |  |  |  |  |  |  |  |  |  |  |  |  |  |  |  |  |  |
| 100 |  | - | Kp_Goe_121641 | Non-CC258 | 101 | Germany（2016 | ）CP018735.1 | - |  |  |  |  |  |  |  |  |  |  |  |  |  |  |  |  |  |
| 101 |  | - | ST101:9601867  33 | Non-CC258 | 101 | South Africa(2016 | CP023487.1 | - |  |  |  |  |  |  |  |  |  |  |  |  |  |  |  |  |  |
| 102 |  | - | FDAARGOS_44 | Non-CC258 | 152 | USA（2017） | CP023949.1 | CP023952.1 | IncA/C | 0 | 0 | 0 | 0 | 0 | 0 | 0 | 0 | 0 | 0 | 0 | 0 | 0 | 0 | 0 | 0 |
| 103 |  | - | KPNIH48 | Non-CC258 | 252 | USA（2018） | CP026392.1 | CP026395.1,C | UT/IncFIIS | 0 | 0 | 0 | 0 | 0 | 0 | 0 | 0 | 0 | 0 | 0 | 0 | 0 | 0 | 0 | 0 |
| 104 |  | - | KPNIH50 | Non-CC258 | 252 | USA（2018） | CP026177.1 | CP026175.1 | A058 | 12 | 15 | 2 | 3 | 1 | 1 | 0 | 1 | 1 | 0 | 1 | 1 | 1 | 1 | 1 | 1 |
| 105 |  | - | KpN01 | Non-CC258 | 278 | Canada (2013) | CP012987.1 | - |  |  |  |  |  |  |  |  |  |  |  |  |  |  |  |  |  |
| 106 |  | - | KpN06 | Non-CC258 | 278 | Canada (2013) | CP012992.1 | - |  |  |  |  |  |  |  |  |  |  |  |  |  |  |  |  |  |
| 107 |  | - | KP1766 | Non-CC258 | 307 | USA（2017） | CP025146.1 | CP025148.1,C | pilL/pilL | 11 | 15 | 2 | 4 | 1 | 0 | 1 | 1 | 1 | 0 | 1 | 1 | 0 | 1 | 1 | 1 |
| 108 |  | - | NR5632 | Non-CC258 | 307 | USA（2017） | CP025143.1 | CP025144.1 | IncA/C | 0 | 0 | 0 | 0 | 0 | 0 | 0 | 0 | 0 | 0 | 0 | 0 | 0 | 0 | 0 | 0 |

| 109 |  | - | KP1768 | Non-CC258 | 307 | USA（2017） | CP025140.1 | CP025141.1 | IncA/C | 0 | 0 | 0 | 0 | 0 | 0 | 0 | 0 | 0 | 0 | 0 | 0 | 0 | 0 | 0 | 0 |
| --- | --- | --- | --- | --- | --- | --- | --- | --- | --- | --- | --- | --- | --- | --- | --- | --- | --- | --- | --- | --- | --- | --- | --- | --- | --- |
| 110 |  | - | KSB1_7E | Non-CC258 | 323 | Australia(2017) | CP024496.1 | - |  |  |  |  |  |  |  |  |  |  |  |  |  |  |  |  |  |
| 111 |  | - | KSB1_4E | Non-CC258 | 323 | Australia(2017) | CP024499.1 | - |  |  |  |  |  |  |  |  |  |  |  |  |  |  |  |  |  |
| 112 |  | - | KSB2_1B | Non-CC258 | 323 | Australia(2017) | CP024504.1 | - |  |  |  |  |  |  |  |  |  |  |  |  |  |  |  |  |  |
| 113 |  | - | KSB1_10J | Non-CC258 | 323 | Australia(2017) | CP024515.1 | - |  |  |  |  |  |  |  |  |  |  |  |  |  |  |  |  |  |
| 114 |  | - | KCTC 2242 | Non-CC258 | 375 | Korea | CP002910.1 | - |  |  |  |  |  |  |  |  |  |  |  |  |  |  |  |  |  |
| 115 |  | - | blaNDM-1 | Non-CC258 | 395 | USA (2011-2013) | CP009114.1 | - |  |  |  |  |  |  |  |  |  |  |  |  |  |  |  |  |  |
| 116 |  | - | Kp_Goe_62629 | Non-CC258 | 395 | Germany(2016) | CP018364.1 | - |  |  |  |  |  |  |  |  |  |  |  |  |  |  |  |  |  |
| 117 |  | - | Kp13 | Non-CC258 | 442 | Brazil(2009) | CP003999.1 | CP003997.1 | parB | 0 | 0 | 0 | 0 | 0 | 0 | 0 | 0 | 0 | 0 | 0 | 0 | 0 | 0 | 0 | 0 |
| 118 |  | - | 234-12 | Non-CC258 | 514 | Germany (2011) | CP011313.1 | - |  |  |  |  |  |  |  |  |  |  |  |  |  |  |  |  |  |
| 119 |  | - | H11 | Non-CC258 | 659 | China（2016） | CP018056.1 | - |  |  |  |  |  |  |  |  |  |  |  |  |  |  |  |  |  |
| 120 |  | - | AR_0107 | Non-CC258 | 1161 | USA（2017） | CP021955.1 | - |  |  |  |  |  |  |  |  |  |  |  |  |  |  |  |  |  |
| 121 |  | - | KPNIH29 | Non-CC258 | 1518 | USA (2013) | CP009863.1 | CP009864.1 | korA, repN, tra | 0 | 0 | 0 | 0 | 0 | 0 | 0 | 0 | 0 | 0 | 0 | 0 | 0 | 0 | 0 | 0 |
| 122 |  | - | W14 | Non-CC258 | 1536 | China(2016) | CP015753.1 | - |  |  |  |  |  |  |  |  |  |  |  |  |  |  |  |  |  |
| 123 |  | - | TH1 | Non-CC258 | 1536 | China(2016) | CP016159.1 | - |  |  |  |  |  |  |  |  |  |  |  |  |  |  |  |  |  |
| 124 |  | - | 1756 | Non-CC258 | 2549 | Taiwan(2017) | CP019219.1 | - |  |  |  |  |  |  |  |  |  |  |  |  |  |  |  |  |  |
| 125 |  | - | HKUOPLC | Non-CC258 | ND | China (2013) | CP012300.1 | - |  |  |  |  |  |  |  |  |  |  |  |  |  |  |  |  |  |
| 126 |  | - | ATCC BAA-214 | CC258 | 11 | USA (2010) | CP006659.2 | - |  |  |  |  |  |  |  |  |  |  |  |  |  |  |  |  |  |
| 127 |  | - | SWU01 | CC258 | 11 | China(2016) | CP018454.1 | CP018455.1 | IncFII | 10 | 14 | 2 | 3 | 2 | 1 | 0 | 1 | 1 | 1 | 1 | 1 | 0 | 0 | 1 | 0 |
| 128 |  | - | WCHKP8F4 | CC258 | 11 | China(2018) | CP027068.2 | CP027067.1 | IncFII | 7 | 11 | 2 | 3 | 2 | 1 | 0 | 1 | 1 | 0 | 1 | 0 | 0 | 0 | 0 | 0 |
| 129 |  | - | WCHKP649 | CC258 | 11 | China(2018) | CP026585.2 | CP026584.1 | IncFII | 10 | 14 | 2 | 3 | 2 | 1 | 0 | 1 | 1 | 1 | 1 | 1 | 0 | 0 | 1 | 0 |
| 130 |  | - | GD4 | CC258 | 11 | Hong Kong(2017) | CP025951.1 | CP025952.1 | IncFII | 11 | 15 | 2 | 3 | 2 | 1 | 1 | 1 | 1 | 1 | 1 | 1 | 0 | 0 | 1 | 0 |
| 131 |  | - | K. pneumoniae 1 | CC258 | 11 | China (2009) | CP025466.1 | CP025468.1 | IncFIIK | 10 | 13 | 2 | 3 | 1 | 1 | 0 | 1 | 1 | 0 | 1 | 1 | 1 | 0 | 1 | 0 |
| 132 |  | - | K. pneumoniae F | CC258 | 11 | China (2016) | CP025461.1 | CP025463.1 | IncFII | 8 | 12 | 2 | 3 | 2 | 1 | 0 | 1 | 1 | 1 | 1 | 0 | 0 | 0 | 0 | 0 |
| 133 |  | - | FDAARGOS_44 | CC258 | 11 | USA（2017） | CP023941.1 | CP023942.1 | IncFII | 10 | 15 | 2 | 3 | 2 | 1 | 0 | 1 | 1 | 2 | 1 | 1 | 0 | 0 | 1 | 0 |
| 134 |  | - | FDAARGOS_44 | CC258 | 11 | USA（2017） | CP023933.1 | CP023938.1 | A054 | 7 | 11 | 2 | 3 | 2 | 1 | 0 | 1 | 1 | 0 | 1 | 0 | 0 | 0 | 0 | 0 |
| 135 |  | - | CAV1392 | CC258 | 11 | UK(2011) | CP011578.1 | CP011575.1 | trbA | 0 | 0 | 0 | 0 | 0 | 0 | 0 | 0 | 0 | 0 | 0 | 0 | 0 | 0 | 0 | 0 |
| 136 |  | - | JM45 | CC258 | 11 | China (2010) | CP006656.1 | CP006657.1 | IncFIIK | 11 | 15 | 2 | 4 | 1 | 0 | 1 | 1 | 1 | 0 | 1 | 1 | 0 | 1 | 1 | 1 |
| 137 |  | - | HS11286 | CC258 | 11 | China (2011) | CP003200.1 | CP003224.1 | IncFIIK | 11 | 15 | 2 | 4 | 1 | 1 | 0 | 1 | 1 | 0 | 1 | 1 | 1 | 1 | 1 | 0 |
| 138 |  | - | KP38731 | CC258 | 11 | USA(2016) | CP014294.1 | - |  |  |  |  |  |  |  |  |  |  |  |  |  |  |  |  |  |
| 139 |  | - | Kp_Goe_822917 | CC258 | 11 | Germany(2016) | CP018438.1 | - |  |  |  |  |  |  |  |  |  |  |  |  |  |  |  |  |  |
| 140 |  | - | Kp_Goe_821588 | CC258 | 11 | Germany（2016 | ）CP018692.1 | - |  |  |  |  |  |  |  |  |  |  |  |  |  |  |  |  |  |
| 141 |  | - | AR_0049 | CC258 | 11 | USA（2016） | CP018816.1 | - |  |  |  |  |  |  |  |  |  |  |  |  |  |  |  |  |  |
| 142 |  | - | K66-45 | CC258 | 11 | Norway(2017) | CP020901.1 | - |  |  |  |  |  |  |  |  |  |  |  |  |  |  |  |  |  |
| 143 |  | - | AR_0146 | CC258 | 11 | USA（2017） | CP021685.1 | - |  |  |  |  |  |  |  |  |  |  |  |  |  |  |  |  |  |
| 144 |  | - | AR_0148 | CC258 | 11 | USA（2017） | CP021950.1 | - |  |  |  |  |  |  |  |  |  |  |  |  |  |  |  |  |  |
| 145 |  | - | 911021 | CC258 | 11 | China(2017) | CP022882.1 | - |  |  |  |  |  |  |  |  |  |  |  |  |  |  |  |  |  |
| 146 |  | - | 721005 | CC258 | 11 | China(2017) | CP022997.1 | - |  |  |  |  |  |  |  |  |  |  |  |  |  |  |  |  |  |
| 147 |  | - | 500_1420 | CC258 | 258 | USA(2004-2012) | CP011980.1 | CP011981.1 | IncFIIK | 10 | 13 | 2 | 3 | 1 | 0 | 0 | 1 | 1 | 0 | 1 | 1 | 0 | 1 | 1 | 1 |
| 148 |  | - | AR_0361 | CC258 | 258 | USA（2018） | CP027160.1 | CP027158.1 | IncFIIK | 8 | 8 | 1 | 1 | 0 | 0 | 0 | 1 | 1 | 0 | 1 | 1 | 1 | 0 | 1 | 0 |
| 149 |  | - | NU-CRE047 | CC258 | 258 | USA（2017） | CP025037.1 | CP025039.1 | IncFIA | 4 | 4 | 0 | 0 | 1 | 0 | 0 | 0 | 0 | 0 | 0 | 1 | 1 | 0 | 1 | 0 |
| 150 |  | - | AUSMDU00008 | CC258 | 258 | Australia(2017) | CP025008.1 | CP025010.1 | IncFIIK | 8 | 8 | 1 | 1 | 0 | 0 | 0 | 1 | 1 | 0 | 1 | 1 | 1 | 0 | 1 | 0 |
|  |  |  |  |  |  |  |  | ,CP025009.1 | IncFIIK | 10 | 14 | 2 | 4 | 1 | 0 | 1 | 1 | 1 | 0 | 1 | 1 | 0 | 0 | 1 | 1 |
| 151 |  | - | AUSMDU00003 | CC258 | 258 | Australia(2017) | CP025005.1 | CP025006.1 | IncFIIK | 10 | 14 | 2 | 4 | 1 | 0 | 1 | 1 | 1 | 0 | 1 | 1 | 0 | 0 | 1 | 1 |
| 152 |  | - | AUSMDU00008 | CC258 | 258 | Australia (2012) | CP022691.1 | CP022693.1 | IncFIIK | 10 | 14 | 2 | 4 | 1 | 0 | 1 | 1 | 1 | 0 | 1 | 1 | 0 | 0 | 1 | 1 |
| 153 |  | - | BIC-1 | CC258 | 258 | France (2009) | CP022573.1 | CP022574.1 | IncFIIK | 10 | 14 | 2 | 4 | 1 | 0 | 1 | 1 | 1 | 0 | 1 | 1 | 0 | 0 | 1 | 1 |
| 154 |  | - | AR_0125 | CC258 | 258 | USA（2017） | CP021859.1 | CP021861.1 | UT | 0 | 0 | 0 | 0 | 0 | 0 | 0 | 0 | 0 | 0 | 0 | 0 | 0 | 0 | 0 | 0 |
|  |  |  |  |  |  |  |  | ,CP021860.1 | A007 | 0 | 0 | 0 | 0 | 0 | 0 | 0 | 0 | 0 | 0 | 0 | 0 | 0 | 0 | 0 | 0 |
| 155 |  | - | AR_0120 | CC258 | 258 | USA（2017） | CP021833.1 | CP021835.1 | IncA/C | 0 | 0 | 0 | 0 | 0 | 0 | 0 | 0 | 0 | 0 | 0 | 0 | 0 | 0 | 0 | 0 |
| 156 |  | - | AR_0113 | CC258 | 258 | USA（2017） | CP021751.1 | CP021756.1 | A007 | 0 | 0 | 0 | 0 | 0 | 0 | 0 | 0 | 0 | 0 | 0 | 0 | 0 | 0 | 0 | 0 |
|  |  |  |  |  |  |  |  | ,CP021754.1 | UT | 0 | 0 | 0 | 0 | 0 | 0 | 0 | 0 | 0 | 0 | 0 | 0 | 0 | 0 | 0 | 0 |
| 157 |  | - | AR_0129 | CC258 | 258 | USA（2017） | CP021718.1 | CP021716.1 | HCM1_259 | 0 | 0 | 0 | 0 | 0 | 0 | 0 | 0 | 0 | 0 | 0 | 0 | 0 | 0 | 0 | 0 |
| 158 |  | - | AR_0112 | CC258 | 258 | USA（2017） | CP021549.1 | CP021546.1 | UT | 0 | 0 | 0 | 0 | 0 | 0 | 0 | 0 | 0 | 0 | 0 | 0 | 0 | 0 | 0 | 0 |
|  |  |  |  |  |  |  |  | ,CP021548.1 | A007 | 0 | 0 | 0 | 0 | 0 | 0 | 0 | 0 | 0 | 0 | 0 | 0 | 0 | 0 | 0 | 0 |
| 159 |  | - | AR_0098 | CC258 | 258 | USA（2017） | CP020108.1 | CP020110.1 | repN | 0 | 0 | 0 | 0 | 0 | 0 | 0 | 0 | 0 | 0 | 0 | 0 | 0 | 0 | 0 | 0 |
| 160 |  | - | AR_0115 | CC258 | 258 | USA（2017） | CP020071.1 | CP020075.1 | traJ | 0 | 0 | 0 | 0 | 0 | 0 | 0 | 0 | 0 | 0 | 0 | 0 | 0 | 0 | 0 | 0 |
| 161 |  | - | MNCRE53 | CC258 | 258 | USA (2012) | CP018437.1 | CP018436.1 | HCM1_259 | 0 | 0 | 0 | 0 | 0 | 0 | 0 | 0 | 0 | 0 | 0 | 0 | 0 | 0 | 0 | 0 |
| 162 |  | - | MNCRE78 | CC258 | 258 | USA (2013) | CP018428.1 | CP018432.1 | HCM1_259 | 0 | 0 | 0 | 0 | 0 | 0 | 0 | 0 | 0 | 0 | 0 | 0 | 0 | 0 | 0 | 0 |

| 163 |  | - | MNCRE69 | CC258 | 258 | USA (before 2016 | CP018427.1 | CP018426.1 | HCM1_259 | 0 | 0 | 0 | 0 | 0 | 0 | 0 | 0 | 0 | 0 | 0 | 0 | 0 | 0 | 0 | 0 |
| --- | --- | --- | --- | --- | --- | --- | --- | --- | --- | --- | --- | --- | --- | --- | --- | --- | --- | --- | --- | --- | --- | --- | --- | --- | --- |
| 164 |  | - | CR14 | CC258 | 258 | USA(2016) | CP015392.1 | CP015395.1 | IncA/C | 0 | 0 | 0 | 0 | 0 | 0 | 0 | 0 | 0 | 0 | 0 | 0 | 0 | 0 | 0 | 0 |
| 165 |  | - | KPNIH36 | CC258 | 258 | USA(2016) | CP014647.1 | CP014650.1 | IncFIIK | 8 | 8 | 1 | 1 | 0 | 0 | 0 | 1 | 1 | 0 | 1 | 1 | 1 | 0 | 1 | 0 |
| 166 |  | - | UHKPC33 | CC258 | 258 | USA(2004-2012) | CP011989.1 | CP011990.1 | IncFIIK | 11 | 15 | 2 | 4 | 1 | 0 | 1 | 1 | 1 | 0 | 1 | 1 | 0 | 1 | 1 | 1 |
| 167 |  | - | UHKPC07 | CC258 | 258 | USA(2004-2012) | CP011985.1 | CP011986.1 | IncFIIK | 8 | 8 | 1 | 1 | 0 | 0 | 0 | 1 | 1 | 0 | 1 | 1 | 1 | 0 | 1 | 0 |
| 168 |  | - | DMC1097 | CC258 | 258 | USA(2004-2012) | CP011976.1 | CP011978.1 | UT | 0 | 0 | 0 | 0 | 0 | 0 | 0 | 0 | 0 | 0 | 0 | 0 | 0 | 0 | 0 | 0 |
| 169 |  | - | CAV1596 | CC258 | 258 | USA (2012) | CP011647.1 | CP011646.1 | trbA | 1 | 1 | 0 | 0 | 0 | 0 | 0 | 0 | 0 | 0 | 0 | 0 | 0 | 0 | 0 | 1 |
|  |  |  |  |  |  |  |  | ,CP011645.1 | repN | 0 | 0 | 0 | 0 | 0 | 0 | 0 | 0 | 0 | 0 | 0 | 0 | 0 | 0 | 0 | 0 |
| 170 |  | - | 32192 | CC258 | 258 | USA (2010) | CP010361.1 | CP010575.1 | repN | 0 | 0 | 0 | 0 | 0 | 0 | 0 | 0 | 0 | 0 | 0 | 0 | 0 | 0 | 0 | 0 |
| 171 |  | - | 34618 | CC258 | 258 | USA (2011) | CP010392.1 | CP010396.1 | repN | 0 | 0 | 0 | 0 | 0 | 0 | 0 | 0 | 0 | 0 | 0 | 0 | 0 | 0 | 0 | 0 |
| 172 |  | - | KPNIH30 | CC258 | 258 | USA (2013) | CP009872.1 | CP009875.1 | IncFIIK | 8 | 8 | 1 | 1 | 0 | 0 | 0 | 1 | 1 | 0 | 1 | 1 | 1 | 0 | 1 | 0 |
| 173 |  | - | KPNIH32 | CC258 | 258 | USA (2013) | CP009775.1 | CP009776.1 | IncFIA | 1 | 1 | 0 | 0 | 1 | 0 | 0 | 0 | 0 | 0 | 0 | 0 | 0 | 0 | 0 | 0 |
| 174 |  | - | KPNIH33 | CC258 | 258 | USA (2013) | CP009771.1 | CP009773.1 | IncFIA | 1 | 1 | 0 | 0 | 1 | 0 | 0 | 0 | 0 | 0 | 0 | 0 | 0 | 0 | 0 | 0 |
| 175 |  | - | KPR0928 | CC258 | 258 | USA (2012) | CP008831.1 | CP008833.1 | IncFIIK | 10 | 15 | 1 | 4 | 0 | 0 | 3 | 1 | 1 | 0 | 1 | 1 | 1 | 1 | 1 | 0 |
| 176 |  | - | KPNIH1 | CC258 | 258 | USA (2011) | CP008827.1 | CP008830.1 | IncFIIK | 8 | 8 | 1 | 1 | 0 | 0 | 0 | 1 | 1 | 0 | 1 | 1 | 1 | 0 | 1 | 0 |
| 177 |  | - | KPNIH24 | CC258 | 258 | USA (2012) | CP008797.1 | CP008798.1 | repN | 0 | 0 | 0 | 0 | 0 | 0 | 0 | 0 | 0 | 0 | 0 | 0 | 0 | 0 | 0 | 0 |
| 178 |  | - | KPNIH10 | CC258 | 258 | USA (2011) | CP007727.1 | CP007730.1 | IncFIIK | 10 | 15 | 1 | 4 | 0 | 0 | 3 | 1 | 1 | 0 | 1 | 1 | 1 | 1 | 1 | 0 |
| 179 |  | - | 30660/NJST258_ | CC258 | 258 | USA (2010) | CP006923.1 | CP006926.1 | IncFIA | 6 | 7 | 1 | 2 | 0 | 0 | 0 | 0 | 0 | 0 | 0 | 1 | 0 | 1 | 1 | 1 |
| 180 |  | - | 30684/NJST258_ | CC258 | 258 | USA (2010) | CP006918.1 | CP006919.1 | A011 | 0 | 0 | 0 | 0 | 0 | 0 | 0 | 0 | 0 | 0 | 0 | 0 | 0 | 0 | 0 | 0 |
| 181 |  | - | CAV1453 | CC258 | 258 | UK(2017) | CP018356.1 | - |  |  |  |  |  |  |  |  |  |  |  |  |  |  |  |  |  |
| 182 |  | - | BK13043 | CC258 | 258 | USA（2017） | CP020837.1 | - |  |  |  |  |  |  |  |  |  |  |  |  |  |  |  |  |  |
| 183 |  | - | AR_0047 | CC258 | 258 | USA（2017） | CP021539.1 | - |  |  |  |  |  |  |  |  |  |  |  |  |  |  |  |  |  |
| 184 |  | - | FDAARGOS_44 | CC258 | 340 | Canada (2013) | CP023502.1 | - |  |  |  |  |  |  |  |  |  |  |  |  |  |  |  |  |  |
| 185 |  | - | CAV1042 | CC258 | 340 | USA (2008) | CP018671.1 | in chromosome |  |  |  |  |  |  |  |  |  |  |  |  |  |  |  |  |  |
| 186 |  | - | CAV1217 | CC258 | 340 | USA (2010) | CP018676.1 | in chromosome |  |  |  |  |  |  |  |  |  |  |  |  |  |  |  |  |  |
| 187 |  | - | NY9 | CC258 | 340 | USA(2016) | CP015385.1 | CP015387.1 | IncFIA | 4 | 4 | 0 | 0 | 1 | 0 | 0 | 0 | 0 | 0 | 0 | 1 | 1 | 0 | 1 | 0 |
| 188 |  | - | INF274 | CC258 | 340 | Australia | CP024570.1 | - |  |  |  |  |  |  |  |  |  |  |  |  |  |  |  |  |  |
| 189 |  | - | INF278 | CC258 | 340 | Australia | CP024563.1 | - |  |  |  |  |  |  |  |  |  |  |  |  |  |  |  |  |  |
| 190 |  | - | INF164 | CC258 | 340 | Australia | CP024556.1 | - |  |  |  |  |  |  |  |  |  |  |  |  |  |  |  |  |  |
| 191 |  | - | INF163 | CC258 | 340 | Australia | CP024549.1 | - |  |  |  |  |  |  |  |  |  |  |  |  |  |  |  |  |  |
| 192 |  | - | CAV1417 | CC258 | 340 | UK(2016) | CP018352.1 | - |  |  |  |  |  |  |  |  |  |  |  |  |  |  |  |  |  |
| 193 |  | - | FDAARGOS_43 | CC258 | 340 | USA(2017) | CP023907.1 | - |  |  |  |  |  |  |  |  |  |  |  |  |  |  |  |  |  |
| 194 |  | - | FDAARGOS_44 | CC258 | 340 | USA(2017) | CP023946.1 | - |  |  |  |  |  |  |  |  |  |  |  |  |  |  |  |  |  |
| 195 |  | - | KSB1_5D | CC258 | 340 | Australia(2017) | CP024191.1 | - |  |  |  |  |  |  |  |  |  |  |  |  |  |  |  |  |  |
| 196 |  | - | INF158 | CC258 | 340 | Australia(2017) | CP024521.1 | - |  |  |  |  |  |  |  |  |  |  |  |  |  |  |  |  |  |
| 197 |  | - | INF157 | CC258 | 340 | Australia(2017) | CP024528.1 | - |  |  |  |  |  |  |  |  |  |  |  |  |  |  |  |  |  |
| 198 |  | - | KSB1_9D | CC258 | 340 | Australia(2017) | CP024535.1 | - |  |  |  |  |  |  |  |  |  |  |  |  |  |  |  |  |  |
| 199 |  | - | BR21 | CC258 | 437 | USA（2016） | CP018885.1 | CP018887.1 | korA, repN, tra | 0 | 0 | 0 | 0 | 0 | 0 | 0 | 0 | 0 | 0 | 0 | 0 | 0 | 0 | 0 | 0 |
| 200 |  | - | BR7 | CC258 | 437 | USA（2016） | CP018883.1 | CP018884.1 | korA | 0 | 0 | 0 | 0 | 0 | 0 | 0 | 0 | 0 | 0 | 0 | 0 | 0 | 0 | 0 | 0 |
| 201 |  | - | KPN_KPC_HUG | CC258 | 512 | Switzerland(2015) | CP019772.1 | CP019774.1 | IncFIIK | 8 | 8 | 1 | 1 | 0 | 0 | 0 | 1 | 1 | 0 | 1 | 1 | 1 | 0 | 1 | 0 |
| 202 |  | - | blood sample 2 | CC258 | 512 | Switzerland(2016) | CP015822.1 | - |  |  |  |  |  |  |  |  |  |  |  |  |  |  |  |  |  |
| 203 |  | - | K. pneumoniae 6 | CC258 | 1326 | China (2017) | CP025456.1 | CP025458.1 | IncFII | 7 | 11 | 2 | 3 | 2 | 1 | 0 | 1 | 1 | 0 | 1 | 0 | 0 | 0 | 0 | 0 |
