# Supplementary material for: The type I-E CRISPR-Cas system influences the acquisition of blaKPC-IncF plasmid in Klebsiella pneumonia
Source: Emerg Microbes Infect. 2020 May 20;9(1):1011–22. doi: 10.1080/22221751.2020.1763209 (PMC7301723; doi:10.1080/22221751.2020.1763209)
Supplement: Supplemental Material [file TEMI_A_1763209_SM1594.zip › Supplementary files/supplementary data1b.docx]

Supplementary data 1b. 121 *bla*_KPC_- positive plasmids in *K. pneumoniae* used in this study.

| No. | Name^a^ | GenBank accession no | Resistance gene | Typing | Number of the regions matching to the spacers of the *K. pneumoniae* CRISPR loci | | | | | | | | | | | | | | |
| --- | --- | --- | --- | --- | --- | --- | --- | --- | --- | --- | --- | --- | --- | --- | --- | --- | --- | --- | --- |
|  |  |  |  |  | Total | Spacer1 | Spacer2 | Spacer3 | Spacer4 | Spacer5 | Spacer6 | Spacer7 | Spacer8 | Spacer9 | Spacer10 | Spacer11 | Spacer12 | Spacer13 | Spacer14 |
| 1 | p187-2 | CP025468.1 | KPC | IncFIIK | 13 | 2 | 3 | 1 | 1 | 0 | 1 | 1 | 0 | 1 | 1 | 1 | 0 | 1 | 0 |
| 2 | p12139-KPC | MF168403.1 | KPC | IncFII | 23 | 2 | 6 | 2 | 2 | 4 | 1 | 1 | 1 | 1 | 1 | 0 | 1 | 1 | 0 |
| 3 | p64917-KPC | MF168405.1 | KPC | IncFII | 21 | 2 | 6 | 2 | 1 | 4 | 1 | 1 | 1 | 1 | 1 | 0 | 0 | 1 | 0 |
| 4 | pCT-KPC | KT185451.1 | KPC | IncFII | 19 | 2 | 5 | 2 | 1 | 3 | 1 | 1 | 0 | 1 | 1 | 0 | 1 | 1 | 0 |
| 5 | p0716-KPC | KY270849.1 | KPC | IncFIIY | 21 | 2 | 5 | 1 | 1 | 4 | 1 | 1 | 1 | 1 | 1 | 1 | 1 | 1 | 0 |
| 6 | p20049-KPC | MF168404.1 | KPC | IncFII | 17 | 2 | 5 | 0 | 1 | 3 | 1 | 1 | 0 | 1 | 1 | 0 | 1 | 1 | 0 |
| 7 | pKpQIL-307_48 | KY271403.1 | KPC | IncFIIK | 21 | 2 | 4 | 0 | 0 | 4 | 2 | 2 | 0 | 2 | 1 | 1 | 1 | 2 | 0 |
| 8 | p12181-KPC | KY270850.1 | KPC | IncFIIY | 20 | 2 | 5 | 1 | 1 | 3 | 1 | 1 | 1 | 1 | 1 | 1 | 1 | 1 | 0 |
| 9 | pKP1034 | KP893385.1 | KPC | IncFII | 19 | 2 | 6 | 2 | 1 | 3 | 1 | 1 | 1 | 1 | 0 | 0 | 0 | 1 | 0 |
| 10 | pBIC-1a | CP022574.1 | KPC | IncFIIK | 16 | 2 | 4 | 1 | 0 | 3 | 1 | 1 | 0 | 1 | 1 | 0 | 1 | 1 | 0 |
| 11 | p44-2 | CP025463 | KPC | IncFII | 19 | 2 | 6 | 2 | 1 | 3 | 1 | 1 | 1 | 1 | 0 | 0 | 0 | 1 | 0 |
| 12 | p1068-KPC | MF168402.1 | KPC | IncFII | 19 | 2 | 5 | 2 | 1 | 3 | 1 | 1 | 0 | 1 | 1 | 0 | 1 | 1 | 0 |
| 13 | p283747-KPC | MF168406.1 | KPC | IncFII | 17 | 1 | 5 | 2 | 1 | 3 | 1 | 1 | 1 | 1 | 0 | 0 | 0 | 1 | 0 |
| 14 | pKPHS2 | CP003224.1 | KPC | IncFIIK | 18 | 2 | 4 | 1 | 1 | 3 | 1 | 1 | 0 | 1 | 1 | 1 | 1 | 1 | 0 |
| 15 | pHS091147 | KX236178.1 | KPC | IncFIIK | 19 | 2 | 4 | 1 | 1 | 4 | 1 | 1 | 0 | 1 | 1 | 1 | 1 | 1 | 0 |
| 16 | p628-KPC | KP987218.1 | KPC | IncFIIK | 17 | 2 | 4 | 1 | 0 | 3 | 1 | 1 | 0 | 1 | 1 | 1 | 1 | 1 | 0 |
| 17 | pGR-1504 | KF874496.1 | KPC | IncFIIK | 16 | 2 | 4 | 0 | 0 | 3 | 1 | 1 | 0 | 1 | 1 | 1 | 1 | 1 | 0 |
| 18 | pGR-1870 | KF874498.1 | KPC | IncFIIK | 18 | 2 | 4 | 1 | 0 | 4 | 1 | 1 | 0 | 1 | 1 | 1 | 1 | 1 | 0 |
| 19 | pKPC_CAV1217 | CP018675.1 | KPC | IncFIIK | 17 | 2 | 4 | 1 | 0 | 3 | 1 | 1 | 0 | 1 | 1 | 1 | 1 | 1 | 0 |
| 20 | pIT-01C03 | HG969995.1 | KPC | IncFIIK | 15 | 1 | 4 | 0 | 0 | 3 | 1 | 1 | 0 | 1 | 1 | 1 | 1 | 1 | 0 |
| 21 | pIT-11C07 | HG969998.1 | KPC | IncFIIK | 17 | 2 | 3 | 1 | 0 | 4 | 1 | 1 | 0 | 1 | 1 | 1 | 1 | 1 | 0 |
| 22 | pKpQIL-10 | KJ146687.1 | KPC | IncFIIK | 15 | 1 | 4 | 0 | 0 | 3 | 1 | 1 | 0 | 1 | 1 | 1 | 1 | 1 | 0 |
| 23 | pKpQIL-234 | KJ146689.1 | KPC | IncFIIK | 15 | 1 | 4 | 0 | 0 | 3 | 1 | 1 | 0 | 1 | 1 | 1 | 1 | 1 | 0 |
| 24 | pAUSMDU8079-2 | CP022693.1 | KPC | IncFIIK | 15 | 1 | 4 | 0 | 0 | 3 | 1 | 1 | 0 | 1 | 1 | 1 | 1 | 1 | 0 |
| 25 | pG12-KPC-2 | KU665642.1 | KPC | IncFIIK | 15 | 1 | 4 | 0 | 0 | 3 | 1 | 1 | 0 | 1 | 1 | 1 | 1 | 1 | 0 |
| 26 | pIT-01C22 | HG969997.1 | KPC | IncFIIK | 15 | 1 | 4 | 0 | 0 | 3 | 1 | 1 | 0 | 1 | 1 | 1 | 1 | 1 | 0 |
| 27 | pKpQIL | GU595196.1 | KPC | IncFIIK | 14 | 1 | 4 | 0 | 0 | 2 | 1 | 1 | 0 | 1 | 1 | 1 | 1 | 1 | 0 |
| 28 | p2 | CP019774.1 | KPC | IncFIIK | 15 | 1 | 4 | 0 | 0 | 3 | 1 | 1 | 0 | 1 | 1 | 1 | 1 | 1 | 0 |
| 29 | pGR-1780 | KF874497.2 | KPC | IncFIIK | 15 | 1 | 4 | 0 | 0 | 3 | 1 | 1 | 0 | 1 | 1 | 1 | 1 | 1 | 0 |
| 30 | pGR-3913 | KF874499.1 | KPC | IncFIIK | 15 | 1 | 4 | 0 | 0 | 3 | 1 | 1 | 0 | 1 | 1 | 1 | 1 | 1 | 0 |
| 31 | pIT-06C07 | LT009688.1 | KPC | IncFIIK | 16 | 2 | 4 | 1 | 0 | 2 | 1 | 1 | 0 | 1 | 1 | 1 | 1 | 1 | 0 |
| 32 | pKpQIL-D1 | KY798505.1 | KPC | IncFIIK | 16 | 2 | 4 | 0 | 0 | 3 | 1 | 1 | 0 | 1 | 1 | 1 | 1 | 1 | 0 |
| 33 | pKpQIL-IT | JN233705.2 | KPC | IncFIIK | 15 | 1 | 4 | 0 | 0 | 3 | 1 | 1 | 0 | 1 | 1 | 1 | 1 | 1 | 0 |
| 34 | pKpQIL-UK | KY798507.1 | KPC | IncFIIK | 15 | 1 | 4 | 0 | 0 | 3 | 1 | 1 | 0 | 1 | 1 | 1 | 1 | 1 | 0 |
| 35 | pUHKPC33 | CP011991.1 | KPC | IncFIIK | 15 | 1 | 4 | 0 | 0 | 3 | 1 | 1 | 0 | 1 | 1 | 1 | 1 | 1 | 0 |
| 36 | p69-2 | CP025458 | KPC | IncFII | 14 | 2 | 3 | 2 | 1 | 2 | 1 | 1 | 0 | 1 | 0 | 0 | 0 | 1 | 0 |
| 37 | p500_1420 | CP011981.1 | KPC | IncFIIK | 15 | 2 | 4 | 0 | 0 | 3 | 1 | 1 | 0 | 1 | 1 | 0 | 1 | 1 | 0 |
| 38 | pKp41 | CP012000.1 | KPC | IncFIIK | 15 | 1 | 4 | 0 | 0 | 3 | 1 | 1 | 0 | 1 | 1 | 1 | 1 | 1 | 0 |
| 39 | pVGH151 | KJ721790.1 | KPC | IncFIIK | 15 | 1 | 4 | 0 | 0 | 3 | 1 | 1 | 0 | 1 | 1 | 1 | 1 | 1 | 0 |
| 40 | pKpQIL-LS6 | JX442975.1 | KPC | IncFIB | 13 | 1 | 4 | 0 | 0 | 4 | 1 | 1 | 0 | 1 | 0 | 0 | 0 | 1 | 0 |
| 41 | pIT-FIPP-1 | HG969999.1 | KPC | IncFIB | 13 | 2 | 4 | 0 | 0 | 3 | 1 | 1 | 0 | 1 | 0 | 0 | 0 | 1 | 0 |
| 42 | pBK32179 | JX430448.1 | KPC | IncFIIK | 13 | 2 | 2 | 1 | 0 | 2 | 1 | 1 | 0 | 1 | 1 | 0 | 1 | 0 | 1 |
| 43 | pIT-12C47 | HG969996.1 | KPC | IncFIIK | 8 | 1 | 2 | 0 | 0 | 2 | 0 | 0 | 0 | 0 | 1 | 1 | 1 | 0 | 0 |
| 44 | PKPCAPSS | KP008371.1 | KPC | IncFIIK | 13 | 0 | 4 | 0 | 0 | 3 | 1 | 1 | 0 | 0 | 1 | 1 | 1 | 1 | 0 |
| 45 | p13294-KPC | MF156708.1 | KPC | IncFIIY | 9 | 1 | 3 | 0 | 0 | 3 | 0 | 0 | 0 | 0 | 1 | 1 | 0 | 0 | 0 |
| 46 | pUSKPC3 | KJ721789.1 | KPC | IncFIA | 8 | 0 | 2 | 1 | 0 | 2 | 0 | 0 | 0 | 0 | 1 | 1 | 1 | 0 | 0 |
| 47 | pNY9_2 | CP015387.1 | KPC | IncFIA | 7 | 0 | 2 | 1 | 0 | 1 | 0 | 0 | 0 | 0 | 1 | 1 | 1 | 0 | 0 |
| 48 | pBK30683 | KF954760.1 | KPC | IncFIA | 7 | 0 | 2 | 1 | 0 | 1 | 0 | 0 | 0 | 0 | 1 | 1 | 1 | 0 | 0 |
| 49 | pKP048 | FJ628167.2 | KPC | IncFIIK | 5 | 0 | 1 | 0 | 0 | 1 | 0 | 0 | 0 | 0 | 1 | 1 | 1 | 0 | 0 |
| 50 | pIT-12C73 | LT009689.1 | KPC | IncFIIK | 3 | 0 | 0 | 0 | 0 | 0 | 0 | 0 | 0 | 0 | 1 | 1 | 1 | 0 | 0 |
| 51 | pKPN101-IT | JX283456.1 | KPC | IncFIIK | 3 | 0 | 0 | 0 | 0 | 0 | 0 | 0 | 0 | 0 | 1 | 1 | 1 | 0 | 0 |
| 52 | pKp28 | CP011999.1 | KPC | IncFIA | 3 | 1 | 1 | 0 | 0 | 1 | 0 | 0 | 0 | 0 | 0 | 0 | 0 | 0 | 0 |
| 53 | pMNCRE44_5 | CP010881.1 | KPC | IncFIA | 3 | 0 | 1 | 1 | 0 | 1 | 0 | 0 | 0 | 0 | 0 | 0 | 0 | 0 | 0 |
| 54 | pBK30661 | KF954759.1 | KPC | IncFIA | 3 | 0 | 1 | 1 | 0 | 1 | 0 | 0 | 0 | 0 | 0 | 0 | 0 | 0 | 0 |

| 55 | pKPC-LK30 | KC405622.1 | KPC | UT | 17 | 2 | 5 | 1 | 1 | 3 | 1 | 1 | 1 | 1 | 0 | 0 | 0 | 1 | 0 |
| --- | --- | --- | --- | --- | --- | --- | --- | --- | --- | --- | --- | --- | --- | --- | --- | --- | --- | --- | --- |
| 56 | pKPC_P16 | KY689238.1 | KPC | UT | 15 | 1 | 5 | 1 | 1 | 2 | 1 | 1 | 1 | 1 | 0 | 0 | 0 | 1 | 0 |
| 57 | pKP1766_p2 | CP025148.1 | KPC | UT | 16 | 2 | 4 | 0 | 0 | 3 | 1 | 1 | 0 | 1 | 1 | 1 | 1 | 0 | 1 |
| 58 | pBK32533 | KP345882.1 | KPC | IncA/C | 3 | 0 | 1 | 1 | 0 | 1 | 0 | 0 | 0 | 0 | 0 | 0 | 0 | 0 | 0 |
| 59 | pWSZBR | CP015991.1 | KPC | repN | 0 | 0 | 0 | 0 | 0 | 0 | 0 | 0 | 0 | 0 | 0 | 0 | 0 | 0 | 0 |
| 60 | pCRKP-59-KPC | KX928752.1 | KPC | Inc FIA, FIB,FII | 0 | 0 | 0 | 0 | 0 | 0 | 0 | 0 | 0 | 0 | 0 | 0 | 0 | 0 | 0 |
| 61 | pSLMT | HQ589350.1 | KPC | IncFIIK | 0 | 0 | 0 | 0 | 0 | 0 | 0 | 0 | 0 | 0 | 0 | 0 | 0 | 0 | 0 |
| 62 | pGN2-KPC | MF156710.1 | KPC | IncFIIY | 0 | 0 | 0 | 0 | 0 | 0 | 0 | 0 | 0 | 0 | 0 | 0 | 0 | 0 | 0 |
| 63 | pGN28-KPC | MF156712.1 | KPC | IncFIIY | 0 | 0 | 0 | 0 | 0 | 0 | 0 | 0 | 0 | 0 | 0 | 0 | 0 | 0 | 0 |
| 64 | pE20-KPC | MF156709.1 | KPC | IncFIIY | 0 | 0 | 0 | 0 | 0 | 0 | 0 | 0 | 0 | 0 | 0 | 0 | 0 | 0 | 0 |
| 65 | pKP1194a | KX756453.1 | KPC | A005 | 0 | 0 | 0 | 0 | 0 | 0 | 0 | 0 | 0 | 0 | 0 | 0 | 0 | 0 | 0 |
| 66 | 15S | FJ223606.1 | KPC | A007 | 0 | 0 | 0 | 0 | 0 | 0 | 0 | 0 | 0 | 0 | 0 | 0 | 0 | 0 | 0 |
| 67 | pKPC-SMH | KT148595.1 | KPC | A007 | 0 | 0 | 0 | 0 | 0 | 0 | 0 | 0 | 0 | 0 | 0 | 0 | 0 | 0 | 0 |
| 68 | IncX-3 | KU934011.1 | KPC | parB | 0 | 0 | 0 | 0 | 0 | 0 | 0 | 0 | 0 | 0 | 0 | 0 | 0 | 0 | 0 |
| 69 | pEC4365 | KX783439.1 | KPC | A007 | 0 | 0 | 0 | 0 | 0 | 0 | 0 | 0 | 0 | 0 | 0 | 0 | 0 | 0 | 0 |
| 70 | pKP4365 | KX783440.1 | KPC | A007 | 0 | 0 | 0 | 0 | 0 | 0 | 0 | 0 | 0 | 0 | 0 | 0 | 0 | 0 | 0 |
| 71 | pCr-145 | KY659388.1 | KPC | A007 | 0 | 0 | 0 | 0 | 0 | 0 | 0 | 0 | 0 | 0 | 0 | 0 | 0 | 0 | 0 |
| 72 | pRYCKPC3.1 | NC_019151.1 | KPC | A007 | 0 | 0 | 0 | 0 | 0 | 0 | 0 | 0 | 0 | 0 | 0 | 0 | 0 | 0 | 0 |
| 73 | pHS10842 | KP125892.1 | KPC | A010 | 0 | 0 | 0 | 0 | 0 | 0 | 0 | 0 | 0 | 0 | 0 | 0 | 0 | 0 | 0 |
| 74 | pNE1280 | JQ837276.1 | KPC | A011 | 0 | 0 | 0 | 0 | 0 | 0 | 0 | 0 | 0 | 0 | 0 | 0 | 0 | 0 | 0 |
| 75 | pKPC_CAV1042-44 | CP018668.1 | KPC | HCM1 | 0 | 0 | 0 | 0 | 0 | 0 | 0 | 0 | 0 | 0 | 0 | 0 | 0 | 0 | 0 |
| 76 | pD5170990 | KX169264.1 | KPC | HCM1 043 | 0 | 0 | 0 | 0 | 0 | 0 | 0 | 0 | 0 | 0 | 0 | 0 | 0 | 0 | 0 |
| 77 | pHS062105 | KF623109.1 | KPC | HCM1 116 | 0 | 0 | 0 | 0 | 0 | 0 | 0 | 0 | 0 | 0 | 0 | 0 | 0 | 0 | 0 |
| 78 | pHS102707 | KF701335.1 | KPC | HCM1 178ac | 0 | 0 | 0 | 0 | 0 | 0 | 0 | 0 | 0 | 0 | 0 | 0 | 0 | 0 | 0 |
| 79 | pKPC_CAV1193* | CP013325.1 | KPC | HCM1 259 | 0 | 0 | 0 | 0 | 0 | 0 | 0 | 0 | 0 | 0 | 0 | 0 | 0 | 0 | 0 |
| 80 | pMNCRE78_3 | CP018432.1 | KPC | HCM1 259 | 0 | 0 | 0 | 0 | 0 | 0 | 0 | 0 | 0 | 0 | 0 | 0 | 0 | 0 | 0 |
| 81 | pKPC_CAV1344* | CP011622.1 | KPC | IncA/C | 0 | 0 | 0 | 0 | 0 | 0 | 0 | 0 | 0 | 0 | 0 | 0 | 0 | 0 | 0 |
| 82 | pKEC-dc3* | CP007732.1 | KPC | IncA/C | 0 | 0 | 0 | 0 | 0 | 0 | 0 | 0 | 0 | 0 | 0 | 0 | 0 | 0 | 0 |
| 83 | KP1768_p1 | CP025141.1 | KPC | IncA/C | 0 | 0 | 0 | 0 | 0 | 0 | 0 | 0 | 0 | 0 | 0 | 0 | 0 | 0 | 0 |
| 84 | NR5632_p1 | CP025144.1 | KPC | IncA/C | 0 | 0 | 0 | 0 | 0 | 0 | 0 | 0 | 0 | 0 | 0 | 0 | 0 | 0 | 0 |
| 85 | KP1766_p1 | CP025147.1 | KPC | IncA/C | 0 | 0 | 0 | 0 | 0 | 0 | 0 | 0 | 0 | 0 | 0 | 0 | 0 | 0 | 0 |
| 86 | pCRKP-1-KPC | KX928750.1 | KPC | IncN | 0 | 0 | 0 | 0 | 0 | 0 | 0 | 0 | 0 | 0 | 0 | 0 | 0 | 0 | 0 |
| 87 | pCRKP-5-KPC | KX928751.1 | KPC | IncN | 0 | 0 | 0 | 0 | 0 | 0 | 0 | 0 | 0 | 0 | 0 | 0 | 0 | 0 | 0 |
| 88 | pJF-WMKPCN1 | KX881941.1 | KPC | IncN | 0 | 0 | 0 | 0 | 0 | 0 | 0 | 0 | 0 | 0 | 0 | 0 | 0 | 0 | 0 |
| 89 | pKp145_11a | KX154765.1 | KPC | IncN | 0 | 0 | 0 | 0 | 0 | 0 | 0 | 0 | 0 | 0 | 0 | 0 | 0 | 0 | 0 |
| 90 | pKP148 | KX062091.1 | KPC | IncN | 0 | 0 | 0 | 0 | 0 | 0 | 0 | 0 | 0 | 0 | 0 | 0 | 0 | 0 | 0 |
| 91 | pKp196 | KX397572.1 | KPC | IncN | 0 | 0 | 0 | 0 | 0 | 0 | 0 | 0 | 0 | 0 | 0 | 0 | 0 | 0 | 0 |
| 92 | pKp314_11a | KX276209.1 | KPC | IncN | 0 | 0 | 0 | 0 | 0 | 0 | 0 | 0 | 0 | 0 | 0 | 0 | 0 | 0 | 0 |
| 93 | pKPC_FCF_3SP | NC_021660.2 | KPC | IncN | 0 | 0 | 0 | 0 | 0 | 0 | 0 | 0 | 0 | 0 | 0 | 0 | 0 | 0 | 0 |
| 94 | pKPC_FCF13_05 | NC_021664.2 | KPC | IncN | 0 | 0 | 0 | 0 | 0 | 0 | 0 | 0 | 0 | 0 | 0 | 0 | 0 | 0 | 0 |
| 95 | 12 | FJ223605.1 | KPC | IncN | 0 | 0 | 0 | 0 | 0 | 0 | 0 | 0 | 0 | 0 | 0 | 0 | 0 | 0 | 0 |
| 96 | 9 | FJ223607.1 | KPC | IncN | 0 | 0 | 0 | 0 | 0 | 0 | 0 | 0 | 0 | 0 | 0 | 0 | 0 | 0 | 0 |
| 97 | pBK31551 | JX193301.1 | KPC | IncN | 0 | 0 | 0 | 0 | 0 | 0 | 0 | 0 | 0 | 0 | 0 | 0 | 0 | 0 | 0 |
| 98 | pIncX-SHV | NC_019157.1 | KPC | IncX | 0 | 0 | 0 | 0 | 0 | 0 | 0 | 0 | 0 | 0 | 0 | 0 | 0 | 0 | 0 |
| 99 | 8-p45-IncX3 | KT362706.1 | KPC | IncX3 |  | 0 | 0 | 0 | 0 | 0 | 0 | 0 | 0 | 0 | 0 | 0 | 0 | 0 | 0 |
| 100 | pKP1433 | NC_021238.1 | KPC | korA | 0 | 0 | 0 | 0 | 0 | 0 | 0 | 0 | 0 | 0 | 0 | 0 | 0 | 0 | 0 |
| 101 | pYD626E | KJ933392.1 | KPC | korA | 0 | 0 | 0 | 0 | 0 | 0 | 0 | 0 | 0 | 0 | 0 | 0 | 0 | 0 | 0 |
| 102 | pKP13d | CP003997.1 | KPC | parB | 0 | 0 | 0 | 0 | 0 | 0 | 0 | 0 | 0 | 0 | 0 | 0 | 0 | 0 | 0 |
| 103 | pKPC_Kp01 | KX348144.1 | KPC | parB | 0 | 0 | 0 | 0 | 0 | 0 | 0 | 0 | 0 | 0 | 0 | 0 | 0 | 0 | 0 |
| 104 | pCfr-30 | KY659387.1 | KPC | parB | 0 | 0 | 0 | 0 | 0 | 0 | 0 | 0 | 0 | 0 | 0 | 0 | 0 | 0 | 0 |
| 105 | pKPC-DK05 | KR091915.1 | KPC | repN | 0 | 0 | 0 | 0 | 0 | 0 | 0 | 0 | 0 | 0 | 0 | 0 | 0 | 0 | 0 |
| 106 | pBK31567 | JX193302.1 | KPC | traJ | 0 | 0 | 0 | 0 | 0 | 0 | 0 | 0 | 0 | 0 | 0 | 0 | 0 | 0 | 0 |
| 107 | pUCLAKPC1 | KY930324.1 | KPC | trbA | 0 | 0 | 0 | 0 | 0 | 0 | 0 | 0 | 0 | 0 | 0 | 0 | 0 | 0 | 0 |
| 108 | pKPC-NY79 | NC_019161.1 | KPC | UT | 0 | 0 | 0 | 0 | 0 | 0 | 0 | 0 | 0 | 0 | 0 | 0 | 0 | 0 | 0 |
| 109 | pKpS90 | NC_019384.1 | KPC | UT | 0 | 0 | 0 | 0 | 0 | 0 | 0 | 0 | 0 | 0 | 0 | 0 | 0 | 0 | 0 |
| 110 | pKPC-NY79 | NC_019161.1 | KPC | UT | 0 | 0 | 0 | 0 | 0 | 0 | 0 | 0 | 0 | 0 | 0 | 0 | 0 | 0 | 0 |
| 111 | pDMC1097 | CP011978.1 | KPC | UT | 0 | 0 | 0 | 0 | 0 | 0 | 0 | 0 | 0 | 0 | 0 | 0 | 0 | 0 | 0 |

| 112 | pFOS18 | KJ653815.1 | KPC | UT | 0 | 0 | 0 | 0 | 0 | 0 | 0 | 0 | 0 | 0 | 0 | 0 | 0 | 0 | 0 |
| --- | --- | --- | --- | --- | --- | --- | --- | --- | --- | --- | --- | --- | --- | --- | --- | --- | --- | --- | --- |
| 113 | pKP64477d | MF150120.1 | KPC | UT | 0 | 0 | 0 | 0 | 0 | 0 | 0 | 0 | 0 | 0 | 0 | 0 | 0 | 0 | 0 |
| 114 | pKPC_CAV1042-89 | CP018669.1 | KPC | UT | 0 | 0 | 0 | 0 | 0 | 0 | 0 | 0 | 0 | 0 | 0 | 0 | 0 | 0 | 0 |
| 115 | pKPC_Kp02 | KX348145.1 | KPC | UT | 0 | 0 | 0 | 0 | 0 | 0 | 0 | 0 | 0 | 0 | 0 | 0 | 0 | 0 | 0 |
| 116 | pKPC_Kp46 | KX348146.1 | KPC | UT | 0 | 0 | 0 | 0 | 0 | 0 | 0 | 0 | 0 | 0 | 0 | 0 | 0 | 0 | 0 |
| 117 | pKPC_UVA01* | CP009465.1 | KPC | UT | 0 | 0 | 0 | 0 | 0 | 0 | 0 | 0 | 0 | 0 | 0 | 0 | 0 | 0 | 0 |
| 118 | pMNCRE53_3 | CP018436.1 | KPC | UT | 0 | 0 | 0 | 0 | 0 | 0 | 0 | 0 | 0 | 0 | 0 | 0 | 0 | 0 | 0 |
| 119 | pMNCRE69_3 | CP018426.1 | KPC | UT | 0 | 0 | 0 | 0 | 0 | 0 | 0 | 0 | 0 | 0 | 0 | 0 | 0 | 0 | 0 |
| 120 | pUCLAKPC2 | KY930325.1 | KPC | UT | 0 | 0 | 0 | 0 | 0 | 0 | 0 | 0 | 0 | 0 | 0 | 0 | 0 | 0 | 0 |
| 121 | pBK15692 | KC845573.1 | KPC | UT | 0 | 0 | 0 | 0 | 0 | 0 | 0 | 0 | 0 | 0 | 0 | 0 | 0 | 0 | 0 |
