# Supplementary material for: The type I-E CRISPR-Cas system influences the acquisition of blaKPC-IncF plasmid in Klebsiella pneumonia
Source: Emerg Microbes Infect. 2020 May 20;9(1):1011–22. doi: 10.1080/22221751.2020.1763209 (PMC7301723; doi:10.1080/22221751.2020.1763209)
Supplement: Supplemental Material [file TEMI_A_1763209_SM1594.zip › Supplementary files/supplementary data1c.docx]

Supplementary data 1c. 14 proto-spacers carried by *bla*_KPC_- positive plasmids matched for the CRISPR system in *K. pneumoniae*.

| Name of spacer | Sequence (5' to 3’) |
| --- | --- |
| Spacer1 | CAGACAGACAGCAGGCAGCAAACAGGGAAGAC |
| Spacer2 | GAGCAGGCACCCGCCGCAACGACGAAGAGCGC |
| Spacer3 | GTGGTTTGTTACCGTGTTGTGTGGCAAAAAGC |
| Spacer4 | GAACGGAGGAATATAAGAACAAAAGCCCGCAG |
| Spacer5 | TTAATACCAGGGGGCAGGTTCAGCAGGTCCCC |
| Spacer6 | CGATAACCGGGCGTTTCGACTGAACTCACCTC |
| Spacer7 | CCGCCGTTTAATCGCGGTGATGATATCCGGCA |
| Spacer8 | TCGTCTGAGTTCCGGCTTACGCCGTGCCGACA |
| Spacer9 | CCCCGTCGTCATTCGCGCATTCTGCGCACAGA |
| Spacer10 | TACTGCAGCAGGATGTCGTAGCCGATATAGTC |
| Spacer11 | GAAATAACCGTCTTCATTTCCACCCTCCCTCA |
| Spacer12 | TACTGAAACGGGTAATCAGCACAAATACCAAA |
| Spacer13 | ATTTTCAAATACTTTTCAGACCGGGCAATGTG |
| Spacer14 | CGTGATAGCGCGTTTAAGCATGTGCGCGGGGG |
