# Supplementary material for: The type I-E CRISPR-Cas system influences the acquisition of blaKPC-IncF plasmid in Klebsiella pneumonia
Source: Emerg Microbes Infect. 2020 May 20;9(1):1011–22. doi: 10.1080/22221751.2020.1763209 (PMC7301723; doi:10.1080/22221751.2020.1763209)
Supplement: Supplemental Material [file TEMI_A_1763209_SM1594.zip › Supplementary files/supplementary data1d.docx]

Supplementary data 1d. 459 clinical isolates in China.

| 459 clinical isolates | | | |
| --- | --- | --- | --- |
| ID | MLST | CRISPR-Cas | *bla* _KPC_ |
| 1 | 218 |  | - |
| 2 | 690 |  | - |
| 3 | 65 |  | - |
| 4 | 23 | I-E* | - |
| 5 | 11 |  | - |
| 6 | 23 | I-E* | - |
| 7 | 86 |  | - |
| 8 | 65 |  | - |
| 9 | 1 |  | - |
| 10 | 17 |  | - |
| 11 | 17 |  | - |
| 12 | 23 | I-E* | - |
| 13 | 29 |  | - |
| 14 | 39 | I-E | - |
| 15 | 11 |  | - |
| 16 | 20 |  | - |
| 17 | 147 |  | - |
| 18 | 65 |  | - |
| 19 | 14 | I-E* | - |
| 20 | 793 | I-E* | - |
| 21 | 14 | I-E* | - |
| 22 | 14 | I-E | - |
| 23 | 686 |  | - |
| 24 | 1938 | I-E | - |
| 25 | 1933 |  | - |
| 26 | 660 |  | - |
| 27 | 1419 |  | - |
| 28 | 101 |  | - |
| 29 | 872 | I-E | - |
| 30 | 23 | I-E* | - |
| 31 | 37 |  | - |
| 32 | 29 |  | - |
| 33 | 1934 | I-E* | - |
| 34 | 23 | I-E* | - |
| 35 | 152 | I-E* | - |

| 232 non-CG258 isolates | | | |
| --- | --- | --- | --- |
| ID | MLST | CRISPR-Cas | *bla* _KPC_ |
| 9 | 1 |  | - |
| 118 | 1 |  | - |
| 183 | 6 |  | - |
| 19 | 14 | I-E* | - |
| 21 | 14 | I-E* | - |
| 22 | 14 | I-E | - |
| 116 | 14 |  | - |
| 52 | 15 | I-E* | - |
| 68 | 15 | I-E* | - |
| 166 | 15 | I-E* | - |
| 170 | 15 | I-E* | - |
| 182 | 15 | I-E* | - |
| 348 | 15 | I-E* | + |
| 368 | 15 | I-E* | + |
| 369 | 15 | I-E* | + |
| 370 | 15 | I-E* | + |
| 374 | 15 | I-E* | + |
| 375 | 15 | I-E* | + |
| 378 | 15 | I-E* | + |
| 381 | 15 | I-E* | + |
| 382 | 15 | I-E* | + |
| 389 | 15 | I-E* | + |
| 392 | 15 | I-E* | + |
| 403 | 15 |  | + |
| 10 | 17 |  | - |
| 11 | 17 |  | - |
| 55 | 17 |  | - |
| 16 | 20 |  | - |
| 37 | 20 |  | - |
| 65 | 20 |  | - |
| 139 | 20 |  | - |
| 140 | 20 |  | - |
| 142 | 20 |  | - |
| 147 | 20 |  | - |
| 148 | 20 |  | - |

| 227 CG258 isolates | | | |
| --- | --- | --- | --- |
| ID | MLST | CRISPR-Cas | *bla* _KPC_ |
| 5 | 11 |  | - |
| 15 | 11 |  | - |
| 66 | 11 |  | - |
| 67 | 11 | I-E* | - |
| 158 | 11 | I-E* | - |
| 220 | 11 |  | + |
| 221 | 11 |  | + |
| 223 | 11 |  | + |
| 224 | 11 |  | + |
| 225 | 11 |  | + |
| 226 | 11 |  | + |
| 227 | 11 |  | + |
| 228 | 11 |  | + |
| 229 | 11 |  | + |
| 230 | 11 |  | + |
| 231 | 11 |  | + |
| 232 | 11 |  | + |
| 233 | 11 |  | + |
| 234 | 11 |  | + |
| 235 | 11 |  | + |
| 236 | 11 |  | + |
| 237 | 11 |  | + |
| 238 | 11 |  | + |
| 239 | 11 |  | + |
| 240 | 11 |  | + |
| 241 | 11 |  | + |
| 242 | 11 |  | + |
| 243 | 11 |  | + |
| 244 | 11 |  | + |
| 245 | 11 |  | + |
| 246 | 11 |  | + |
| 247 | 11 |  | + |
| 248 | 11 |  | + |
| 249 | 11 |  | + |
| 250 | 11 |  | + |

| 36 | 23 | I-E* | - |
| --- | --- | --- | --- |
| 37 | 20 |  | - |
| 38 | 29 |  | - |
| 39 | 23 | I-E* | - |
| 40 | 1938 | I-E | - |
| 41 | 494 | I-E* | - |
| 42 | 23 | I-E* | - |
| 43 | 309 |  | - |
| 44 | 309 |  | - |
| 45 | 218 |  | - |
| 46 | 690 | I-E | - |
| 47 | 626 | I-E | - |
| 48 | 592 | I-E | - |
| 49 | 461 |  | - |
| 50 | 23 | I-E* | - |
| 51 | 37 | I-E | - |
| 52 | 15 | I-E* | - |
| 53 | 29 |  | - |
| 54 | 23 | I-E* | - |
| 55 | 17 |  | - |
| 56 | 1939 |  | - |
| 57 | 494 | I-E* | - |
| 58 | 494 | I-E* | - |
| 59 | 36 |  | - |
| 60 | 1940 |  | - |
| 61 | 35 | I-E* | - |
| 62 | 39 | I-E | - |
| 63 | 261 |  | - |
| 64 | 655 |  | - |
| 65 | 20 |  | - |
| 66 | 11 |  | - |
| 67 | 11 | I-E* | - |
| 68 | 15 | I-E* | - |
| 69 | 494 | I-E* | - |
| 70 | 101 |  | - |
| 71 | 494 |  | - |
| 72 | 23 |  | - |
| 73 | 86 | I-E* | - |

| 150 | 20 |  | - |
| --- | --- | --- | --- |
| 152 | 20 |  | - |
| 153 | 20 |  | - |
| 154 | 20 |  | - |
| 155 | 20 |  | - |
| 156 | 20 |  | - |
| 168 | 20 |  | - |
| 4 | 23 | I-E* | - |
| 6 | 23 | I-E* | - |
| 12 | 23 | I-E* | - |
| 30 | 23 | I-E* | - |
| 34 | 23 | I-E* | - |
| 36 | 23 | I-E* | - |
| 39 | 23 | I-E* | - |
| 42 | 23 | I-E* | - |
| 50 | 23 | I-E* | - |
| 54 | 23 | I-E* | - |
| 72 | 23 |  | - |
| 105 | 23 | I-E* | - |
| 109 | 23 | I-E* | - |
| 113 | 23 | I-E* | - |
| 185 | 23 | I-E* | - |
| 186 | 23 | I-E* | - |
| 191 | 23 | I-E* | - |
| 193 | 23 | I-E* | - |
| 404 | 23 | I-E* | + |
| 203 | 25 |  | - |
| 13 | 29 |  | - |
| 32 | 29 |  | - |
| 38 | 29 |  | - |
| 53 | 29 |  | - |
| 92 | 29 |  | - |
| 93 | 29 |  | - |
| 97 | 29 |  | - |
| 98 | 29 |  | - |
| 125 | 29 |  | - |
| 114 | 34 | I-E | - |
| 61 | 35 | I-E* | - |

| 251 | 11 |  | + |
| --- | --- | --- | --- |
| 252 | 11 |  | + |
| 253 | 11 |  | + |
| 254 | 11 |  | + |
| 255 | 11 |  | + |
| 256 | 11 |  | + |
| 257 | 11 |  | + |
| 258 | 11 |  | + |
| 259 | 11 |  | + |
| 260 | 11 |  | + |
| 261 | 11 |  | + |
| 262 | 11 |  | + |
| 263 | 11 |  | + |
| 264 | 11 |  | + |
| 265 | 11 |  | + |
| 266 | 11 |  | + |
| 267 | 11 |  | + |
| 268 | 11 |  | + |
| 269 | 11 |  | + |
| 270 | 11 |  | + |
| 271 | 11 |  | + |
| 272 | 11 |  | + |
| 273 | 11 |  | + |
| 274 | 11 |  | + |
| 275 | 11 |  | + |
| 276 | 11 |  | + |
| 277 | 11 |  | + |
| 278 | 11 |  | + |
| 279 | 11 |  | + |
| 280 | 11 |  | + |
| 281 | 11 |  | + |
| 282 | 11 |  | + |
| 283 | 11 |  | + |
| 284 | 11 |  | + |
| 285 | 11 |  | + |
| 286 | 11 |  | + |
| 287 | 11 |  | + |
| 288 | 11 |  | + |

| 74 | 134 |  | - |
| --- | --- | --- | --- |
| 75 | 1935 |  | - |
| 76 | 36 | I-E* | - |
| 77 | 36 |  | - |
| 78 | 36 |  | - |
| 79 | 1936 |  | - |
| 80 | 101 |  | - |
| 81 | ND |  | - |
| 82 | 1937 | I-E | - |
| 83 | 1764 |  | - |
| 84 | 43 |  | - |
| 85 | 1770 |  | - |
| 86 | 39 | I-E | - |
| 87 | 188 |  | - |
| 88 | 35 | I-E* | - |
| 89 | 1031 | I-E* | - |
| 90 | 881 |  | - |
| 91 | 881 |  | - |
| 92 | 29 |  | - |
| 93 | 29 |  | - |
| 94 | 86 |  | - |
| 95 | 37 |  | - |
| 96 | 35 | I-E* | - |
| 97 | 29 |  | - |
| 98 | 29 |  | - |
| 99 | 1914 |  | - |
| 100 | 494 | I-E* | - |
| 101 | 152 |  | - |
| 102 | 1419 |  | - |
| 103 | 455 |  | - |
| 104 | 1419 |  | - |
| 105 | 23 | I-E* | - |
| 106 | 1326 |  | - |
| 107 | 896 |  | - |
| 108 | 584 |  | - |
| 109 | 23 | I-E* | - |
| 110 | 48 |  | - |
| 111 | 133 |  | - |

| 88 | 35 | I-E* | - |
| --- | --- | --- | --- |
| 96 | 35 | I-E* | - |
| 169 | 35 | I-E* | - |
| 190 | 35 | I-E* | - |
| 59 | 36 |  | - |
| 76 | 36 | I-E* | - |
| 77 | 36 |  | - |
| 78 | 36 |  | - |
| 31 | 37 |  | - |
| 51 | 37 | I-E | - |
| 95 | 37 |  | - |
| 119 | 37 |  | - |
| 14 | 39 | I-E | - |
| 62 | 39 | I-E | - |
| 86 | 39 | I-E | - |
| 84 | 43 |  | - |
| 110 | 48 |  | - |
| 112 | 48 |  | - |
| 133 | 54 |  | - |
| 135 | 54 |  | - |
| 136 | 54 |  | - |
| 137 | 54 |  | - |
| 138 | 54 |  | - |
| 144 | 54 |  | - |
| 145 | 54 |  | - |
| 146 | 54 |  | - |
| 149 | 54 |  | - |
| 151 | 54 |  | - |
| 157 | 64 |  | - |
| 3 | 65 |  | - |
| 8 | 65 |  | - |
| 18 | 65 |  | - |
| 174 | 65 |  | - |
| 197 | 65 |  | - |
| 202 | 65 |  | - |
| 218 | 65 |  | + |
| 222 | 65 |  | + |
| 178 | 76 |  | - |

| 289 | 11 |  | + |
| --- | --- | --- | --- |
| 290 | 11 |  | + |
| 291 | 11 |  | + |
| 292 | 11 |  | + |
| 293 | 11 |  | + |
| 294 | 11 |  | + |
| 295 | 11 |  | + |
| 296 | 11 |  | + |
| 297 | 11 |  | + |
| 298 | 11 |  | + |
| 299 | 11 |  | + |
| 300 | 11 |  | + |
| 301 | 11 |  | + |
| 302 | 11 |  | + |
| 303 | 11 |  | + |
| 304 | 11 |  | + |
| 305 | 11 |  | + |
| 306 | 11 |  | + |
| 307 | 11 |  | + |
| 308 | 11 |  | + |
| 309 | 11 |  | + |
| 310 | 11 |  | + |
| 311 | 11 |  | + |
| 312 | 11 |  | + |
| 313 | 11 |  | + |
| 314 | 11 |  | + |
| 315 | 11 |  | + |
| 316 | 11 |  | + |
| 317 | 11 |  | + |
| 318 | 11 |  | + |
| 319 | 11 |  | + |
| 320 | 11 |  | + |
| 321 | 11 |  | + |
| 322 | 11 |  | + |
| 323 | 11 |  | + |
| 324 | 11 |  | + |
| 325 | 11 |  | + |
| 326 | 11 |  | + |

| 112 | 48 |  | - |
| --- | --- | --- | --- |
| 113 | 23 | I-E* | - |
| 114 | 34 | I-E | - |
| 115 | 502 | I-E* | - |
| 116 | 14 |  | - |
| 117 | 420 | I-E* | - |
| 118 | 1 |  | - |
| 119 | 37 |  | - |
| 120 | 592 | I-E | - |
| 121 | 86 |  | - |
| 122 | 502 |  | - |
| 123 | 661 |  | - |
| 124 | 1783 | I-E* | - |
| 125 | 29 |  | - |
| 126 | 893 | I-E | - |
| 127 | 490 |  | - |
| 128 | 661 |  | - |
| 129 | 107 |  | - |
| 130 | 412 |  | - |
| 131 | 91 | I-E* | - |
| 132 | 785 |  | - |
| 133 | 54 |  | - |
| 134 | 290 |  | - |
| 135 | 54 |  | - |
| 136 | 54 |  | - |
| 137 | 54 |  | - |
| 138 | 54 |  | - |
| 139 | 20 |  | - |
| 140 | 20 |  | - |
| 141 | 705 |  | - |
| 142 | 20 |  | - |
| 143 | 705 |  | - |
| 144 | 54 |  | - |
| 145 | 54 |  | - |
| 146 | 54 |  | - |
| 147 | 20 |  | - |
| 148 | 20 |  | - |
| 149 | 54 |  | - |

| 184 | 81 |  | - |
| --- | --- | --- | --- |
| 342 | 81 |  | + |
| 343 | 81 |  | + |
| 345 | 81 |  | + |
| 7 | 86 |  | - |
| 73 | 86 | I-E* | - |
| 94 | 86 |  | - |
| 121 | 86 |  | - |
| 189 | 86 |  | - |
| 131 | 91 | I-E* | - |
| 28 | 101 |  | - |
| 70 | 101 |  | - |
| 80 | 101 |  | - |
| 129 | 107 |  | - |
| 194 | 111 | I-E* | - |
| 111 | 133 |  | - |
| 74 | 134 |  | - |
| 17 | 147 |  | - |
| 35 | 152 | I-E* | - |
| 101 | 152 |  | - |
| 87 | 188 |  | - |
| 1 | 218 |  | - |
| 45 | 218 |  | - |
| 171 | 254 | I-E* | - |
| 63 | 261 |  | - |
| 175 | 268 |  | - |
| 134 | 290 |  | - |
| 332 | 290 |  | + |
| 43 | 309 |  | - |
| 44 | 309 |  | - |
| 160 | 334 |  | - |
| 181 | 347 |  | - |
| 130 | 412 |  | - |
| 187 | 412 |  | - |
| 188 | 412 |  | - |
| 192 | 412 | I-E* | - |
| 196 | 412 | I-E* | - |
| 195 | 412 |  | - |

| 327 | 11 |  | + |
| --- | --- | --- | --- |
| 328 | 11 |  | + |
| 329 | 11 |  | + |
| 330 | 11 |  | + |
| 331 | 11 |  | + |
| 333 | 11 |  | + |
| 334 | 11 |  | + |
| 335 | 11 |  | + |
| 336 | 11 |  | + |
| 337 | 11 |  | + |
| 338 | 11 |  | + |
| 339 | 11 |  | + |
| 340 | 11 |  | + |
| 341 | 11 |  | + |
| 344 | 11 |  | + |
| 346 | 11 |  | + |
| 347 | 11 |  | + |
| 349 | 11 |  | + |
| 350 | 11 |  | + |
| 351 | 11 |  | + |
| 352 | 11 |  | + |
| 353 | 11 |  | + |
| 354 | 11 |  | + |
| 355 | 11 |  | + |
| 356 | 11 |  | + |
| 357 | 11 |  | + |
| 358 | 11 |  | + |
| 359 | 11 |  | + |
| 360 | 11 |  | + |
| 361 | 11 |  | + |
| 362 | 11 |  | + |
| 363 | 11 |  | + |
| 364 | 11 |  | + |
| 365 | 11 |  | + |
| 366 | 11 |  | + |
| 367 | 11 |  | + |
| 371 | 11 |  | + |
| 372 | 11 |  | + |

| 150 | 20 |  | - |
| --- | --- | --- | --- |
| 151 | 54 |  | - |
| 152 | 20 |  | - |
| 153 | 20 |  | - |
| 154 | 20 |  | - |
| 155 | 20 |  | - |
| 156 | 20 |  | - |
| 157 | 64 |  | - |
| 158 | 11 | I-E* | - |
| 159 | 2695 |  | - |
| 160 | 334 |  | - |
| 161 | 697 | I-E | - |
| 162 | 697 | I-E | - |
| 163 | 1536 |  | - |
| 164 | 1446 | I-E | - |
| 165 | 2464 |  | - |
| 166 | 15 | I-E* | - |
| 167 | 692 | I-E* | - |
| 168 | 20 |  | - |
| 169 | 35 | I-E* | - |
| 170 | 15 | I-E* | - |
| 171 | 254 | I-E* | - |
| 172 | 814 | I-E | - |
| 173 | 580 | I-E | - |
| 174 | 65 |  | - |
| 175 | 268 |  | - |
| 176 | 876 | I-E | - |
| 177 | 1265 | I-E* | - |
| 178 | 76 |  | - |
| 179 | 1653 | I-E/I-E* | - |
| 180 | 1265 | I-E* | - |
| 181 | 347 |  | - |
| 182 | 15 | I-E* | - |
| 183 | 6 |  | - |
| 184 | 81 |  | - |
| 185 | 23 | I-E* | - |
| 186 | 23 | I-E* | - |
| 187 | 412 |  | - |

| 117 | 420 | I-E* | - |
| --- | --- | --- | --- |
| 213 | 423 |  | + |
| 214 | 423 |  | + |
| 215 | 423 |  | + |
| 216 | 423 |  | + |
| 217 | 423 |  | + |
| 103 | 455 |  | - |
| 49 | 461 |  | - |
| 127 | 490 |  | - |
| 41 | 494 | I-E* | - |
| 57 | 494 | I-E* | - |
| 58 | 494 | I-E* | - |
| 69 | 494 | I-E* | - |
| 71 | 494 |  | - |
| 100 | 494 | I-E* | - |
| 115 | 502 | I-E* | - |
| 122 | 502 |  | - |
| 173 | 580 | I-E | - |
| 108 | 584 |  | - |
| 48 | 592 | I-E | - |
| 120 | 592 | I-E | - |
| 204 | 612 |  | - |
| 47 | 626 | I-E | - |
| 64 | 655 |  | - |
| 26 | 660 |  | - |
| 123 | 661 |  | - |
| 128 | 661 |  | - |
| 23 | 686 |  | - |
| 2 | 690 |  | - |
| 46 | 690 | I-E | - |
| 167 | 692 | I-E* | - |
| 161 | 697 | I-E | - |
| 162 | 697 | I-E | - |
| 141 | 705 |  | - |
| 143 | 705 |  | - |
| 132 | 785 |  | - |
| 20 | 793 | I-E* | - |
| 172 | 814 | I-E | - |

| 373 | 11 |  | + |
| --- | --- | --- | --- |
| 376 | 11 |  | + |
| 377 | 11 |  | + |
| 379 | 11 |  | + |
| 380 | 11 |  | + |
| 383 | 11 |  | + |
| 384 | 11 |  | + |
| 385 | 11 |  | + |
| 386 | 11 |  | + |
| 387 | 11 |  | + |
| 388 | 11 |  | + |
| 390 | 11 |  | + |
| 391 | 11 |  | + |
| 393 | 11 |  | + |
| 394 | 11 |  | + |
| 395 | 11 |  | + |
| 396 | 11 |  | + |
| 397 | 11 |  | + |
| 398 | 11 |  | + |
| 399 | 11 |  | + |
| 400 | 11 |  | + |
| 401 | 11 |  | + |
| 402 | 11 |  | + |
| 405 | 11 |  | + |
| 406 | 11 |  | + |
| 407 | 11 |  | + |
| 408 | 11 |  | + |
| 409 | 11 |  | + |
| 410 | 11 |  | + |
| 411 | 11 |  | + |
| 412 | 11 |  | + |
| 413 | 11 |  | + |
| 414 | 11 |  | + |
| 415 | 11 |  | + |
| 416 | 11 |  | + |
| 417 | 11 |  | + |
| 418 | 11 |  | + |
| 419 | 11 |  | + |

| 188 | 412 |  | - |
| --- | --- | --- | --- |
| 189 | 86 |  | - |
| 190 | 35 | I-E* | - |
| 191 | 23 | I-E* | - |
| 192 | 412 | I-E* | - |
| 193 | 23 | I-E* | - |
| 194 | 111 | I-E* | - |
| 195 | 412 |  | - |
| 196 | 412 | I-E* | - |
| 197 | 65 |  | - |
| 198 | 2158 |  | - |
| 199 | ND |  | - |
| 200 | 893 | I-E | - |
| 201 | 1536 |  | - |
| 202 | 65 |  | - |
| 203 | 25 |  | - |
| 204 | 612 |  | - |
| 205 | ND | I-E | - |
| 206 | ND |  | - |
| 207 | ND |  | - |
| 208 | ND |  | - |
| 209 | ND | I-E* | - |
| 210 | ND |  | - |
| 211 | ND |  | - |
| 212 | ND |  | - |
| 213 | 423 |  | + |
| 214 | 423 |  | + |
| 215 | 423 |  | + |
| 216 | 423 |  | + |
| 217 | 423 |  | + |
| 218 | 65 |  | + |
| 219 | 977 |  | + |
| 220 | 11 |  | + |
| 221 | 11 |  | + |
| 222 | 65 |  | + |
| 223 | 11 |  | + |
| 224 | 11 |  | + |
| 225 | 11 |  | + |

| 29 | 872 | I-E | - |
| --- | --- | --- | --- |
| 176 | 876 | I-E | - |
| 90 | 881 |  | - |
| 91 | 881 |  | - |
| 126 | 893 | I-E | - |
| 200 | 893 | I-E | - |
| 107 | 896 |  | - |
| 219 | 977 |  | + |
| 89 | 1031 | I-E* | - |
| 177 | 1265 | I-E* | - |
| 180 | 1265 | I-E* | - |
| 106 | 1326 |  | - |
| 27 | 1419 |  | - |
| 102 | 1419 |  | - |
| 104 | 1419 |  | - |
| 164 | 1446 | I-E | - |
| 163 | 1536 |  | - |
| 201 | 1536 |  | - |
| 179 | 1653 | I-E/I-E* | - |
| 83 | 1764 |  | - |
| 85 | 1770 |  | - |
| 124 | 1783 | I-E* | - |
| 99 | 1914 |  | - |
| 25 | 1933 |  | - |
| 33 | 1934 | I-E* | - |
| 75 | 1935 |  | - |
| 79 | 1936 |  | - |
| 82 | 1937 | I-E | - |
| 24 | 1938 | I-E | - |
| 40 | 1938 | I-E | - |
| 56 | 1939 |  | - |
| 60 | 1940 |  | - |
| 198 | 2158 |  | - |
| 165 | 2464 |  | - |
| 159 | 2695 |  | - |
| 81 | ND |  | - |
| 199 | ND |  | - |
| 205 | ND | I-E | - |

| 420 | 11 |  | + |
| --- | --- | --- | --- |
| 421 | 11 |  | + |
| 422 | 11 |  | + |
| 423 | 11 |  | + |
| 424 | 11 |  | + |
| 425 | 11 |  | + |
| 426 | 11 |  | + |
| 427 | 11 |  | + |
| 428 | 11 |  | + |
| 429 | 11 |  | + |
| 430 | 11 |  | + |
| 431 | 11 |  | + |
| 432 | 11 |  | + |
| 433 | 11 |  | + |
| 434 | 11 |  | + |
| 435 | 11 |  | + |
| 436 | 11 |  | + |
| 437 | 11 |  | + |
| 438 | 11 |  | + |
| 439 | 11 |  | + |
| 440 | 11 |  | + |
| 441 | 11 |  | + |
| 442 | 11 |  | + |
| 443 | 11 |  | + |
| 444 | 11 |  | + |
| 445 | 11 |  | + |
| 446 | 11 |  | + |
| 447 | 11 |  | + |
| 448 | 11 |  | + |
| 449 | 11 |  | + |
| 450 | 11 |  | + |
| 451 | 11 |  | + |
| 452 | 11 |  | + |
| 453 | 11 |  | + |
| 454 | 11 |  | + |
| 455 | 11 |  | + |
| 456 | 11 |  | + |
| 457 | 11 |  | + |

| 226 | 11 |  | + |
| --- | --- | --- | --- |
| 227 | 11 |  | + |
| 228 | 11 |  | + |
| 229 | 11 |  | + |
| 230 | 11 |  | + |
| 231 | 11 |  | + |
| 232 | 11 |  | + |
| 233 | 11 |  | + |
| 234 | 11 |  | + |
| 235 | 11 |  | + |
| 236 | 11 |  | + |
| 237 | 11 |  | + |
| 238 | 11 |  | + |
| 239 | 11 |  | + |
| 240 | 11 |  | + |
| 241 | 11 |  | + |
| 242 | 11 |  | + |
| 243 | 11 |  | + |
| 244 | 11 |  | + |
| 245 | 11 |  | + |
| 246 | 11 |  | + |
| 247 | 11 |  | + |
| 248 | 11 |  | + |
| 249 | 11 |  | + |
| 250 | 11 |  | + |
| 251 | 11 |  | + |
| 252 | 11 |  | + |
| 253 | 11 |  | + |
| 254 | 11 |  | + |
| 255 | 11 |  | + |
| 256 | 11 |  | + |
| 257 | 11 |  | + |
| 258 | 11 |  | + |
| 259 | 11 |  | + |
| 260 | 11 |  | + |
| 261 | 11 |  | + |
| 262 | 11 |  | + |
| 263 | 11 |  | + |

| 206 | ND |  | - |
| --- | --- | --- | --- |
| 207 | ND |  | - |
| 208 | ND |  | - |
| 209 | ND | I-E* | - |
| 210 | ND |  | - |
| 211 | ND |  | - |
| 212 | ND |  | - |

| 458 | 11 |  | + |
| --- | --- | --- | --- |
| 459 | 11 |  | + |

| 264 | 11 |  | + |
| --- | --- | --- | --- |
| 265 | 11 |  | + |
| 266 | 11 |  | + |
| 267 | 11 |  | + |
| 268 | 11 |  | + |
| 269 | 11 |  | + |
| 270 | 11 |  | + |
| 271 | 11 |  | + |
| 272 | 11 |  | + |
| 273 | 11 |  | + |
| 274 | 11 |  | + |
| 275 | 11 |  | + |
| 276 | 11 |  | + |
| 277 | 11 |  | + |
| 278 | 11 |  | + |
| 279 | 11 |  | + |
| 280 | 11 |  | + |
| 281 | 11 |  | + |
| 282 | 11 |  | + |
| 283 | 11 |  | + |
| 284 | 11 |  | + |
| 285 | 11 |  | + |
| 286 | 11 |  | + |
| 287 | 11 |  | + |
| 288 | 11 |  | + |
| 289 | 11 |  | + |
| 290 | 11 |  | + |
| 291 | 11 |  | + |
| 292 | 11 |  | + |
| 293 | 11 |  | + |
| 294 | 11 |  | + |
| 295 | 11 |  | + |
| 296 | 11 |  | + |
| 297 | 11 |  | + |
| 298 | 11 |  | + |
| 299 | 11 |  | + |
| 300 | 11 |  | + |
| 301 | 11 |  | + |

| 302 | 11 |  | + |
| --- | --- | --- | --- |
| 303 | 11 |  | + |
| 304 | 11 |  | + |
| 305 | 11 |  | + |
| 306 | 11 |  | + |
| 307 | 11 |  | + |
| 308 | 11 |  | + |
| 309 | 11 |  | + |
| 310 | 11 |  | + |
| 311 | 11 |  | + |
| 312 | 11 |  | + |
| 313 | 11 |  | + |
| 314 | 11 |  | + |
| 315 | 11 |  | + |
| 316 | 11 |  | + |
| 317 | 11 |  | + |
| 318 | 11 |  | + |
| 319 | 11 |  | + |
| 320 | 11 |  | + |
| 321 | 11 |  | + |
| 322 | 11 |  | + |
| 323 | 11 |  | + |
| 324 | 11 |  | + |
| 325 | 11 |  | + |
| 326 | 11 |  | + |
| 327 | 11 |  | + |
| 328 | 11 |  | + |
| 329 | 11 |  | + |
| 330 | 11 |  | + |
| 331 | 11 |  | + |
| 332 | 290 |  | + |
| 333 | 11 |  | + |
| 334 | 11 |  | + |
| 335 | 11 |  | + |
| 336 | 11 |  | + |
| 337 | 11 |  | + |
| 338 | 11 |  | + |
| 339 | 11 |  | + |

| 340 | 11 |  | + |
| --- | --- | --- | --- |
| 341 | 11 |  | + |
| 342 | 81 |  | + |
| 343 | 81 |  | + |
| 344 | 11 |  | + |
| 345 | 81 |  | + |
| 346 | 11 |  | + |
| 347 | 11 |  | + |
| 348 | 15 | I-E* | + |
| 349 | 11 |  | + |
| 350 | 11 |  | + |
| 351 | 11 |  | + |
| 352 | 11 |  | + |
| 353 | 11 |  | + |
| 354 | 11 |  | + |
| 355 | 11 |  | + |
| 356 | 11 |  | + |
| 357 | 11 |  | + |
| 358 | 11 |  | + |
| 359 | 11 |  | + |
| 360 | 11 |  | + |
| 361 | 11 |  | + |
| 362 | 11 |  | + |
| 363 | 11 |  | + |
| 364 | 11 |  | + |
| 365 | 11 |  | + |
| 366 | 11 |  | + |
| 367 | 11 |  | + |
| 368 | 15 | I-E* | + |
| 369 | 15 | I-E* | + |
| 370 | 15 | I-E* | + |
| 371 | 11 |  | + |
| 372 | 11 |  | + |
| 373 | 11 |  | + |
| 374 | 15 | I-E* | + |
| 375 | 15 | I-E* | + |
| 376 | 11 |  | + |
| 377 | 11 |  | + |

| 378 | 15 | I-E* | + |
| --- | --- | --- | --- |
| 379 | 11 |  | + |
| 380 | 11 |  | + |
| 381 | 15 | I-E* | + |
| 382 | 15 | I-E* | + |
| 383 | 11 |  | + |
| 384 | 11 |  | + |
| 385 | 11 |  | + |
| 386 | 11 |  | + |
| 387 | 11 |  | + |
| 388 | 11 |  | + |
| 389 | 15 | I-E* | + |
| 390 | 11 |  | + |
| 391 | 11 |  | + |
| 392 | 15 | I-E* | + |
| 393 | 11 |  | + |
| 394 | 11 |  | + |
| 395 | 11 |  | + |
| 396 | 11 |  | + |
| 397 | 11 |  | + |
| 398 | 11 |  | + |
| 399 | 11 |  | + |
| 400 | 11 |  | + |
| 401 | 11 |  | + |
| 402 | 11 |  | + |
| 403 | 15 |  | + |
| 404 | 23 | I-E* | + |
| 405 | 11 |  | + |
| 406 | 11 |  | + |
| 407 | 11 |  | + |
| 408 | 11 |  | + |
| 409 | 11 |  | + |
| 410 | 11 |  | + |
| 411 | 11 |  | + |
| 412 | 11 |  | + |
| 413 | 11 |  | + |
| 414 | 11 |  | + |
| 415 | 11 |  | + |

| 416 | 11 |  | + |
| --- | --- | --- | --- |
| 417 | 11 |  | + |
| 418 | 11 |  | + |
| 419 | 11 |  | + |
| 420 | 11 |  | + |
| 421 | 11 |  | + |
| 422 | 11 |  | + |
| 423 | 11 |  | + |
| 424 | 11 |  | + |
| 425 | 11 |  | + |
| 426 | 11 |  | + |
| 427 | 11 |  | + |
| 428 | 11 |  | + |
| 429 | 11 |  | + |
| 430 | 11 |  | + |
| 431 | 11 |  | + |
| 432 | 11 |  | + |
| 433 | 11 |  | + |
| 434 | 11 |  | + |
| 435 | 11 |  | + |
| 436 | 11 |  | + |
| 437 | 11 |  | + |
| 438 | 11 |  | + |
| 439 | 11 |  | + |
| 440 | 11 |  | + |
| 441 | 11 |  | + |
| 442 | 11 |  | + |
| 443 | 11 |  | + |
| 444 | 11 |  | + |
| 445 | 11 |  | + |
| 446 | 11 |  | + |
| 447 | 11 |  | + |
| 448 | 11 |  | + |
| 449 | 11 |  | + |
| 450 | 11 |  | + |
| 451 | 11 |  | + |
| 452 | 11 |  | + |
| 453 | 11 |  | + |

| 454 | 11 |  | + |
| --- | --- | --- | --- |
| 455 | 11 |  | + |
| 456 | 11 |  | + |
| 457 | 11 |  | + |
| 458 | 11 |  | + |
| 459 | 11 |  | + |
