# Supplementary material for: The type I-E CRISPR-Cas system influences the acquisition of blaKPC-IncF plasmid in Klebsiella pneumonia
Source: Emerg Microbes Infect. 2020 May 20;9(1):1011–22. doi: 10.1080/22221751.2020.1763209 (PMC7301723; doi:10.1080/22221751.2020.1763209)
Supplement: Supplemental Material [file TEMI_A_1763209_SM1594.zip › Supplementary files/Supplementary material legends.docx]

**Supplemental material legends**

**Fig S1. Schematics of identified CRISPR-cas systems in *K. pneumoniae.***

**Fig S2 121 *bla*_KPC_- positive plasmids in *K. pneumoniae.***

**Fig S3. A total of 459 *K. pneumoniae* clinical isolates collected from six provinces of China with different carbapenem resistance rates.**

**Fig S4 (A) Characteristics of plasmid with different proto-spacers and PAMs. (B) Construction of *Escherichia coli* BW25113 without and with the CRISPR-Cas system mutant strain**

**Table S1. Strains and plasmids used in this study**

**Table S2. Oligonucleotides for cloning or PCR**

**Table S3. MICs for clones isolated in the plasmid stable assay.**

**Table S4. Proto-Spacer sequences matching KP8 CRISPR**

**Supplementary data1**

**Supplementary data 1a. 203 completely sequenced *K. pneumoniae* strains used in this study.**

**Supplementary data 1b. 121 *bla*_KPC_- positive plasmids in *K. pneumoniae* used in this study.**

**Supplementary data 1c. 14 proto-spacers carried by *bla*_KPC_- positive plasmids matched for the CRISPR system in *K. pneumoniae*.**

**Supplementary data 1d. 459 clinical isolates in China.**

**
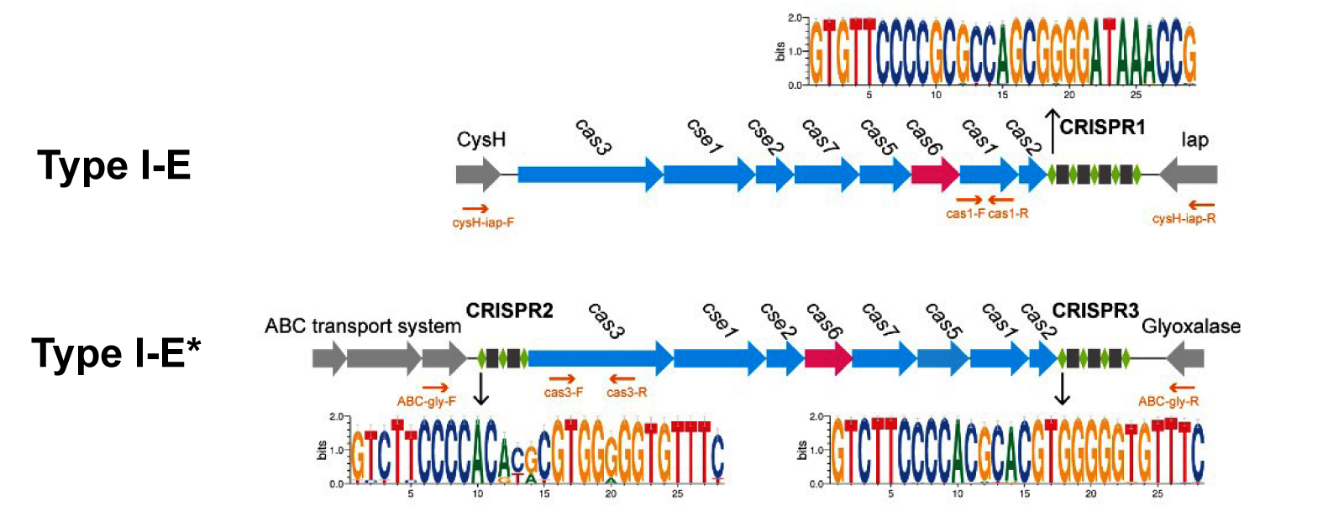
**

**Fig S1. Schematics of identified CRISPR-cas systems in *K. pneumoniae.***

Genes are depicted as arrows in different colors. The green diamonds and dark grey rectangles indicate direct repeats (DRs) and spacers, respectively. Numbers and sizes of DRs and spacers are not to scale. The consensus sequences of the repeats were generated in WebLogo. The primers for CRISPR-Cas screen are shown as small and orange arrows.


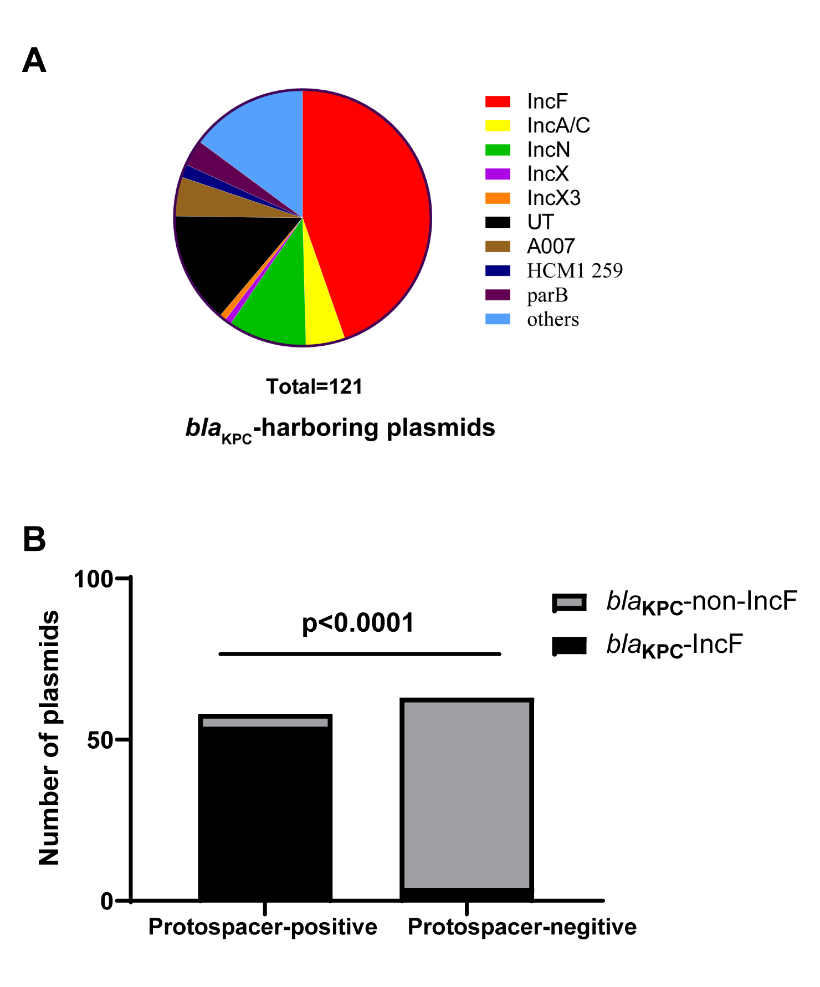


**Fig S2 121 *bla*_KPC_- positive plasmids in *K. pneumoniae.*** (A) The categories of 121 *bla*_KPC_- positive plasmids. (B) Plasmid incompatibility type (IncF and non-IncF) of *K. pneumoniae* *bla*_KPC_- positive plasmids in protospacer-positive and negative groups.

**
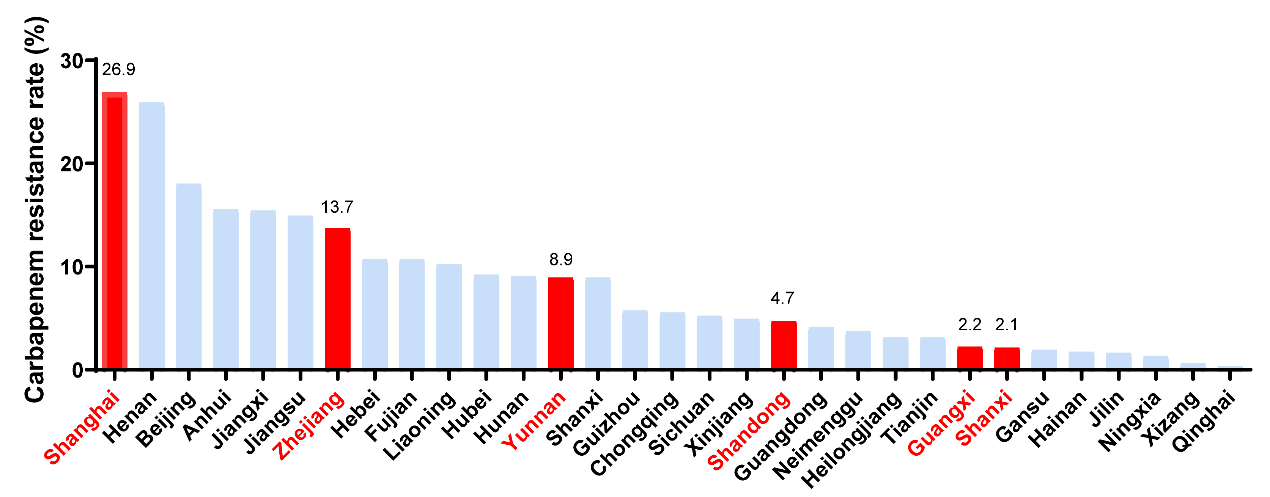
**

**Fig S3. A total of 459 *K. pneumoniae* clinical isolates collected from six provinces of China with different carbapenem resistance rates.**

Carbapenem resistance rates of *K. pneumoniae* among different provinces of China in 2017 was taken from data of China Antimicrobial Resistance Surveillance System (CARSS)*. The six red bars characterize the six provinces, from which our 459 strains collected and the resistance rate in these provinces typically represent high, medium and low levels. The variance distribution of resistance levels in the six provinces ensures the universality of our research results.

* <http://www.carss.cn/Report/Details/552>


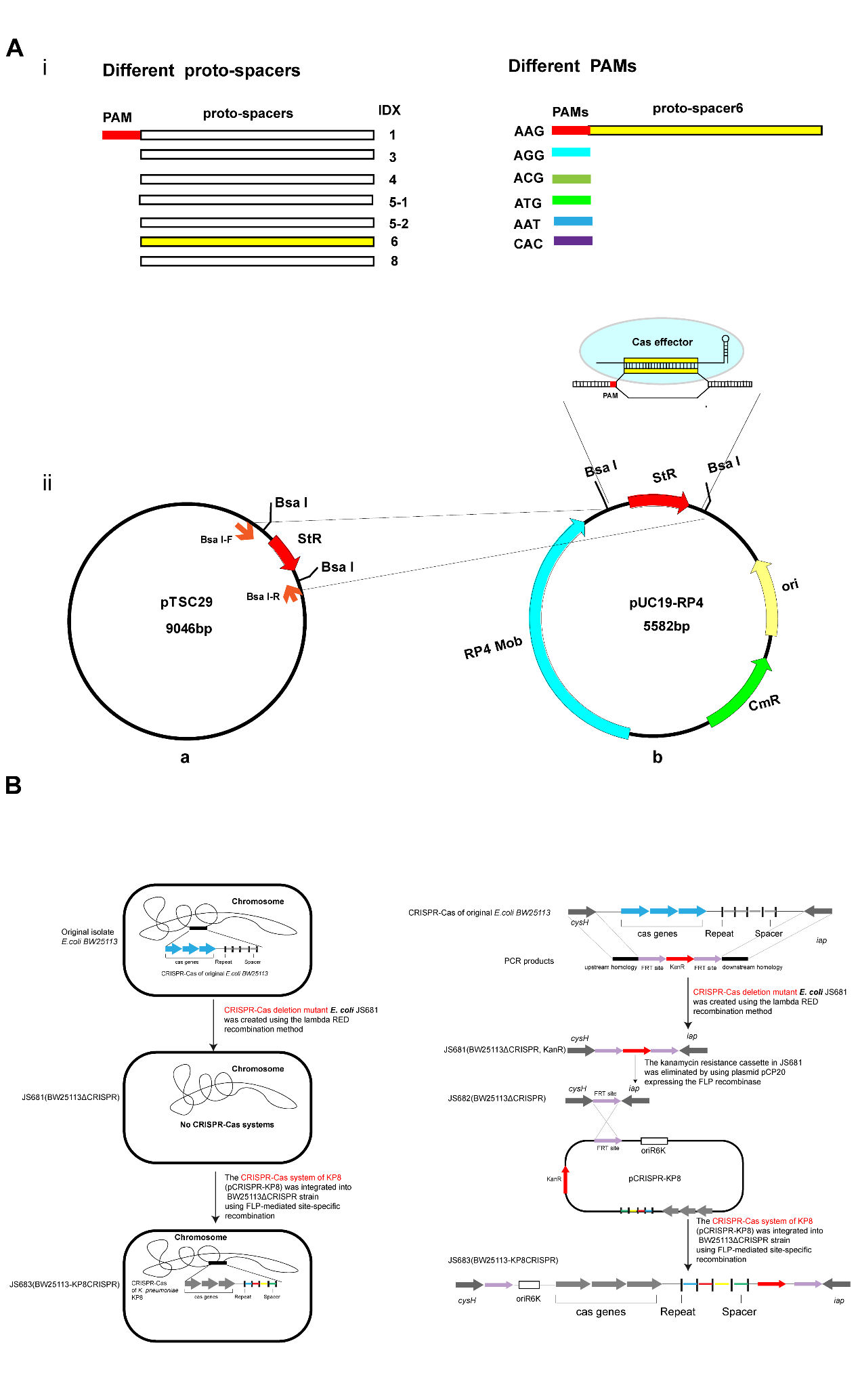


**Fig S4 (A) Characteristics of plasmid with different proto-spacers and PAMs.** The plasmid pTSC29 was used as a template (primers are shown as small and orange arrows) for amplifying the *Bas I* restriction site, in which the proto-spacers (i)were inserted. The RP4 mob (ii) cloned from the pJTOOL-3 functioned for plasmid mobilization. **(B) Construction of *Escherichia coli* BW25113 without and with the CRISPR-Cas system mutant strain**

**Table S1. Strains and plasmids used in this study**

| **Strains/plasmids** | **Relevant characteristics** | **Source or reference** |
| --- | --- | --- |
| ***K. pneumoniae*** |  |  |
| **KP8** | *K. pneumoniae* ST458(non-CC258) clinical isolates containing CRISPR-Cas system (containing spacer1, 3, 4, 5, 6 and 8). | **CP025636.1** |
| JS686 | KP8 derivative (CAM^R^) for allelic replacement of *cas3* protein with chloramphenicol resistance cassette flanked by FRT sites | This study |
| JS687 | KP8-ΔCas3 derivative, eliminates chloramphenicol resistance cassette by using FLP recombinase and contains an FRT site only | This study |
|  |  |  |
| ***E. coli*** |  |  |
| BW25113 | DE (araD-araB)567 lacZ4787(del)(::rrnB-3) lacIp-4000(lacI$^Q$) LAM- rph-1 DE(rhaD-rhaB)568 rrnB-3 hsdR514 | 23 |
| DH5αpir | endA1 hsdR17 glnV44 (= supE44) thi-1 recA1 gyrA96 relA1 φ80dlacΔ(lacZ)M15 Δ(lacZYA-argF) U169 zdg-232::Tn10 uidA::pir+ | Lab stock |
| S17-1 λpir | TpR SmR *recA, thi, pro, hsd*R-M+RP4: 2-Tc: Mu: KmR Tn*7* λ*pir* | 25 |
| MG1655 | F- lambda- ilvG- rfb-50 rph-1 | Lab stock |
| JS681 | BW25113 derivative (KAN^R^) for allelic replacement of E*. coli* CRISPR-Cas system with kanamycin resistance cassette flanked by FRT sites | This study |
| JS682 | BW25113-ΔCRISPR derivative, eliminates kanamycin resistance cassette by using FLP recombinase and contains an FRT site only | This study |
| JS683 | BW25113-ΔCRISPR: FRT derivative (KAN^R^), contains CRISPR-Cas of KP8 integrated in the FRT site | This study |
| JS531 | DH5α contains plasmid p187-2, an IncFII conjugative plasmid harbouring *bla*KPC and matches for spacer1, 3, 4, 5 and 6 recovered in K. pneumoniae. Accession number of p187-2: CP025468.1 | This study |
|  |  |  |
| **Plasmids**  **p187-2** | an IncFII conjugative plasmid harbouring *bla*_KPC_ and matches for spacer1, 3, 4, 5 and 6 recovered in *K. pneumoniae* | **CP025468.1** |
| pKD4 | AMP^R^ KAN^R^, kanamycin cassette flanked by FRT sites template, pir-dependent | 23 |
| pKD3 | AMP^R^ CAM^R^, chloramphenicol cassette flanked by FRT sites template, pir-dependent | 23 |
| pKD46 | AMP^R^, λ Red recombinase expression, 30°C | 23 |
| pKOBEG | Apr^R^, λ Red recombinase expression, 30°C | Lab stock |
| pCP20 | AMP^R^ CAM^R^, FLP recombinase expression, 30°C | 25 |
| pCP20-KAN | AMP^R^ KAN^R^, FLP recombinase expression, 30°C | This study |
| pUC19 | AMP^R^, ori pMB1 | Lab stock |
| pUC19-CAM | CAM^R^, ori pMB1 | This study |
| pUC19-Apr | Apr^R^, ori pMB1 | This study |
| pJTOOL-3 | CAM^R^, RP4-specific Mob site template | 25 |
| PTSC29 | STR^R^ CAM^R^, *Bsa I* restriction site template | Lab stock |
| pCRISPR-KP8 | KAN^R^, pKD4 derivative containing CRISPR-Cas system of KP8 and one FRT site, pir-dependent | This study |
| pUC-RP4 | CAM^R^, ori pMB1, pUC19 derivative, inserts *Bsa I* restriction site and RP4-specific Mob site by In-Fusion Cloning | This study |
|  |  |  |

**Table S2. Oligonucleotides for cloning or PCR**

| **Name** | **Sequence^a^** |  |
| --- | --- | --- |
| **For CRISPR-Cas screening** |  |  |
| cysH-iap-F | CGGTTCTTCGGGCTTAAACG |  |
| cysH-iap-R | CTGCTGCAATGACGCCAG |  |
| ABC-gly-F | TGTTCGCCGCTGAGTTTATG |  |
| ABC-gly-R | TACCACGCCAGTTACTACGC |  |
| cas1-F | CTTTTGGCACGACGGAATCA |  |
| cas1-R | TGGCGCTGGATGATGATTTG |  |
| cas3-F | GTCCCGACTAAAATGCGTCC |  |
| cas3-R | CGTTGATGGCGGTGATGAAT |  |
| **For *bla*_KPC_ Screening** |  |  |
| *bla*_KPC_-F | TCGCTAAACTCGAACAGG |  |
| *bla*_KPC_-R | TTACTGCCCGTTGACGCCCAATCC |  |
| **For constructing PUC-protospacer** |  |  |
| PUC-bsaI-F | GTTGGCGGGTGTCGGGGCGCAGCCA CTTGCCACCAGTGATGCGG |  |
| PUC-bsaI-R | TGCAGGTCGACTCTAGAGGATCCCC ATCCGTTTCCACGGTGTGC |  |
| RP4-F | GCCAGTGAATTCGAGCTCGGTACCC GATCCAGCCGACCAGGCTTT |  |
| RP4-R | TGGCTGCGCCCCGACACCCG |  |
| proto-spacer1-F | TTCG AAGCAGACAGACAGCAGGCAGCAAACAGGGAAGACGCGGA | |
| proto-spacer1-R | CAAG TCCGCGTCTTCCCTGTTTGCTGCCTGCTGTCTGTCTGCTT |  |
| proto-spacer3-F | TTCG AAGGTGGTTTGTTACCGTGTTGTGTGGCAAAAAGCAGAAA | |
| proto-spacer3-R | CAAG TTTCTGCTTTTTGCCACACAACACGGTAACAAACCACCTT | |
| proto-spacer4-F | TTCG AAGGAACGGAGGAATATAAGAACAAAAGCCCGCAGAGAAA | |
| proto-spacer4-R | CAAG TTTCTCTGCGGGCTTTTGTTCTTATATTCCTCCGTTCCTT |  |
| proto-spacer5-1-F | TTCG GAAAGTTATATCCAGGGGGCAGGTTCAGCAGGTCCCCGCACA | |
| proto-spacer5-1-R | CAAG TGTGCGGGGACCTGCTGAACCTGCCCCCTGGATATAACTTTC | |
| proto-spacer5-2-F | TTCG AAGTTAATACCAGGGGGCAGGTTCAGCAGGTCCCCGCA |  |
| proto-spacer5-2-R | CAAG TGCGGGGACCTGCTGAACCTGCCCCCTGGTATTAACTT |  |
| proto-spacer6-F | TTCG AAGCGATAACAGGGCGTTTCGACTGAACTCACCTCCCCCT | |
| proto-spacer6-R | CAAG AGGGGGAGGTGAGTTCAGTCGAAACGCCCTGTTATCGCTT | |
| proto-spacer8-F | TTCG AAGTCGTCTGAGTTCCGGCTTACGCCGTGCCGACACGA |  |
| proto-spacer8-R | CAAG TCGTGTCGGCACGGCGTAAGCCGGAACTCAGACGACTT |  |
| none-proto-spacer6-F | TTCGCGATAACAGGGCGTTTCGACTGAACTCACCTC |  |
| none-proto-spacer6-R | CAAG GAGGTGAGTTCAGTCGAAACGCCCTGTTATCG |  |
| AAT-proto-spacer6-F | TTCGAATCGATAACAGGGCGTTTCGACTGAACTCACCTC |  |
| AAT-proto-spacer6-R | CAAG GAGGTGAGTTCAGTCGAAACGCCCTGTTATCGATT |  |
| AGG-proto-spacer6-F | TTCGAGGCGATAACAGGGCGTTTCGACTGAACTCACCTC |  |
| AGG-proto-spacer6-R | CAAG GAGGTGAGTTCAGTCGAAACGCCCTGTTATCGCCT |  |
| ATG-proto-spacer6-F | TTCGATGCGATAACAGGGCGTTTCGACTGAACTCACCTC |  |
| ATG-proto-spacer6-R | CAAG GAGGTGAGTTCAGTCGAAACGCCCTGTTATCGCAT |  |
| ACG-proto-spacer6-F | TTCGACGCGATAACAGGGCGTTTCGACTGAACTCACCTC |  |
| ACG-proto-spacer6-R | CAAG GAGGTGAGTTCAGTCGAAACGCCCTGTTATCGCGT |  |
| CAC-proto-spacer6-F | TTCGCACCGATAACAGGGCGTTTCGACTGAACTCACCTC |  |
| CAC-proto-spacer6-R | CAAG GAGGTGAGTTCAGTCGAAACGCCCTGTTATCGGTG |  |
| **For constructing PUC-two-protospacer** |  |  |
| Xba I -HindIII-proto-spacer5-1-F | CTAG GAAGGTTATATCCAGGGGGCAGGTTCAGCAGGTCCCCGCACA | |
| Xba I -HindIII-proto-spacer5-1-R | AGCT TGTGCGGGGACCTGCTGAACCTGCCCCCTGGATATAACCTTC | |
| Xba I -HindIII-proto-spacer4-F | CTAG GGAAGGAACGGAGGAATATAAGAACAAAAGCCCGCAGAGAAA | |
| Xba I -HindIII-proto-spacer4-R | AGCT TTTCTCTGCGGGCTTTTGTTCTTATATTCCTCCGTTCCTTCC |  |
| **For constructing KP8 Cas3-deletion** |  |  |
| Upstream of Cas3-F | GCGCGGTTGTCTCCTCTATT |  |
| Upstream of Cas3-R | GAAGCAGCTCCAGCCTACAC TGCGGCGACATCAAGGGAAT |  |
| Downstream of Cas3-F | GGACCATGGCTAATTCCCAT GCAGCTAACTACATGAATAC |  |
| Downstream of Cas3-R | CGGGCCAAACTGAAAGGCAG |  |
| Cam-FRT-F | GTGTAGGCTGGAGCTGCTTC |  |
| Cam-FRT-R | ATGGGAATTAGCCATGGTCC |  |
| **For constructing *E. coli* BW25113 CRISPR-deletion** |  |  |
| upstream of CRISPR-F | GGAATGGATGATAACGCCGC |  |
| upstream of CRISPR-R | GAAGCAGCTCCAGCCTACACTCATGCCAGCTATTTCCCGC |  |
| Kan with FRT-F | GTGTAGGCTGGAGCTGCTTC |  |
| Kan with FRT-R | CCATATGAATATCCTCCTT |  |
| downstream of CRISPR-F | AAGGAGGATATTCATATGGCAGCACCGGTAAATTGGCAC |  |
| downstream of CRISPR-R | TCGATCTAAACGCCCTGAACG |  |
| **For constructing pCRISPR-KP8** |  |  |
| PKD4-F | AAATATCGCTACCTGCCGCTCTAGCGATAT ACTGGGCTATCTGGACAAGG | |
| PKD4-R | GGGATTAAATAATAGAGGAGACAACCGCGC GCAAGATCCGCAGTTCAACC |  |
| KP8-CRISPR1-F | ATATCGCTAGAGCGGCAGG |  |
| KP8-CRISPR1-R | GAGGTACTTCGCCAGGCTTA |  |
| KP8-CRISPR2-F | GCTGTCGCAGAGAAAGCCTC |  |
| KP8-CRISPR2-R | GCGCGGTTGTCTCCTCTATT |  |

^a^ underlines sequences were the overlaps used for SOE-PCR or plasmid construction; the red bases were the PAM seque

**Table S3. MICs for clones isolated in the plasmid stable assay.**

| Strains | Passage Number | MIC (mg/L) | |  |
| --- | --- | --- | --- | --- |
|  |  | Imipenem | Chloramphenicol |  |
| ^1^p187-2-JS683 | 10 | ≤0.25 | —— |  |
| p187-2-JS681 | 10 | ≥16 | —— |  |
|  |  |  |  |  |
| ^2^pUC-proto-spacer6-KP8 | 3 | —— | 8 |  |
| pUC-proto-spacer6-JS687(KP8ΔCas3) | 3 | —— | ＞128 |  |
| pUC-Empty-KP8 | 3 | —— | ＞128 |  |
| pUC-Empty-JS687 | 3 | —— | ＞128 |  |
|  |  |  |  |  |
| pUC-proto-spacer6-JS683 | 6 | —— | 8 |  |
| pUC-proto-spacer6-JS681 | 6 | —— | ＞128 |  |
| pUC-Empty-JS683 | 6 | —— | ＞128 |  |
| pUC Empty-JS681 | 6 | —— | ＞128 |  |
|  |  |  |  |  |
| Control |  |  |  |  |
| JS683 | —— | ≤0.25 | 8 |  |
| JS681 | —— | ≤0.25 | 8 |  |
| KP8 | —— | —— | 8 |  |
| JS687 | —— | —— | 8 |  |

Note

^1^these results indicated the imipenem sensitivity in both two strains (JS683 and JS681) after 10 times passages in LB broth, which consistent with the Fig 3A

^2^these results indicated the chloramphenicol sensitivity in *K. pneumonia* (KP8 and JS687) after 3 times passages (Fig 3Bi) and corresponding MICs in *E. coli* (JS683 and JS681) after 6 times passages (Fig.3Bii)

**Table S4. Proto-Spacer sequences matching KP8 CRISPR**

| Name of proto-spacers | Sequence (5' to 3’) ^a^ | Plasmid | Accession number | GC Content |
| --- | --- | --- | --- | --- |
| proto-spacer1 | CAGACAGACAGCAGGCAGCAAACAGGGAAGAC | p187-2 | CP025468.1 | 56% |
| proto-spacer3 | GTGGTTTGTTACCGTGTTGTGTGGCAAAAAGC | p187-2 | CP025468.1 | 47% |
| protospacer4 | GAACGGAGGAATATAAGAACAAAAGCCCGCAG | p187-2 | CP025468.1 | 47% |
| proto-spacer5-1 | TT**ATAT**CCAGGGGGCAGGTTCAGCAGGTCCCC | p187-2 | CP025468.1 | 59% |
| proto-spacer5-2 | TT**AATA**CCAGGGGGCAGGTTCAGCAGGTCCCC | p0716-KPC | KY270849.1 | 59% |
| proto-spacer6 | CGATAACAGGGCGTTTCGACTGAACTCACCTC | p187-2 | CP025468.1 | 56% |
| proto-spacer8 | TCGTCTGAGTTCCGGCTTACGCCGTGCCGACA | p12139-KPC-2 | MF168403.1 | 63% |

^a^ red bases represent the mutations.
