# Supplementary material for: The type I-E CRISPR-Cas system influences the acquisition of blaKPC-IncF plasmid in Klebsiella pneumonia
Source: Emerg Microbes Infect. 2020 May 20;9(1):1011–22. doi: 10.1080/22221751.2020.1763209 (PMC7301723; doi:10.1080/22221751.2020.1763209)
Supplement: Supplemental Material [file TEMI_A_1763209_SM1594.zip › Supplementary files/Table S2.docx]

**Table S2. Oligonucleotides for cloning or PCR**

| **Name** | **Sequence^a^** |  |
| --- | --- | --- |
| **For CRISPR-Cas screening** |  |  |
| cysH-iap-F | CGGTTCTTCGGGCTTAAACG |  |
| cysH-iap-R | CTGCTGCAATGACGCCAG |  |
| ABC-gly-F | TGTTCGCCGCTGAGTTTATG |  |
| ABC-gly-R | TACCACGCCAGTTACTACGC |  |
| cas1-F | CTTTTGGCACGACGGAATCA |  |
| cas1-R | TGGCGCTGGATGATGATTTG |  |
| cas3-F | GTCCCGACTAAAATGCGTCC |  |
| cas3-R | CGTTGATGGCGGTGATGAAT |  |
| **For *bla*_KPC_ Screening** |  |  |
| *bla*_KPC_-F | TCGCTAAACTCGAACAGG |  |
| *bla*_KPC_-R | TTACTGCCCGTTGACGCCCAATCC |  |
| **For constructing PUC-protospacer** |  |  |
| PUC-bsaI-F | GTTGGCGGGTGTCGGGGCGCAGCCA CTTGCCACCAGTGATGCGG |  |
| PUC-bsaI-R | TGCAGGTCGACTCTAGAGGATCCCC ATCCGTTTCCACGGTGTGC |  |
| RP4-F | GCCAGTGAATTCGAGCTCGGTACCC GATCCAGCCGACCAGGCTTT |  |
| RP4-R | TGGCTGCGCCCCGACACCCG |  |
| proto-spacer1-F | TTCG AAGCAGACAGACAGCAGGCAGCAAACAGGGAAGACGCGGA | |
| proto-spacer1-R | CAAG TCCGCGTCTTCCCTGTTTGCTGCCTGCTGTCTGTCTGCTT |  |
| proto-spacer3-F | TTCG AAGGTGGTTTGTTACCGTGTTGTGTGGCAAAAAGCAGAAA | |
| proto-spacer3-R | CAAG TTTCTGCTTTTTGCCACACAACACGGTAACAAACCACCTT | |
| proto-spacer4-F | TTCG AAGGAACGGAGGAATATAAGAACAAAAGCCCGCAGAGAAA | |
| proto-spacer4-R | CAAG TTTCTCTGCGGGCTTTTGTTCTTATATTCCTCCGTTCCTT |  |
| proto-spacer5-1-F | TTCG GAAAGTTATATCCAGGGGGCAGGTTCAGCAGGTCCCCGCACA | |
| proto-spacer5-1-R | CAAG TGTGCGGGGACCTGCTGAACCTGCCCCCTGGATATAACTTTC | |
| proto-spacer5-2-F | TTCG AAGTTAATACCAGGGGGCAGGTTCAGCAGGTCCCCGCA |  |
| proto-spacer5-2-R | CAAG TGCGGGGACCTGCTGAACCTGCCCCCTGGTATTAACTT |  |
| proto-spacer6-F | TTCG AAGCGATAACAGGGCGTTTCGACTGAACTCACCTCCCCCT | |
| proto-spacer6-R | CAAG AGGGGGAGGTGAGTTCAGTCGAAACGCCCTGTTATCGCTT | |
| proto-spacer8-F | TTCG AAGTCGTCTGAGTTCCGGCTTACGCCGTGCCGACACGA |  |
| proto-spacer8-R | CAAG TCGTGTCGGCACGGCGTAAGCCGGAACTCAGACGACTT |  |
| none-proto-spacer6-F | TTCGCGATAACAGGGCGTTTCGACTGAACTCACCTC |  |
| none-proto-spacer6-R | CAAG GAGGTGAGTTCAGTCGAAACGCCCTGTTATCG |  |
| AAT-proto-spacer6-F | TTCGAATCGATAACAGGGCGTTTCGACTGAACTCACCTC |  |
| AAT-proto-spacer6-R | CAAG GAGGTGAGTTCAGTCGAAACGCCCTGTTATCGATT |  |
| AGG-proto-spacer6-F | TTCGAGGCGATAACAGGGCGTTTCGACTGAACTCACCTC |  |
| AGG-proto-spacer6-R | CAAG GAGGTGAGTTCAGTCGAAACGCCCTGTTATCGCCT |  |
| ATG-proto-spacer6-F | TTCGATGCGATAACAGGGCGTTTCGACTGAACTCACCTC |  |
| ATG-proto-spacer6-R | CAAG GAGGTGAGTTCAGTCGAAACGCCCTGTTATCGCAT |  |
| ACG-proto-spacer6-F | TTCGACGCGATAACAGGGCGTTTCGACTGAACTCACCTC |  |
| ACG-proto-spacer6-R | CAAG GAGGTGAGTTCAGTCGAAACGCCCTGTTATCGCGT |  |
| CAC-proto-spacer6-F | TTCGCACCGATAACAGGGCGTTTCGACTGAACTCACCTC |  |
| CAC-proto-spacer6-R | CAAG GAGGTGAGTTCAGTCGAAACGCCCTGTTATCGGTG |  |
| **For constructing PUC-two-protospacer** |  |  |
| Xba I -HindIII-proto-spacer5-1-F | CTAG GAAGGTTATATCCAGGGGGCAGGTTCAGCAGGTCCCCGCACA | |
| Xba I -HindIII-proto-spacer5-1-R | AGCT TGTGCGGGGACCTGCTGAACCTGCCCCCTGGATATAACCTTC | |
| Xba I -HindIII-proto-spacer4-F | CTAG GGAAGGAACGGAGGAATATAAGAACAAAAGCCCGCAGAGAAA | |
| Xba I -HindIII-proto-spacer4-R | AGCT TTTCTCTGCGGGCTTTTGTTCTTATATTCCTCCGTTCCTTCC |  |
| **For constructing KP8 Cas3-deletion** |  |  |
| Upstream of Cas3-F | GCGCGGTTGTCTCCTCTATT |  |
| Upstream of Cas3-R | GAAGCAGCTCCAGCCTACAC TGCGGCGACATCAAGGGAAT |  |
| Downstream of Cas3-F | GGACCATGGCTAATTCCCAT GCAGCTAACTACATGAATAC |  |
| Downstream of Cas3-R | CGGGCCAAACTGAAAGGCAG |  |
| Cam-FRT-F | GTGTAGGCTGGAGCTGCTTC |  |
| Cam-FRT-R | ATGGGAATTAGCCATGGTCC |  |
| **For constructing *E. coli* BW25113 CRISPR-deletion** |  |  |
| upstream of CRISPR-F | GGAATGGATGATAACGCCGC |  |
| upstream of CRISPR-R | GAAGCAGCTCCAGCCTACACTCATGCCAGCTATTTCCCGC |  |
| Kan with FRT-F | GTGTAGGCTGGAGCTGCTTC |  |
| Kan with FRT-R | CCATATGAATATCCTCCTT |  |
| downstream of CRISPR-F | AAGGAGGATATTCATATGGCAGCACCGGTAAATTGGCAC |  |
| downstream of CRISPR-R | TCGATCTAAACGCCCTGAACG |  |
| **For constructing pCRISPR-KP8** |  |  |
| PKD4-F | AAATATCGCTACCTGCCGCTCTAGCGATAT ACTGGGCTATCTGGACAAGG | |
| PKD4-R | GGGATTAAATAATAGAGGAGACAACCGCGC GCAAGATCCGCAGTTCAACC |  |
| KP8-CRISPR1-F | ATATCGCTAGAGCGGCAGG |  |
| KP8-CRISPR1-R | GAGGTACTTCGCCAGGCTTA |  |
| KP8-CRISPR2-F | GCTGTCGCAGAGAAAGCCTC |  |
| KP8-CRISPR2-R | GCGCGGTTGTCTCCTCTATT |  |

^a^ underlines sequences were the overlaps used for SOE-PCR or plasmid construction; the red bases were the PAM seque
