# Supplementary material for: The type I-E CRISPR-Cas system influences the acquisition of blaKPC-IncF plasmid in Klebsiella pneumonia
Source: Emerg Microbes Infect. 2020 May 20;9(1):1011–22. doi: 10.1080/22221751.2020.1763209 (PMC7301723; doi:10.1080/22221751.2020.1763209)
Supplement: Supplemental Material [file TEMI_A_1763209_SM1594.zip › Supplementary files/Table S3.docx]

**Table S3. MICs for clones isolated in the plasmid stable assay.**

| Strains | Passage Number | MIC (mg/L) | |  |
| --- | --- | --- | --- | --- |
|  |  | Imipenem | Chloramphenicol |  |
| ^1^p187-2-JS683 | 10 | ≤0.25 | —— |  |
| p187-2-JS681 | 10 | ≥16 | —— |  |
|  |  |  |  |  |
| ^2^pUC-proto-spacer6-KP8 | 3 | —— | 8 |  |
| pUC-proto-spacer6-JS687(KP8ΔCas3) | 3 | —— | ＞128 |  |
| pUC-Empty-KP8 | 3 | —— | ＞128 |  |
| pUC-Empty-JS687 | 3 | —— | ＞128 |  |
|  |  |  |  |  |
| pUC-proto-spacer6-JS683 | 6 | —— | 8 |  |
| pUC-proto-spacer6-JS681 | 6 | —— | ＞128 |  |
| pUC-Empty-JS683 | 6 | —— | ＞128 |  |
| pUC Empty-JS681 | 6 | —— | ＞128 |  |
|  |  |  |  |  |
| Control |  |  |  |  |
| JS683 | —— | ≤0.25 | 8 |  |
| JS681 | —— | ≤0.25 | 8 |  |
| KP8 | —— | —— | 8 |  |
| JS687 | —— | —— | 8 |  |

Note

^1^these results indicated the imipenem sensitivity in both two strains (JS683 and JS681) after 10 times passages in LB broth, which consistent with the Fig 3A

^2^these results indicated the chloramphenicol sensitivity in *K. pneumonia* (KP8 and JS687) after 3 times passages (Fig 3Bi) and corresponding MICs in *E. coli* (JS683 and JS681) after 6 times passages (Fig.3Bii)
