# Supplementary material for: The type I-E CRISPR-Cas system influences the acquisition of blaKPC-IncF plasmid in Klebsiella pneumonia
Source: Emerg Microbes Infect. 2020 May 20;9(1):1011–22. doi: 10.1080/22221751.2020.1763209 (PMC7301723; doi:10.1080/22221751.2020.1763209)
Supplement: Supplemental Material [file TEMI_A_1763209_SM1594.zip › Supplementary files/Table S4.docx]

**Table S4. Proto-Spacer sequences matching KP8 CRISPR**

| Name of proto-spacers | Sequence (5' to 3’) ^a^ | Plasmid | Accession number | GC Content |
| --- | --- | --- | --- | --- |
| proto-spacer1 | CAGACAGACAGCAGGCAGCAAACAGGGAAGAC | p187-2 | CP025468.1 | 56% |
| proto-spacer3 | GTGGTTTGTTACCGTGTTGTGTGGCAAAAAGC | p187-2 | CP025468.1 | 47% |
| protospacer4 | GAACGGAGGAATATAAGAACAAAAGCCCGCAG | p187-2 | CP025468.1 | 47% |
| proto-spacer5-1 | TT**ATAT**CCAGGGGGCAGGTTCAGCAGGTCCCC | p187-2 | CP025468.1 | 59% |
| proto-spacer5-2 | TT**AATA**CCAGGGGGCAGGTTCAGCAGGTCCCC | p0716-KPC | KY270849.1 | 59% |
| proto-spacer6 | CGATAACAGGGCGTTTCGACTGAACTCACCTC | p187-2 | CP025468.1 | 56% |
| proto-spacer8 | TCGTCTGAGTTCCGGCTTACGCCGTGCCGACA | p12139-KPC-2 | MF168403.1 | 63% |

^a^ red bases represent the mutations.
