# Supplementary figures and images for: The type I-E CRISPR-Cas system influences the acquisition of blaKPC-IncF plasmid in Klebsiella pneumonia
Source: Emerg Microbes Infect. 2020 May 20;9(1):1011–22. doi: 10.1080/22221751.2020.1763209 (PMC7301723; doi:10.1080/22221751.2020.1763209)

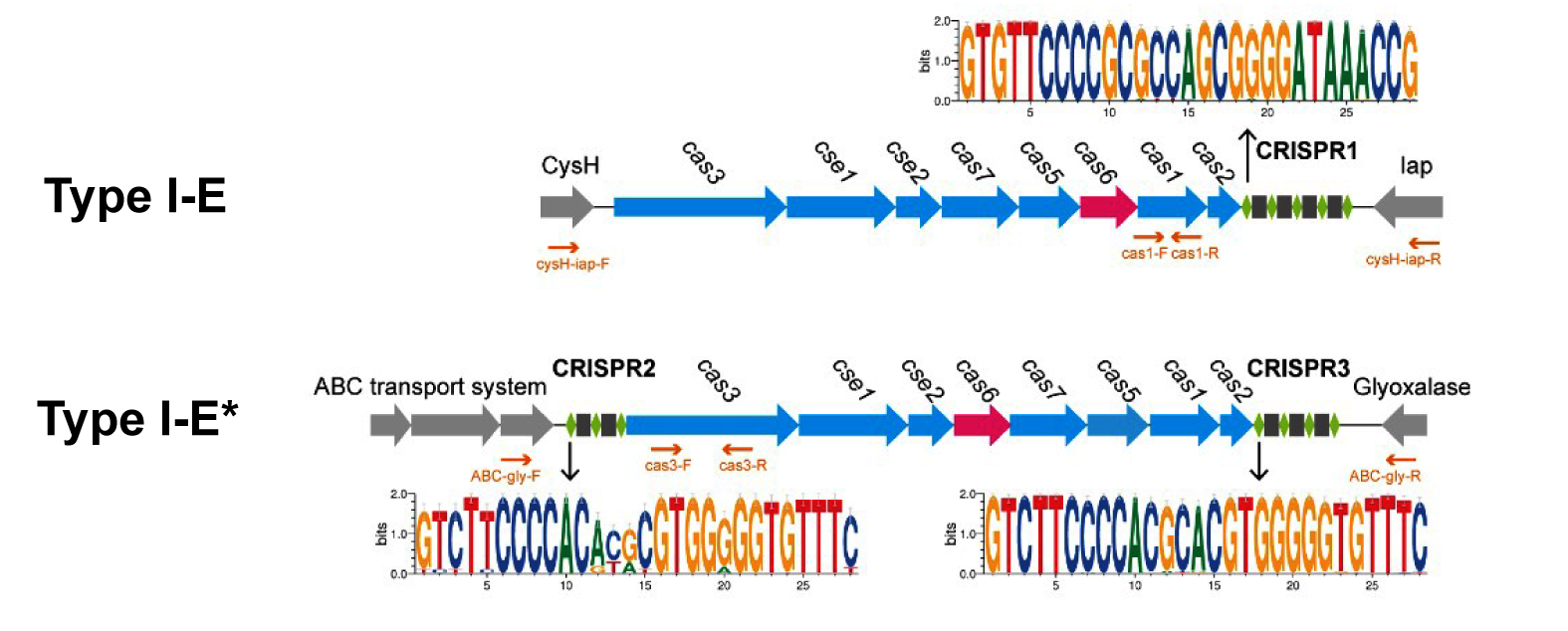

Supplement: Supplemental Material [file TEMI_A_1763209_SM1594.zip › Supplementary files/Fig S1.tif]

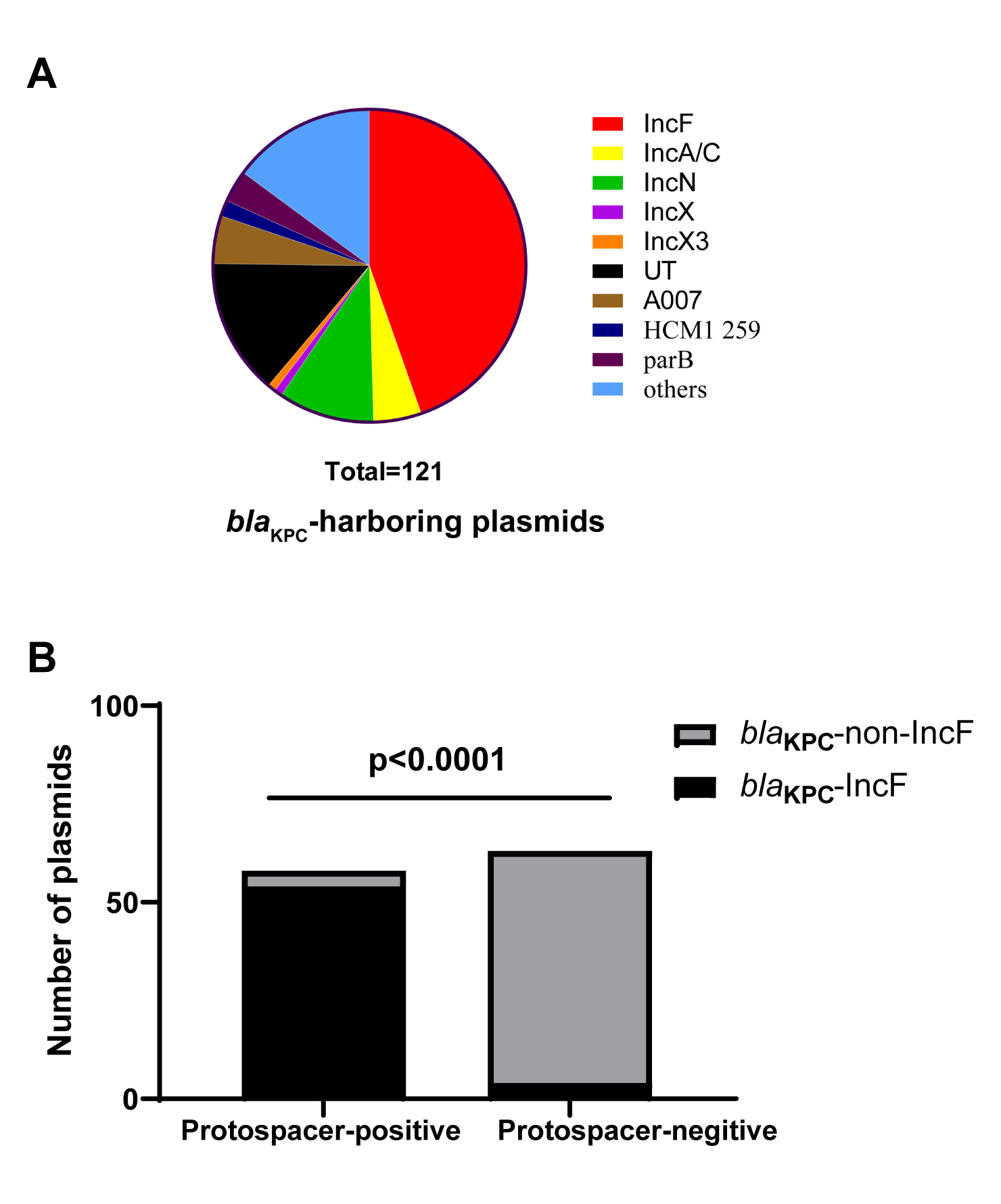

Supplement: Supplemental Material [file TEMI_A_1763209_SM1594.zip › Supplementary files/Fig S2.tif]

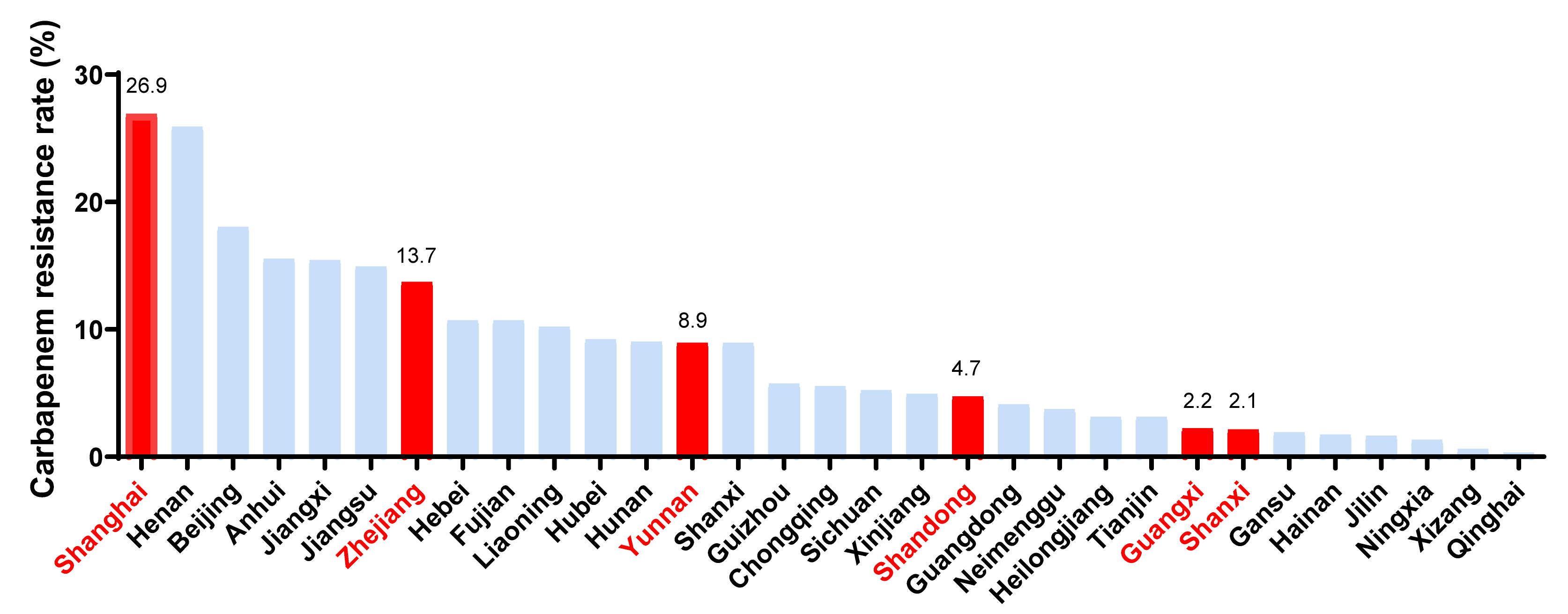

Supplement: Supplemental Material [file TEMI_A_1763209_SM1594.zip › Supplementary files/Fig S3.tif]

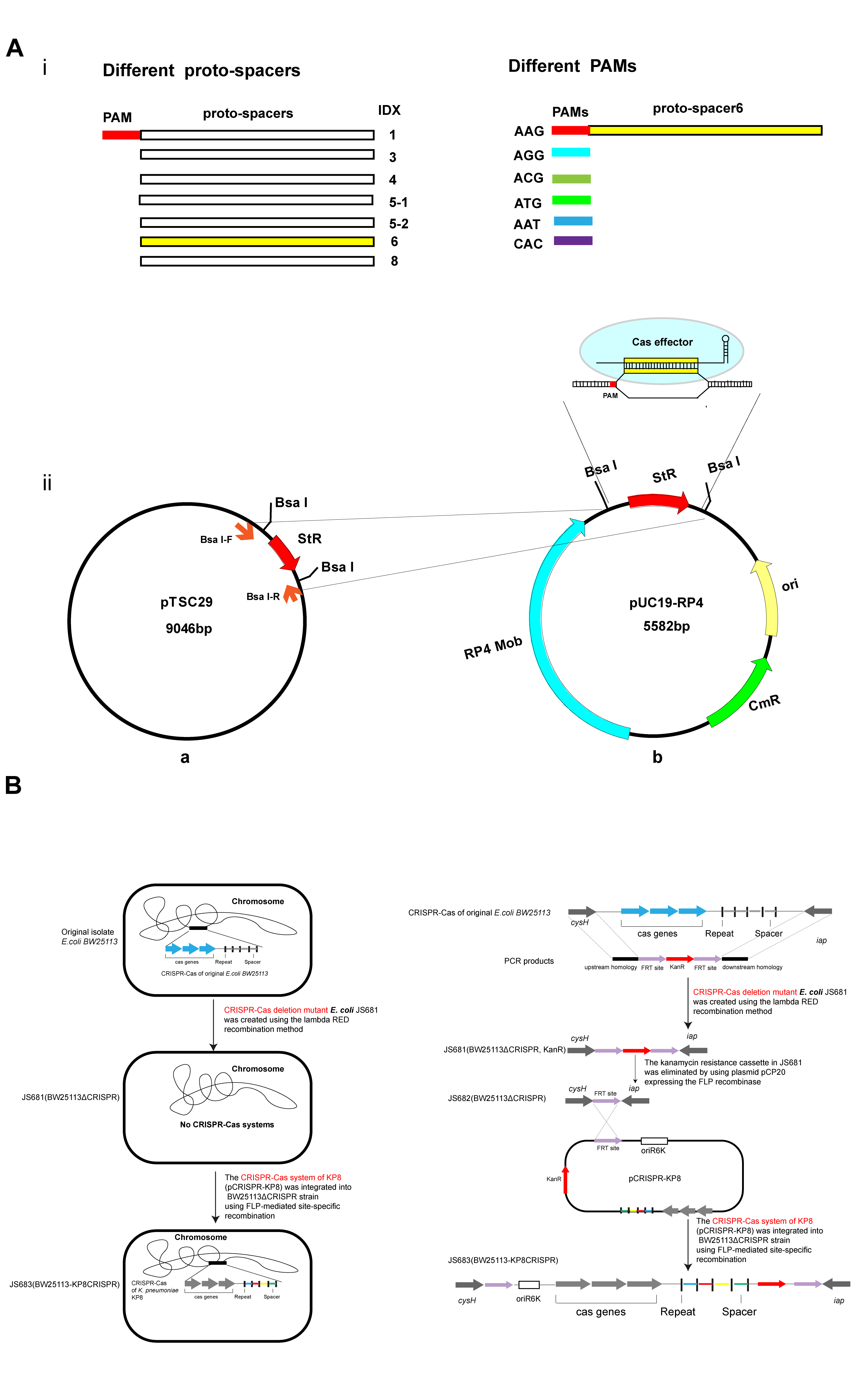

Supplement: Supplemental Material [file TEMI_A_1763209_SM1594.zip › Supplementary files/Fig_S4_5_21.tif]
