# Supplementary material for: The type I-E CRISPR-Cas system influences the acquisition of blaKPC-IncF plasmid in Klebsiella pneumonia
Source: Emerg Microbes Infect. 2020 May 20;9(1):1011–22. doi: 10.1080/22221751.2020.1763209 (PMC7301723; doi:10.1080/22221751.2020.1763209)
Supplement: Supplemental Material [file TEMI_A_1763209_SM1594.zip › Supplementary files/Table S1.doc]

**Table S1. Strains and plasmids used in this study**

| **Strains/plasmids** | **Relevant characteristics** | **Source or reference** |
| --- | --- | --- |
| ***K. pneumoniae*** |  |  |
| **KP8** | *K. pneumoniae* ST458(non-CC258) clinical isolates containing CRISPR-Cas system (containing spacer1, 3, 4, 5, 6 and 8). | **CP025636.1** |
| JS686 | KP8 derivative (CAM^R^) for allelic replacement of *cas3* protein with chloramphenicol resistance cassette flanked by FRT sites | This study |
| JS687 | KP8-ΔCas3 derivative, eliminates chloramphenicol resistance cassette by using FLP recombinase and contains an FRT site only | This study |
|  |  |  |
| ***E. coli*** |  |  |
| BW25113 | DE (araD-araB)567 lacZ4787(del)(::rrnB-3) lacIp-4000(lacI$^Q$) LAM- rph-1 DE(rhaD-rhaB)568 rrnB-3 hsdR514 | 23 |
| DH5αpir | endA1 hsdR17 glnV44 (= supE44) thi-1 recA1 gyrA96 relA1 φ80dlacΔ(lacZ)M15 Δ(lacZYA-argF) U169 zdg-232::Tn10 uidA::pir+ | Lab stock |
| S17-1 λpir | TpR SmR *recA, thi, pro, hsd*R-M+RP4: 2-Tc: Mu: KmR Tn*7* λ*pir* | 25 |
| MG1655 | F- lambda- ilvG- rfb-50 rph-1 | Lab stock |
| JS681 | BW25113 derivative (KAN^R^) for allelic replacement of E*. coli* CRISPR-Cas system with kanamycin resistance cassette flanked by FRT sites | This study |
| JS682 | BW25113-ΔCRISPR derivative, eliminates kanamycin resistance cassette by using FLP recombinase and contains an FRT site only | This study |
| JS683 | BW25113-ΔCRISPR: FRT derivative (KAN^R^), contains CRISPR-Cas of KP8 integrated in the FRT site | This study |
| JS531 | DH5α contains plasmid p187-2, an IncFII conjugative plasmid harbouring *bla*KPC and matches for spacer1, 3, 4, 5 and 6 recovered in K. pneumoniae. Accession number of p187-2: CP025468.1 | This study |
|  |  |  |
| **Plasmids**  **p187-2** | an IncFII conjugative plasmid harbouring *bla*_KPC_ and matches for spacer1, 3, 4, 5 and 6 recovered in *K. pneumoniae* | **CP025468.1** |
| pKD4 | AMP^R^ KAN^R^, kanamycin cassette flanked by FRT sites template, pir-dependent | 23 |
| pKD3 | AMP^R^ CAM^R^, chloramphenicol cassette flanked by FRT sites template, pir-dependent | 23 |
| pKD46 | AMP^R^, λ Red recombinase expression, 30°C | 23 |
| pKOBEG | Apr^R^, λ Red recombinase expression, 30°C | Lab stock |
| pCP20 | AMP^R^ CAM^R^, FLP recombinase expression, 30°C | 25 |
| pCP20-KAN | AMP^R^ KAN^R^, FLP recombinase expression, 30°C | This study |
| pUC19 | AMP^R^, ori pMB1 | Lab stock |
| pUC19-CAM | CAM^R^, ori pMB1 | This study |
| pUC19-Apr | Apr^R^, ori pMB1 | This study |
| pJTOOL-3 | CAM^R^, RP4-specific Mob site template | 25 |
| PTSC29 | STR^R^ CAM^R^, *Bsa I* restriction site template | Lab stock |
| pCRISPR-KP8 | KAN^R^, pKD4 derivative containing CRISPR-Cas system of KP8 and one FRT site, pir-dependent | This study |
| pUC-RP4 | CAM^R^, ori pMB1, pUC19 derivative, inserts *Bsa I* restriction site and RP4-specific Mob site by In-Fusion Cloning | This study |
|  |  |  |
